# Synthesis of Novel 2-Thiouracil-5-Sulfonamide Derivatives as Potent Inducers of Cell Cycle Arrest and CDK2A Inhibition Supported by Molecular Docking

**DOI:** 10.3390/ijms222111957

**Published:** 2021-11-04

**Authors:** Samar S. Fatahala, Amira I. Sayed, Shahenda Mahgoub, Heba Taha, Mohamed-I kotb El-Sayed, Mohamed F. El-Shehry, Samir M. Awad, Rania H. Abd El-Hameed

**Affiliations:** 1 Pharmaceutical Organic Chemistry Department, Faculty of Pharmacy, Helwan University, Helwan 11795, Egypt; amiraelsaedy@yahoo.com (A.I.S.); Samir.Awad@pharm.helwan.edu.eg (S.M.A.); zeiadomar@yahoo.com (R.H.A.); 2 Department of Biochemistry and Molecular Biology, Faculty of Pharmacy, Helwan University, Helwan 11795, Egypt; shahenda.mahgoub@pharm.helwan.edu.eg (S.M.); heba.taha@pharm.helwan.edu.eg (H.T.); mohamed.kotb71524@gmail.com (M.K.E.-S.); 3 Pesticide Chemistry Department, National Research Centre, Dokki 12622, Egypt; moh_elshehry2000@yahoo.com

**Keywords:** sulfonamides, cytotoxicity, apoptosis, cell cycle, CDK2, molecular docking

## Abstract

In an effort to discover potent anticancer agents, 2-thiouracil-5-sulfonamides derivatives were designed and synthesized. The cytotoxic activity of all synthesized compounds was investigated against four human cancer cell lines viz A-2780 (ovarian), HT-29 (colon), MCF-7 (breast), and HepG2 (liver). Compounds **6b,d**–**g**, and **7b** showed promising anticancer activity and significant inhibition of CDK2A. Moreover, they were all safe when tested on WI38 normal cells with high selectivity index for cancer cells. Flow cytometric analysis for the most active compound **6e** displayed induction of cell growth arrest at G1/S phase (A-2780 cells), S phase (HT-29 and MCF-7 cells), and G2/M phase (HepG2 cells) and stimulated the apoptotic death of all cancer cells. Moreover, **6e** was able to cause cycle arrest indirectly through enhanced expression of cell cycle inhibitors p21 and p27. Finally, molecular docking of compound **6e** endorsed its proper binding to CDK2A, which clarifies its potent anticancer activity.

## 1. Introduction

Cancer is a life-threatening disease and a major global health problem as it is the second leading cause of death in the world [1]. Current cancer treatment, including chemotherapy and radiotherapy, has systemic toxicity and high drug resistance. Therefore, there is a need to develop new anti-cancer agents [2,3]. Literature survey indicated that pyrimidine analogs are of considerable interest in drug discovery [4,5,6,7,8]. They possess remarkable biological activity especially as anti-cancer and anti-viral agents due to their resemblance to cellular pyrimidine bases involved in nucleic acids formation. The synthetic pyrimidine analog 5-fluorouracil (5-FU) is an antimetabolite drug that has been widely used for the treatment of various types of cancer like colon, breast, and other cancers through meddling with the cellular biosynthetic activity via inhibiting thymidylate synthase or by misincorporation of its metabolites into nucleic acid [9]. Moreover, 5-FU is considered a cell cycle-phase-dependent anticancer drug [10] and studies have revealed that 5-FU interfered with the cell cycle at the G1 and S phases [11,12].

**Figure 1:**
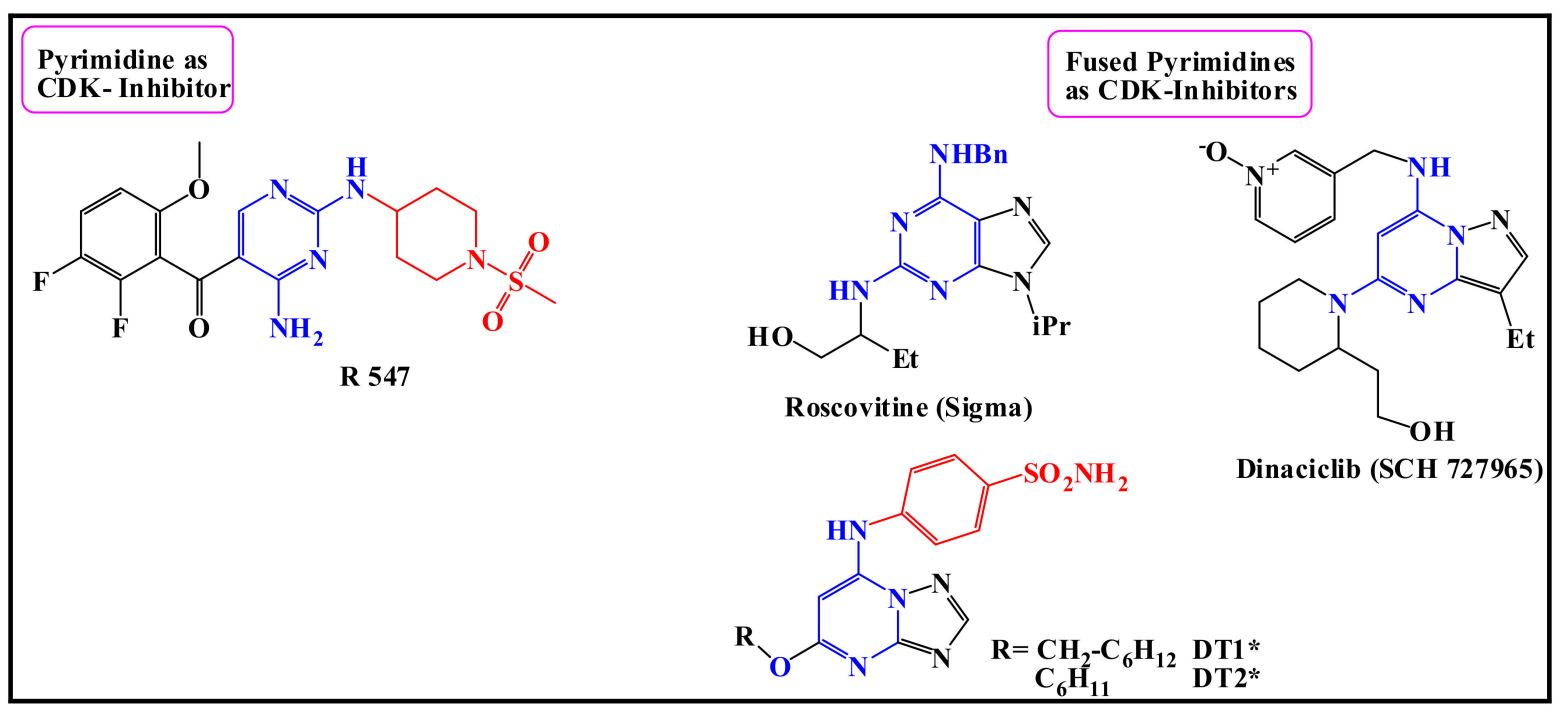
Structures of some CDK inhibitors [21] containing; pyrimidine [22], fused pyrimidine compounds [23,24,25], and pyrimidine bearing sulfonamide moiety [26,27].

However, the extensive utilization of 5-FU in chemotherapy has resulted in the development of drug resistance [13,14]. During the journey to use combination therapy with 5-FU, studies were performed using computer-aided screening to reveal molecular targets for 5-FU. A study by Yata et al. used in silico methods and reconfirmed that cyclin-dependent kinase-2 (CDK2) can be a potential molecular target for 5-FU with a significant binding affinity [15]. Moreover, recently, 5-FU compounds bearing sulfonamide and Sulfone groups have shown anti-CDK2, CDK9 promising activities [16]. Furthermore, pyrimidine analogs have been developed as cyclin-dependent kinase inhibitors [17,18], like CDK2A inhibitors [19] or checkpoint kinase inhibitors [20], as revealed in Figure 1.

Cyclin-dependent kinases (CDKs) belong to a family of Ser/Thr protein kinases that are essential for cell progression regulation through cell cycle G/S and G2/M1 phases [28] that control cell development, via controlling the DNA replication of chromosomes [29,30,31]. However, deregulation of CDKs may result in uncontrolled cell proliferation, as well as genomic and chromosomal instability and thus contributing to both cancer progression and aggressiveness [32].

There are 20 different CDKs in the human kinome viz CDK1 to CDK20 [33]. Studies showed overexpression of CDK1 in breast cancer, and loss of expression of CDK inhibitory proteins (CKI) by mutational or epigenetic modifications, in melanoma, lung, and breast cancers. Moreover, several cancers are exclusively dependent on CDKs and therefore are selectively sensitive to their inhibition [34]. Therefore, synthetic inhibitors of CDK activity exist as a plausible tactic in the advance of new cancer treatments [35].

**Figure 2:**
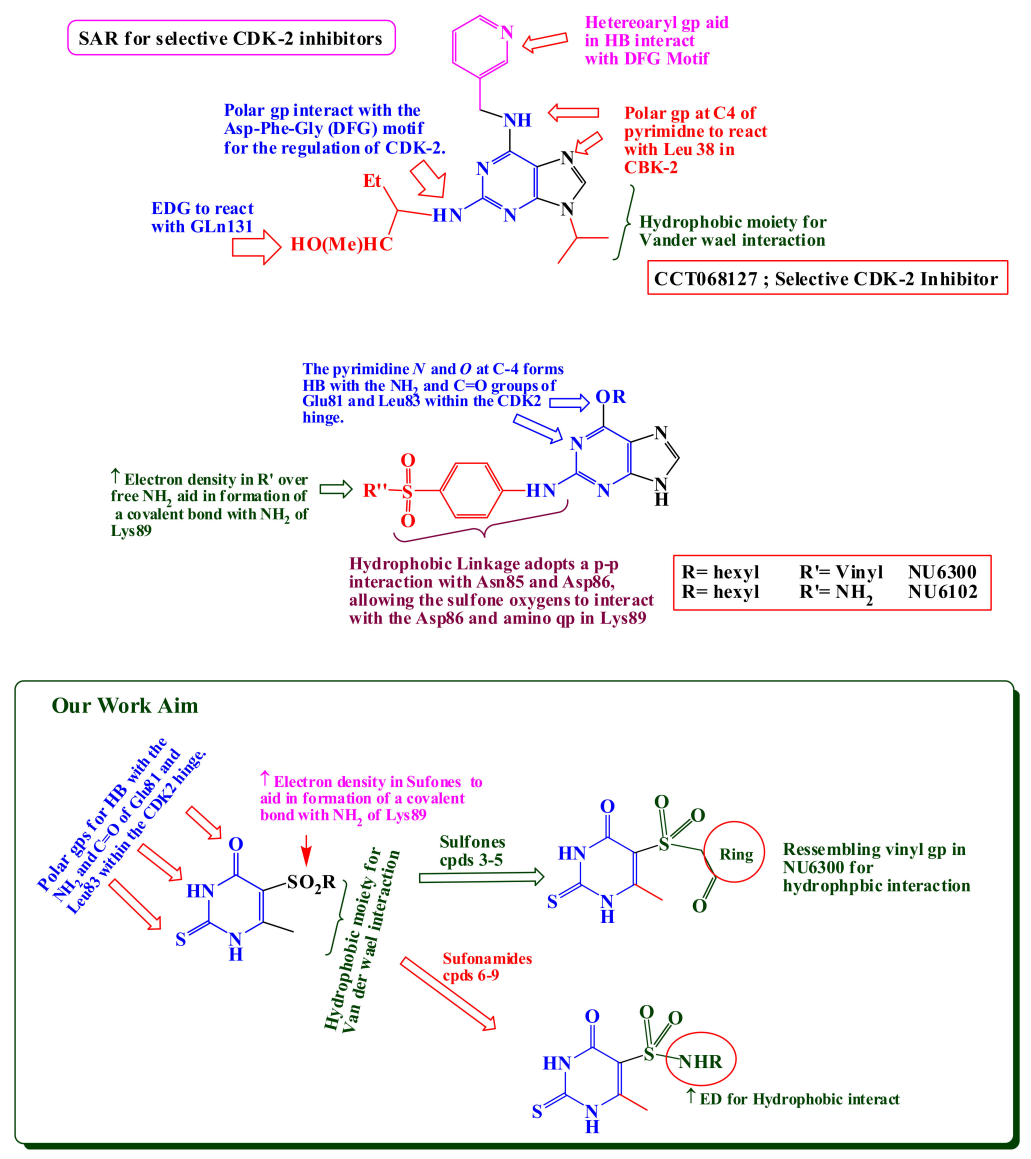
Structure-activity of some selected CDK-2 inhibitors [5,6,7,8,36,37] and structure design of our newly synthesized compounds **3**–**9**.

Enthused by the aforementioned studies, we were endeavored to design and synthesize novel sulfonamides and sulfones, aiming to develop new potent anticancer agents with potential CDK inhibition activity (Figure 2). The synthesized compounds were screened for their cytotoxicity to investigate their anti-cancer activity against; four human A-2780 (ovarian), HT-29 (colon), MCF-7 (breast), and HepG2 (liver) cancer cell lines and subsequent testing of CDK2 inhibition activity of the most active derivatives.

## 2. Results and Discussion

### 2.1. Chemistry

An overview of the literature survey on the importance of 2-thiouracils, sulfonamides, and 2-thiouracil-5-sulfonamides in biological systems [38], prompted us to design and synthesize a new class of 5-substituted-2-thiouracil derivatives such as the sulfonamide isosteres [38,39].

The preparation of sulfonamides is based on the reactions of chlorosulfonates [38,40]. Mainly, pyrimidine may not undergo the reaction of chlorosulfonate, because of the inhibitory effect of two atoms of nitrogen. However, incorporation of SH, OH, and methyl groups at the second, fourth, and sixth positions increased the reaction of the pyrimidine ring towards chlorosulfonide formation [41].

**Scheme 1:**
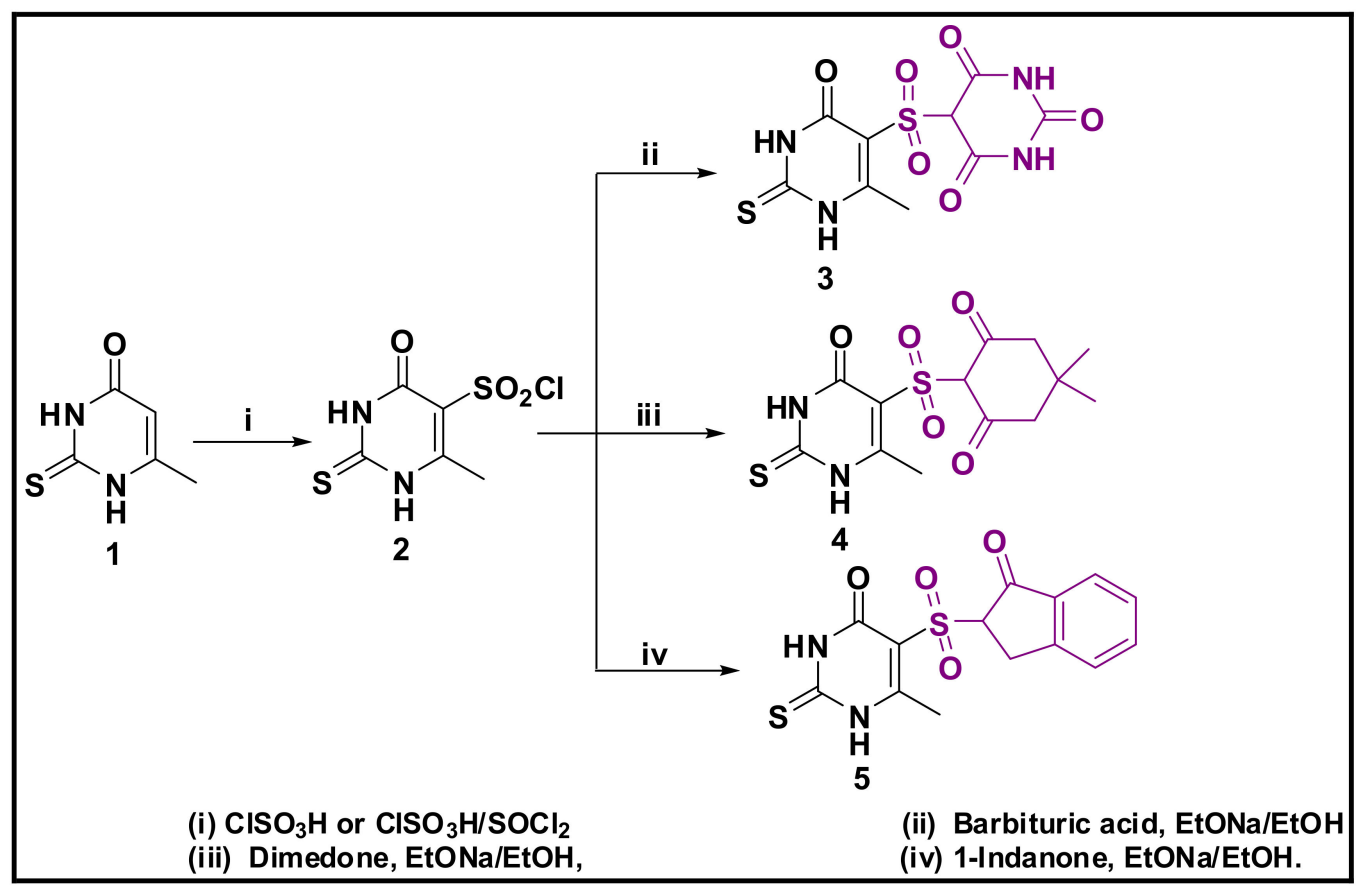
Preparation of Sulfone derivatives **3**–**5**.

The reaction of compound (**1**) with chlorosulfonide was performed by refluxing with a mixture of chlorosulfonic acid and thionyl chloride at 120 °C, offered compound (**2**) in good yield. This active sulfonyl chloride has undergone a wide variety of reactions. First, with the active methylene, namely, barbituric acid and demidone and 1-indanone in sodium ethoxide/ethanol; giving the sulfone derivatives **3**, **4,** and **5**, respectively. The synthetic method for the preparation of sulfonylpyrimidine-4-one derivatives (**2**–**5**) is revealed in Scheme 1.

**Scheme 2:**
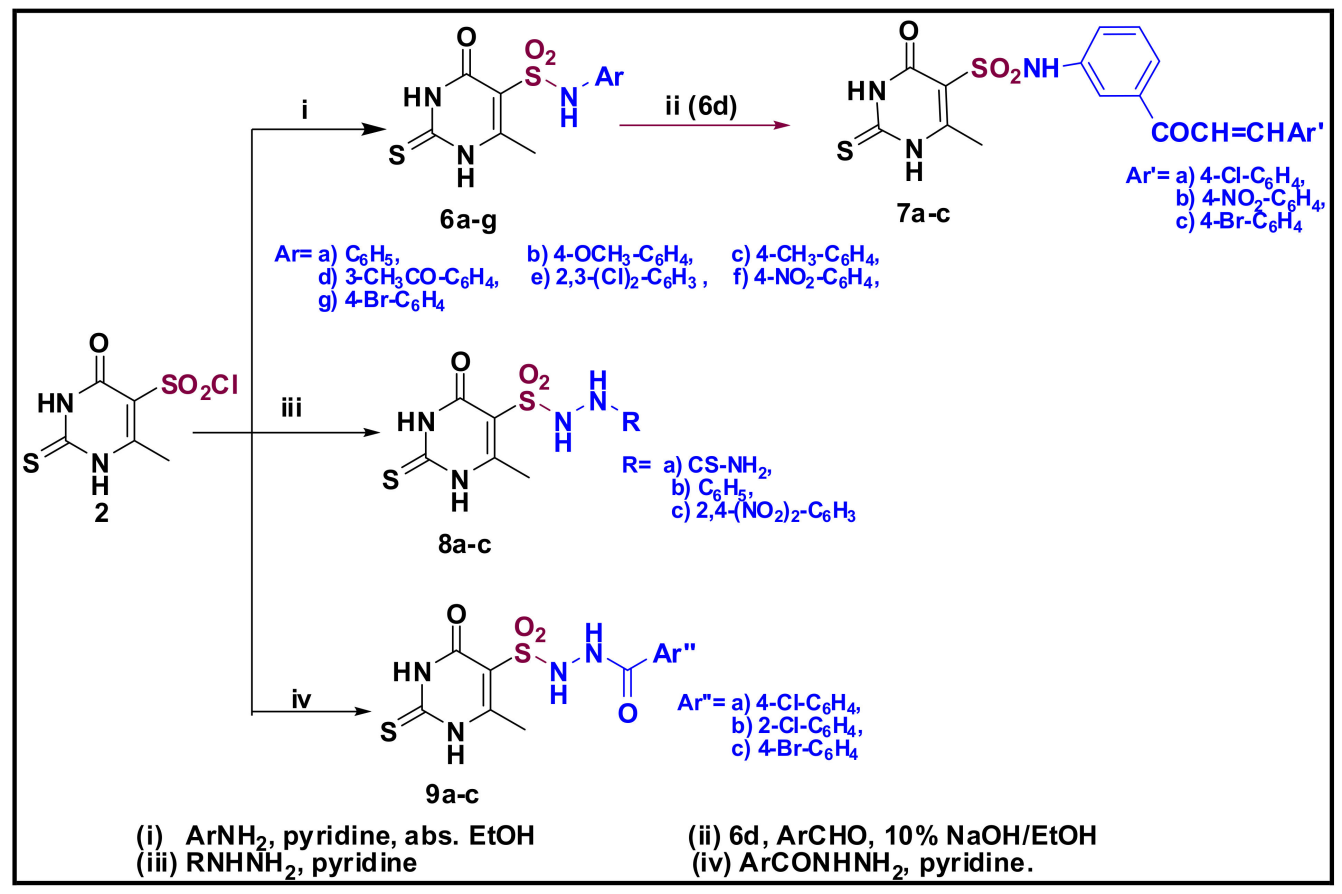
Synthesis of Sulfonamide derivatives (compounds **6**–**9**).

Scheme 2 revealed the reaction of sulfonyl chloride (**2**) with a series of aromatic amines (i.e., aniline, 4-methoxyaniline, 4-methylaniline, 3-acetylaniline, 3,4-dichloroaniline, 4-nitroaniline, and 4-bromoaniline) in absolute ethanol containing pyridine as an acid scavenger that provided the sulfonamide derivatives **6a**–**g** through which, the **6d** compound underwent further Claisen-Schmidt condensation with some aromatic aldehydes (namely, 4-chloroaldehyde, 4-nitro aldehyde, and 4-bromoaldehyde), resulting in the unsaturated α-carbonyl compounds (chalcones) **7a**–**c**. Moreover, sulfonyl chloride **2** also reacted with thiosemicarbazide, hydrazine, and hydrazide derivatives to provide the corresponding compounds **8a–c** and **9a**–**c.** The structures of all the newly synthesized compounds (**2**–**9**) were in complete accordance with the achieved spectral and elemental analyses data.

### 2.2. Biological Evaluation of the Cytotoxic Effect of 2-Thiouracils Derivatives

5-fluorouracil; a halogenated metabolite is one of the primary metabolites considered in cancer therapy and it is one of the most active pyrimidine derivatives [38,42]. Moreover, studies of other halogenated uracylates have become an alternative for naturally occurring uracil. Subsequently, 5-FU acts like uracil in its biochemical properties [14,43,44], while 5-iodo- or 5-bromo uracil are more like thymine. Halogenated uracil can be easily incorporated into DNA as a pseudo-metabolite [14,21,42], indicating the importance of C-5 substitutes size in determining the metabolic fate of a compound and whether or not it may be incorporated into DNA [45]. Usually, DNA-directed effects are exhibited in the S phase, while RNA-directed ones are manifested in the G1 phase [46]. 5-FU was found to block G1 and S phases of the cell cycle [11,12]. Hence, several structural adjustments have been made to fit this point, among these 2-thiouracil-5-sulfonamides, which have been designed and screened for their anticancer activities [38,41,47]. Thus, in this research we were endeavored to perform a cytotoxic initial screening of a series of 2-thiouracils against a panel of four different cancer cell lines, viz A-2780, HT-29, MCF-7, and HepG2, using 5-FU as the reference cytotoxic drug. The cytotoxic activities expressed as the median growth inhibitory concentration (IC_50_) are provided in Table 1. 

Results showed a potent growth inhibitory activity against MCF-7 and HT-29 cell lines, and fair activity against A-2780 and HepG2 cancer cells. Nevertheless, most of the synthesized compounds showed higher cytotoxicity against HepG2 cells than that of **5-FU**. It should be noted that compound **6e**, bearing the 2,3-dichlorophenyl, showed the most active inhibitory activity against the four cancer cell lines. While chalcone derivatives **7**, 5-sulfonohydrazides **8**, and **9** showed less anti-cancer activity. In the case of sulfone derivatives **3**, **4**, and **5**, there was low or no anticancer activity. 

**Figure 3:**
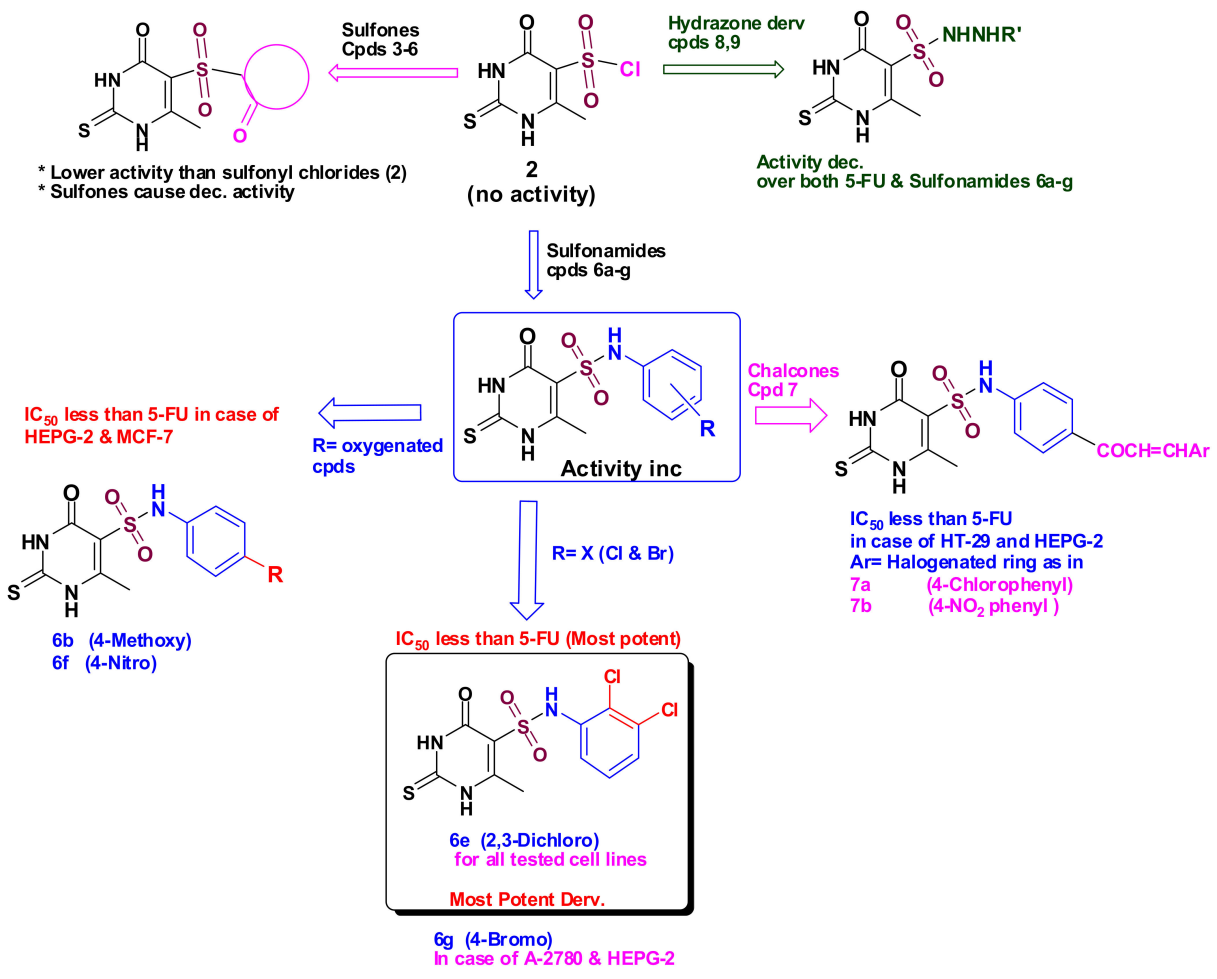
SAR for all tested compounds (**3**–**9**), compared to 5-FU.

Regarding SAR studies, we found that sulfones **3**, **4**, and **5** (containing no SO_2_NH group) were inactive towards the four used cell lines. Also, sulfonyl chloride **2** (the starting compound) showed low anticancer activity against those cell lines. On the other hand, sulfonamides **6b**, **6e**, and **6g** (bearing 4-methoxyphenyl, 2,3-dichlorophenyl, and 4-bromophenyl, respectively) were the most potent against the used cancer cell lines, suggesting that the presence of sulfonamide group (-SO_2_NH-) is crucial for cytotoxic activity. Yet, further modification of the -SO_2_NH- group (for example, -SO_2_NHNH- as in compounds **8** and **9**) diminished the anticancer activity, as revealed in Figure 3. 

Based on the cytotoxicity results, the compounds showing promising cytotoxicity compared to **5-FU** were selected to carry out a screening for their cytotoxic effects against normal human cell line WI38, and the selectivity index was calculated to test their safety on normal cells. As shown in Table 2, most of the selected compounds were selective to the cancer cells; however, compounds **6b, 6e**, and **6g** were the most selective ones towards cancer cells with the highest selectivity index. The most potent compound **6e** showed the greatest selectivity towards MCF-7 cells (SI, 36.05) followed by HT-29 cells (SI, 34.40).

### 2.3. Inhibitory Activities of the Most Active Cytotoxic Compounds against CDK2 Protein Kinase

The promising cytotoxic effects of compounds **6b,d**–**g**, and **7a**–**c**, especially **6e**, encouraged us to study their further inhibitory activities against CDK2 protein kinase. As shown in Table 3, compounds **6b**, **6g**, and **6e** exhibited good inhibitory activity against CDK2. Furthermore, compound **6e** showed the best inhibitory activity against CDK2 which was comparable to that of the known kinase inhibitor, Roscovitine. On the other hand, compounds **7a** and **7c** exhibited the least inhibitory activity. 

### 2.4. Cellular Mechanism of Action

As a result of the encouraging cytotoxic effect and the CDK2 inhibitory activity exhibited by the dichlorophenol derivative **6e,** it was selected for further assessment for its cellular mechanism of action.

#### 2.4.1. Annexin V-FITC/PI Apoptosis Assay

Staining with Annexin V-FITC and propidium iodide (PI) was applied to confirm apoptosis induction on the four cell lines. Phosphatidylserine (PS) is a phospholipid that is normally kept in the inner membrane of the viable cells. An early event in apoptosis is the translocation of PS to be exposed on the membrane surface, where it functions as a signal for the attack of phagocytes. Annexin V possesses a very high affinity for membranes exposing the negatively charged PS and thus can be used as marker of early apoptosis [48]. Dual staining with PI and annexin V-FITC can discriminate between viable, early, and late apoptotic, as well as necrotic cells by alterations in plasma membrane integrity and permeability [49]. Annexin-V produces green fluoresces when it exclusively binds to PS exposed on the surface of the apoptotic cells. Propidium iodide shows red fluorescence on staining DNA of late apoptotic and necrotic cells [50]. Thus, early apoptotic cells are Annexin V-FITC ^+^/PI^−^, while late apoptotic or secondary necrotic cells are Annexin V-FITC^+^/PI^+^ [51]. The effect of compound **6e** on normal cell cycle profile and induction of apoptosis was investigated when cells were treated with **6e** at its IC_50_ values for 48 h. Results showed that treatment of A-2780, HT-29, MCF-7, and HepG2 cells with **6e** amplified the percentage of Annexin-V FITC in early and late apoptosis. However, **6e** mainly induced the late stage of apoptosis in all treated cancer cells, (Table 4). Therefore, **6e** may exert its cytotoxic activity by induction of apoptosis. 

#### 2.4.2. Cell Cycle Arrest 

**Figure 4:**
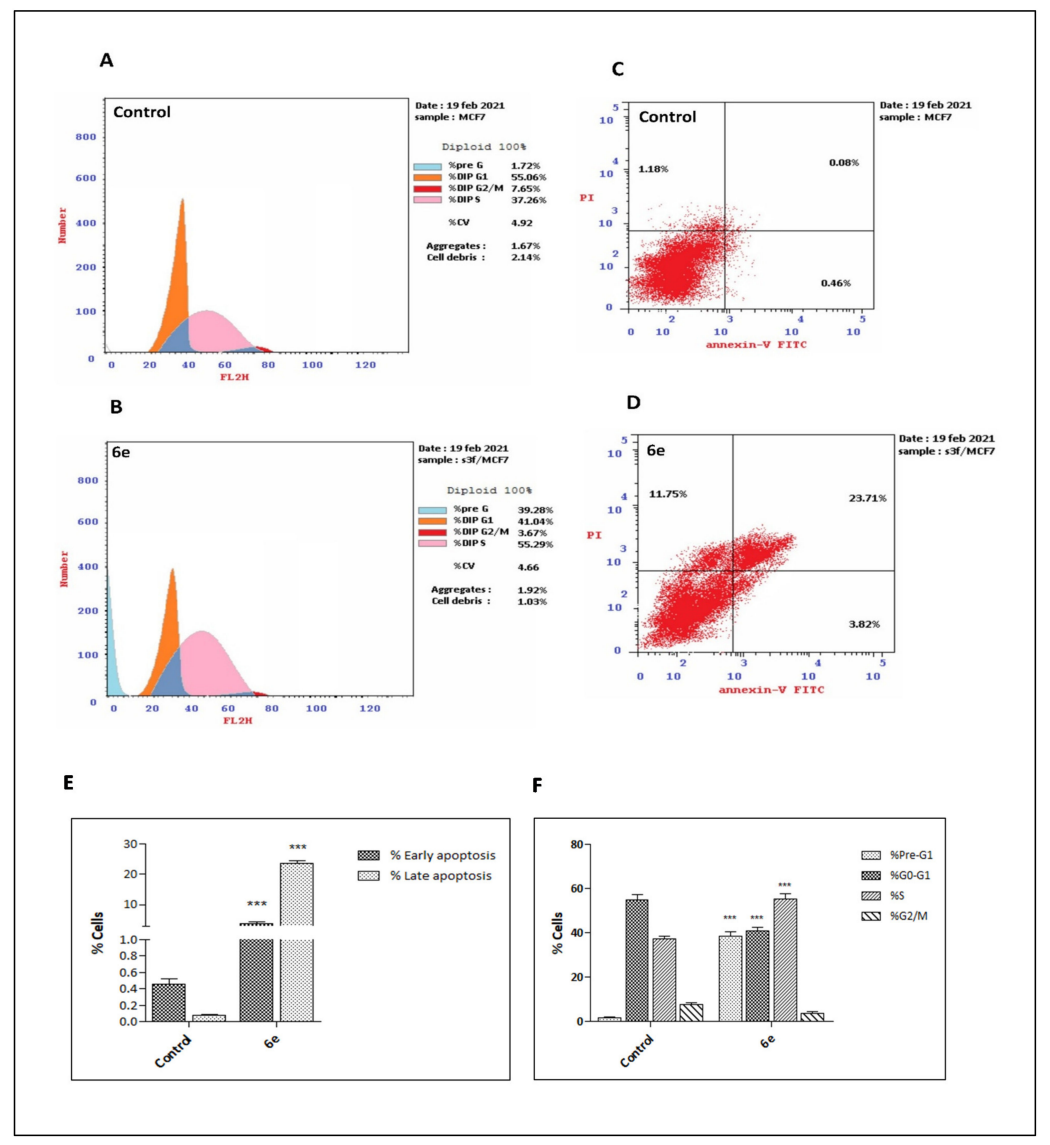
**(A** and **B):** DNA-flow cytometry analysis for MCF-7 cells treated with compound **6e** for 48h at its IC_50_. 0.1% DMSO was used as a control. **(C** and **D):** Induction of apoptosis by compound **6e**. Cells were analyzed by annexin V/PI staining after exposure to **6e** for 48 h. The percentage of cells enduring apoptosis is described as the sum of early apoptotic (annexin V^+^/PI^−^) and late apoptotic (annexin V^+^/PI^+^) cell percentages. **(E** and **F):** Bar charts showing early and late apoptotic cells and comparative analysis of percentage of cells in each cell cycle phase after treatment with compound **6e** for 48 h. Values are displayed as mean ± SD of three individual experiments. *** *p* < 0.001 versus the control.

To verify the CDK2 inhibition activity demonstrated by compound **6e**, cell cycle analysis was performed. DNA structure checkpoints are one of the key aspects of cell cycle regulation, which cause cell cycle arrest at diverse phases (G1, S, G2, and M) in response to DNA damage or incomplete replication [52]. CDKs are protein kinases involved in regulating the cell cycle as they control the cell cycle progression from one phase to the later. Our results showed that treatment of all four cancer cell lines with compound **6e** resulted in interfering with cell cycle distribution causing significant cell growth arrest at G1/S phase (A-2780 cells), S phase (HT-29 and MCF-7 cells), and G2/M phase (HepG2 cells), compared to the control, as revealed in Figure 4 and Supplementary Materials. These results were consistent with its CDK2 inhibitory effect as cell cycle arrest occurred at CDK2 regulated checkpoints. Furthermore, this data also indicated the apoptotic role of compound **6e** in cancerous cells. 

### 2.5. Expression Levels of Cyclin-Dependent Kinase Inhibitor Proteins p21 and p27

**Figure 5:**
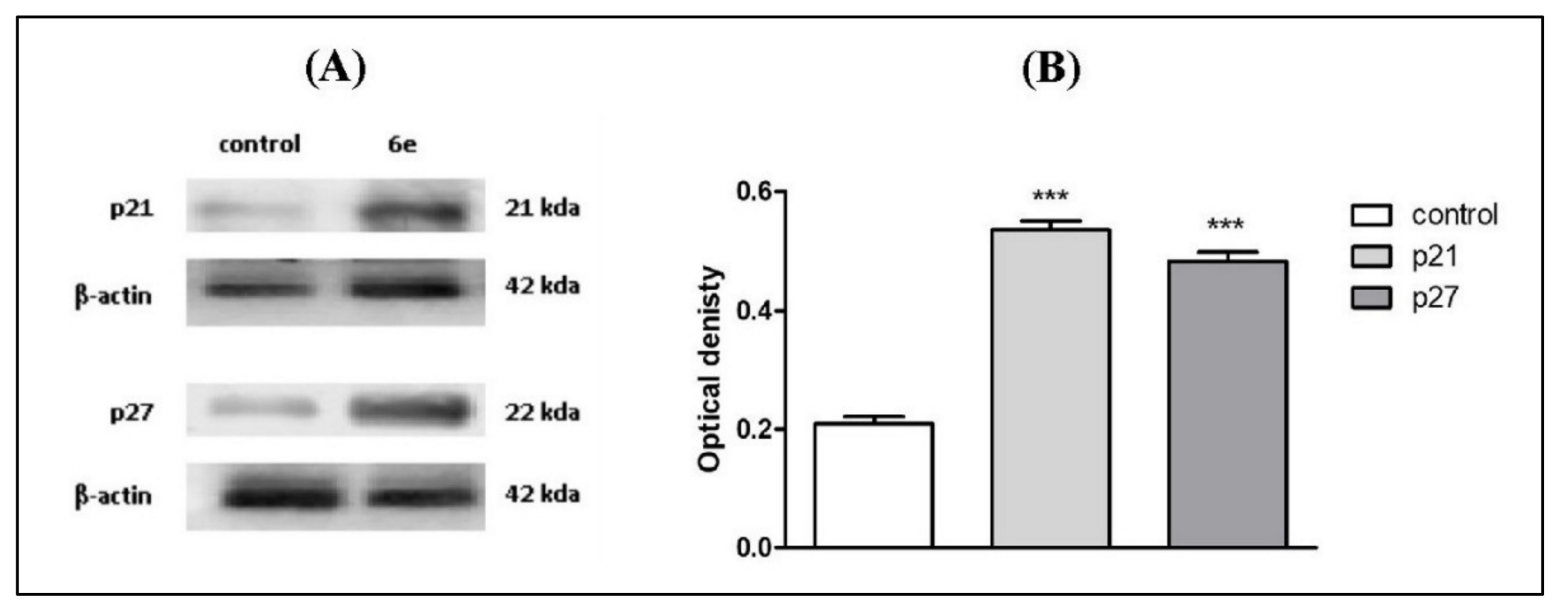
Effect of compound **6e** on cell-cycle regulatory proteins p21 and p27 in MCF-7 cells treated with IC_50_, 1.67 μM for 48 h measured by western blot. (**A**) One of three repeated experiments is shown. (**B**) Bar chart represents the quantification of the protein expression level of p21 and p27 compared to control. *** Significantly different from control at *p* < 0.001.

Finally, we tested the ability of **6e** to cause cell cycle arrest indirectly through enhancing the expression of cyclin-dependent kinase inhibitors. Cyclin-dependent kinase inhibitors p21 and p27 are proteins that bind to and inhibit the activity of CDK2/cyclin E. Those proteins play a major role in cell cycle control and are mainly regulated at the transcriptional level, as their induction mainly leads to cell cycle arrest [53]. Moreover, tumors with low levels of p27 had higher cyclin E/CDK2 activities than those with high p27 levels [54]. Furthermore, high levels of p21 and p27 were found to inhibit the cell cycle via targeting CDK2 [55]. Therefore, the expression of p21 and p27 were examined in MCF-7 cells treated with **6e** at IC_50_ (1.67 μM) by Western blot analysis since it was the most sensitive cell line to **6e**. Exposure to compound **6e** resulted in an elevation in both p21 and p27 protein levels in MCF-7 cells compared to the control group (Figure 5A). The obtained data revealed that the protein expression levels of p21 and p27 in **6e** treated MCF-7 cells were increased by about 2.6- and 2.2-fold, respectively, compared to control (Figure 5B). Our results agree with a previously published work, which showed that some pyrimidine derivatives were able to cause cell cycle arrest indirectly through enhancing the expression level of p21 protein by 1.7-fold [3]. Furthermore, another study on ovarian A-2780 tumor cells demonstrated a four-fold increase in the protein level of p27 induced by DAP, a cisplatin analogue, and the increase was comparable to or higher than that of p21 [56]. In the present study, compound **6e** was able to cause cell cycle arrest indirectly through enhancing the expression of cyclin-dependent kinase inhibitors p21 and p27, in addition to its direct inhibition of CDK2 activity.

### 2.6. Molecular Docking against CDK2

**Figure 6:**
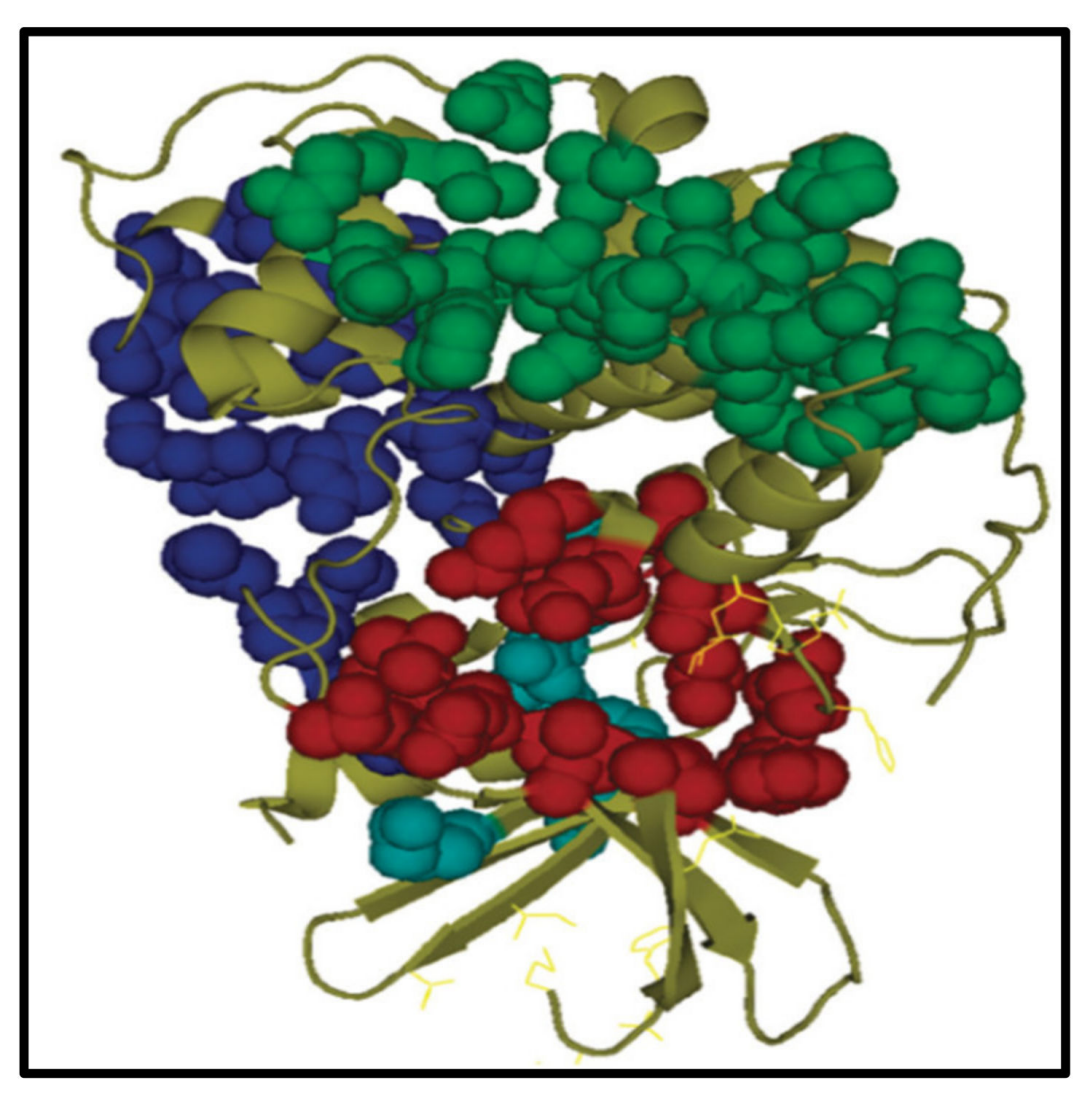
The four binding sites of CDK2A. ATP attachment pocket (site I) indicated in red, the second II and third sites III are indicated by green and blue, respectively. The allosteric site (fourth site IV) is indicated in cyan.

Fostered by the promising activities of compounds (**6b**,**d–g**, and **7b**) as anticancer agents, a molecular docking study was performed using the MOE 2014.09 software. The potential binding patterns for active compounds within the active site of the CDK2-ATP binding site were supposed [57]. Here, the binding modes of the highly active compounds were calculated against the CDK2-ATP binding site [58,59,60]. The docking results were linked to the conveyed ligand, 2,4,6-Trioxo-1-phenyl-hexahydropyrimidone- 5-carboxamide (TPHP). Concisely, CDK2 has 298 amino acid residues counting four binding sites; the adenosine triphosphate (ATP) binding pocket (Site I), two non-competitive binding sites (Sites II and III), and allosteric binding site (Site IV), as revealed in Figure 6.

The ATP-binding site is the best binding site, where the contraindications are linked to the challenge with ATP [26,27]. It is situated at a deep cleft among the amino- and carboxy-terminal lobes. It is highly characterized by a hydrophobic property. The notable effects of link stabilization within this site are hydrogen bond interactions formed with the hinge region including Lys33, Lys129, and Gln131, as well as the hydrophobic interactions (Gly13) [57]. The most important residues present in the CDK2-ATP binding pocket are revealed in Table 5.

**Figure 7:**
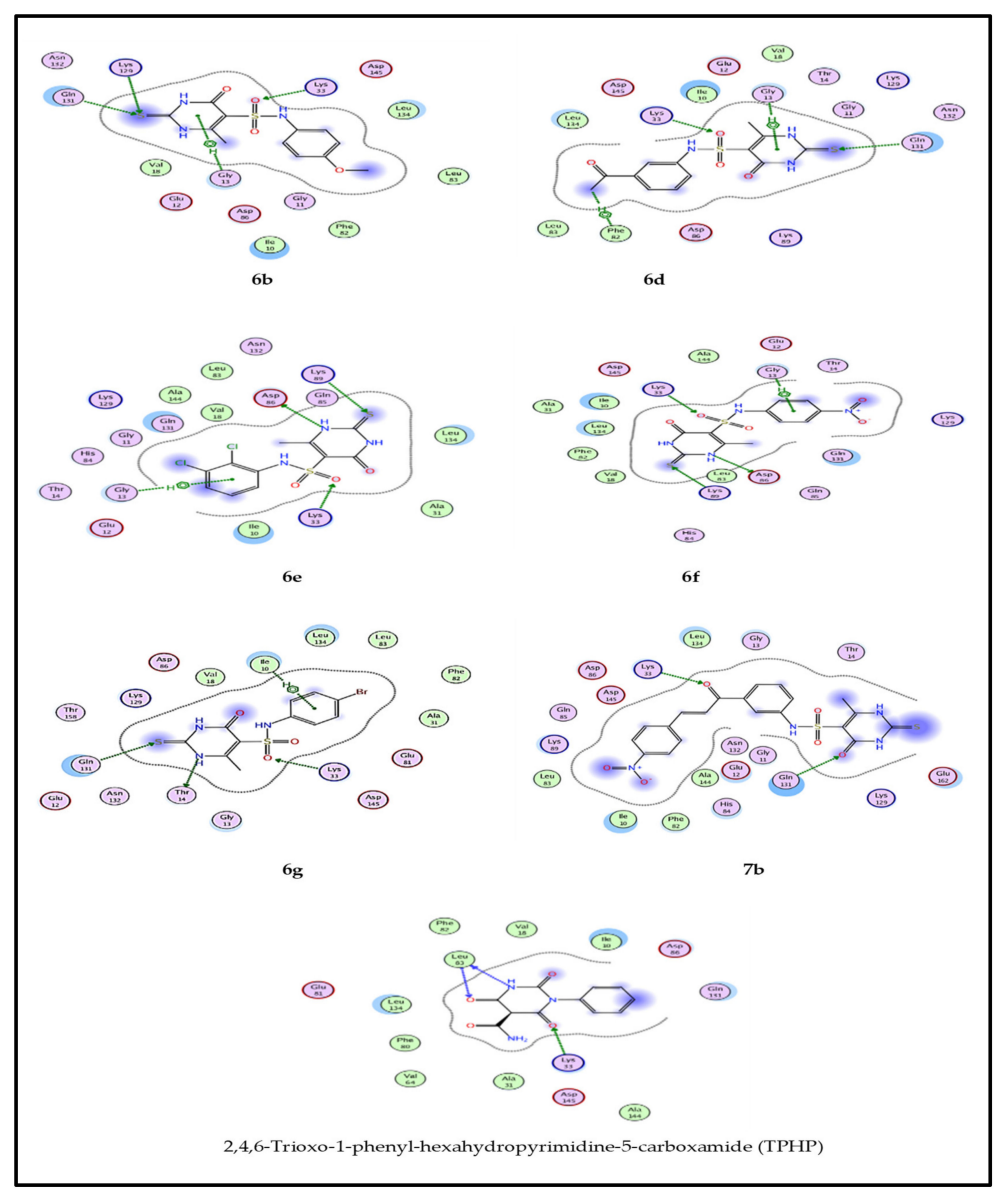
2D binding mode of compounds **6b, 6d–g, 7b** and **TPHP** with CDK2 ATP binding site.

**Figure 8:**
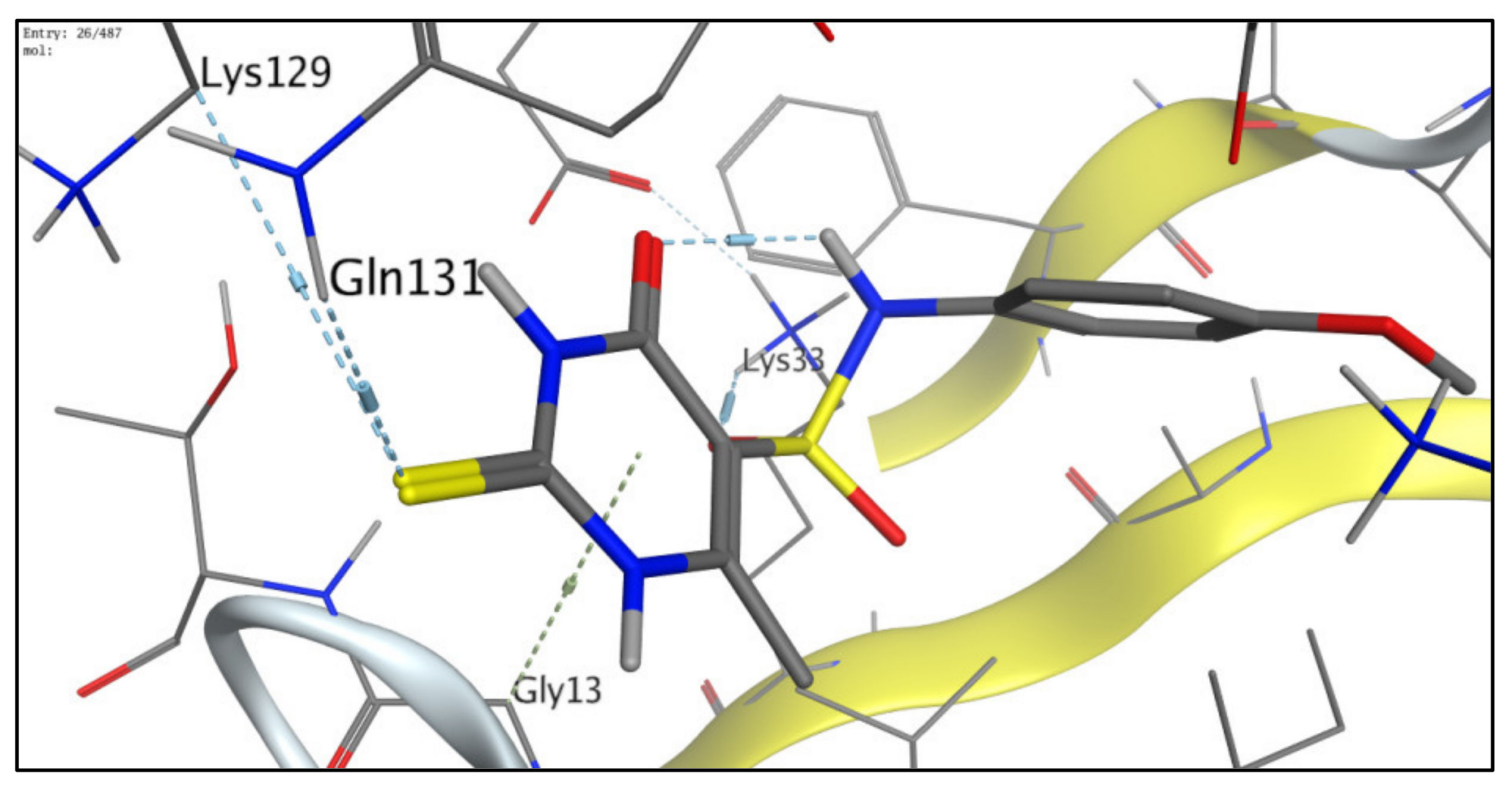
Molecular model for binding of **6e** forming three distinctive hydrogen bonds with Lys33, Lys129, and Gln131, in addition to arene-H binding with Gly13.

From the obtained results, compounds **6b**, **6d–g**, and **7b**, showed higher anti-cancer and in vitro CDK2 inhibition activities, and also the best docking results, as revealed in Figure 7. Interpretation of the docking results indicated that: compounds **6b**, **6d**, **6e**, **6f**, and **6g** bind to four residues, while TPHP binds with only two residues. Likewise, compound **7b** binds only with two residues, which explains its moderate activity against only one cell line. Compound **6e**, the most promising anticancer agent, exerted one hydrophobic bond with Gly 13 (2.89A^o^), along with three hydrogen bonds with Lys33 (2.98A^o^), Lys89 (4.29A^o^), and Asp86 (3.17A^o^), compared to three hydrogen bonds Lys33 (2.89A^o^), Leu83 (NH-3.56A^o^), and Leu83 (C=O. 3.21) for TPHP. The docking score (−5.48 kcal/mol) and RMSD (1.05) for compound **6e** indicated better binding affinity over TPHP, which showed docking score (−4.52 kcal/mol) and RMSD (1.86). The docking study explained medium to high activity of compound **6e** against four cell lines over the remain active compounds, as revealed in Figure 7 and Figure 8 and Supplementary Materials.

## 3. Materials and Methods

### 3.1. Chemical Experimental

All chemicals were purchased as reagent grade from Merck (Darmstadt, Germany). All melting points were uncorrected and measured using Electro-thermal IA 9100 apparatus (Shimadzu, Japan); IR spectra were recorded as potassium bromide pellets on a Perkin-Elmer 1650 spectrophotometer (CT, USA), Faculty of Science, Cairo University, Cairo, Egypt. A Varian Mercury spectrometer (Varian UK) was used to determine the ^1^H-NMR (300 MHz) and ^13^C-NMR (75 MHz) spectra and chemical shifts were expressed as ppm against TMS as internal reference (The Main Chemical warfare Laboratories, Almaza, Cairo, Egypt). Mass spectra were recorded on 70 eV EI Ms-QP 1000 EX (Shimadzu, Japan), Faculty of Science, Cairo University, Cairo, Egypt. Microanalyses were performed using Vario, Elmentar apparatus (Shimadzu, Japan), Organic Microanalysis Unit, Faculty of Science, Cairo University, Cairo, Egypt. The progress of the reactions was monitored by TLC sheets pre-coated with UV fluorescent silica gel (Merck 60 F254, Germany) using chloroform/methanol (3:1) and spots were visualized using UV-light and iodine vapor, TLC sheets were used also for testing compounds purity after recrystallization. All new compounds yielded spectral data consistent with the proposed structure and microanalysis within ±0.4% of the theoretical values. All compounds were newly prepared except for compound **2**, which was prepared according to previously reported method [61].

#### 3.1.1. Synthesis of 6-Methyl-4-Oxo-2-Thioxo-1,2,3,4-Tetrahydropyrimidine-5-Sulfonyl Chloride (**2**)

A mixture of 6-methyl-2-thiouracil (12.5 g, 0.0055 mol) and chlorosulphonic acid (51 mL, 0.055 mol) was refluxed at 120 °C for 8 h. The reaction mixture was cooled and poured on ice. The precipitate was filtered and dried under vacuum and recrystallized from DMF/water to give compound 2 which was then used as raw material for the next step.

Yield: 70%; m.p.: 324–326 °C; IR (KBr) υ (cm^−1^): 3320–3266 (NH), 2976 (aliphatic CH), 1685 (C=O), 1323,1145 (SO_2_); ^1^H-NMR (DMSO-d_6_, 300 MHz) δ (ppm): 2.32 (s, 3H, CH_3_), 10.89, 12.34 (s, 2H, 2NH, D_2_O exchangeable); ^13^C-NMR (DMSO, 75 MHz) δ (ppm): 18.49 (CH_3_), 99.9, 104.11(SP^2^ carbon atoms), 161.42 (C=O), 176.27 (C=S); MS (EI) *m/z*: 240.68 (M^+^, ^35^Cl, 23.56%), 242.68 (M^+^+2, ^37^Cl, 7.84%); Anal. Calcd. for C_5_H_5_ClN_2_O_3_S_2_ (240.69): C, 25.00; H, 2.08; N, 11.67%. Found: C, 24.87; H, 2.18; N, 11.68%. 

#### 3.1.2. General Synthesis of Sulfonylpyrimidine-4-One Derivatives (**3**–**5**)

A mixture of pyrimidine-5-sulfonyl chloride **2** (0.001 mol) and an equimolar amount of barbituric acid, dimedone, or 1-indanone in 25 mL sodium ethoxide/ethanol was refluxed with stirring for 8 h, cooled, poured on acidified ice till neutralization then the produced solid was filtered off, dried, and recrystallized from DMF/water.

#### 3.1.3. 5-[(6-Methyl-4-Oxo-2-Thioxo-1,2,3,4-Tetrahydropyrimidin-5-Yl)sulfonyl]pyrimidine-2,4,6(1H,3H,5H)-Trione (**3**)

Yield: 77%; m.p.: 344–346 °C; IR (KBr) υ (cm^−1^): 3369–3180 (NH), 2973 (aliphatic CH), 1689, 1678, 1667, 1655 (4C=O), 1332,1141 (SO_2_); ^1^H-NMR (DMSO-d_6_, 300 MHz) δ (ppm): 2.28 (s, 3H, CH_3_), 5.66 (s, 1H, CH), 9.84,10.85, 11.48, 12.21 (s, 4NH, D_2_O exchangeable); ^13^C-NMR (DMSO, 75 MHz) δ (ppm): 18.49 (CH_3_), 81.65 (barbituric acid carbon), 104.11, 153.12 (thiouracil SP^2^ carbons), 153.54, 161.42, 176.28 (4C=O), 176.50 (C=S); MS (EI) *m/z*: 332.5 (M^+^, 18.11%); Anal. Calcd. for C_9_H_8_ N_4_O_6_S_2_ (332.31): C, 32.53; H, 2.41; N, 16.87%. Found: C, 32.65; H, 2.35; N, 16.76%.

#### 3.1.4. 5,5-Dimethyl-2-[(6-Methyl-4-Oxo-2-Thioxo-1,2,3,4-Tetrahydropyrimidin-5-Yl)sulfonyl]cyclohexane-1,3-Dione (**4**)

Yield: 73%; m.p.: 340–342 °C; IR (KBr) υ (cm^−1^): 3377–3184 (NH), 2989 (aliphatic CH), 1723, 1713, 1665 (3C=O), 1331,1140 (SO_2_); ^1^H-NMR (DMSO-d_6_, 300 MHz) δ (ppm): 0.94 (s, 6H, 2CH_3_ dimedone), 2.04 (s, 3H, CH_3_), 2.38 (s, 4H, 2CH_2_), 5.82 (s, 1H, SO_2_-CH), 11.10, 12.21 (s, 2NH, D_2_O exchangeable); ^13^C-NMR (DMSO, 75 MHz) δ (ppm): 18.49 (CH_3_), 27.83 (2CH_3_ dimedone), 31.85, 46.09, 99.13 (dimedone carbons), 104.11, 153.53 (thiouracil SP^2^ carbons), 176.28 (C=S), 161.42, 190.49 (3C=O); MS (EI) *m/z*: 344.51 (M^+^, 15.12%); Anal. Calcd. for C_13_H_16_N_2_O_5_S_2_ (344.41): C, 45.35; H, 4.65; N, 8.14%. Found: C, 45.44; H, 4.76; N, 8.23%.

#### 3.1.5. 6-Methyl-5-(1-Oxo-2,3-Dihydro-1H-Inden-2-Ylsulfonyl)-2-Thioxo-2,3-Dihydropyrimidin-4(1H)-One (**5**)

Yield: 69%; m.p.: 348–350 °C; IR (KBr) υ (cm^−1^): 3382-3194 (NH), 2978 (aliphatic CH), 1716,1665 (2C=O), 1335,1142 (SO_2_); ^1^H-NMR (DMSO-d_6_, 300 MHz) δ (ppm): 2.07 (s, 3H, CH_3_), 2.61 (d, 2H, *J =* 7.4 Hz, CH2), 3.07 (t, 1H, *J =* 7.4 Hz, CH), 7.36–7.72 (m, 4H, Ar-H), 10.84, 12.29 (s, 2NH, D_2_O exchangeable); ^13^C-NMR (DMSO, 75 MHz) δ (ppm): 25.85 (CH_3_), 73.8, 84.5 (indene carbon), 104.11, 110.12, 129.2, 133.8, 153.7, 160.31 (SP^2^ carbon atoms), 162.4, 178.73 (2C=O), 179.8 (C=S); MS (EI) *m/z*: 336.39 (M^+^, 12.86%); Anal. Calcd. for C_14_H_12_ N_2_O_4_S_2_ (336.38): C, 50.00; H, 3.57; N, 8.33%. Found: C, 50.11; H, 3.73; N, 8.28%.

#### 3.1.6. General Synthesis of N-Substituted Pyrimidine-5-Sulfonamides (**6**)

A mixture of pyrimidine-5-sulfonyl chloride **2** (1.13 g, 0.005 mol), the suitable amine (0.005 mol), and pyridine (0.4 mL, 0.005 mol) was refluxed in 25 mL absolute ethanol for 16–20 h, then cooled, filtered off, dried, and recrystallized from DMF/water.

#### 3.1.7. 6-Methyl-4-Oxo-N-Phenyl-2-Thioxo-1,2,3,4-Tetrahydropyrimidine-5-Sulfonamide (**6a**)

Yield: 67%; m.p.: 316–318 °C; IR (KBr) υ (cm^−1^): 3325–3281 (NH), 3176 (aromatic CH), 2978 (aliphatic CH), 1681 (C=O), 1327,1143 (SO_2_); ^1^H-NMR (DMSO-d_6_, 300 MHz) δ (ppm): 2.11 (s, 3H, CH_3_), 7.12-7.52 (m, 5H, Ar-H), 5.54, 10.88, 12.34 (s, 3NH, D_2_O exchangeable); ^13^C-NMR (DMSO, 75 MHz) δ (ppm): 18.71 (CH_3_), 99.13, 104.11, 115.13, 130.43, 153.53, 161.42 (SP^2^ carbon atoms), 172.3 (C=O), 176.27 (C=S); MS (EI) *m/z*: 297.30 (M^+^, 18.43%); Anal. Calcd. for C_11_H_11_N_3_O_3_S_2_ (297.35): C, 44.44; H, 3.70; N, 14.14%. Found: C, 44.37; H, 3.78; N, 14.28%. 

#### 3.1.8. N-(4-Methoxyphenyl)-6-Methyl-4-Oxo-2-Thioxo-1,2,3,4-Tetrahydropyrimidine-5-Sulfon-Amide (**6b**)

Yield: 71%; m.p.: 320–322 °C; IR (KBr) υ (cm^−1^): 3333–3219 (NH), 3178 (aromatic CH), 2988 (aliphatic CH), 1675 (C=O), 1325,1141 (SO_2_); ^1^H-NMR (DMSO-d_6_, 300 MHz) δ (ppm): 2.20 (s, 3H, CH_3_), 5.29 (s, 3H, OCH_3_), 6.63 (d, 2H, *J =* 8.6 Hz, Ar-H), 7.26 (d, 2H, *J =* 8.6 Hz, Ar-H), 10.70, 10.83, 12.21 (s, 3NH, D_2_O exchangeable); ^13^C-NMR (DMSO, 75 MHz) δ (ppm): 18.6 (CH_3_), 45.89 (OCH_3_), 98.54, 105.1, 124.51, 130.12, 153.41, 160.0 (SP^2^ carbon atoms), 163.8 (C=O), 175.22 (C=S); MS (EI) *m/z*: 327.4 (M^+^, 21.76%); Anal. Calcd. for C_12_H_13_N_3_O_4_S_2_ (327.37): C, 44.04; H, 3.98; N, 12.84%. Found: C, 44.07; H, 3.91; N, 12.78%.

#### 3.1.9. 6-Methyl-4-Oxo-2-Thioxo-N-(P-Tolyl)-1,2,3,4-Tetrahydropyrimidine-5-Sulfonamide (**6c**)

Yield: 67%; m.p.: 313–315 °C; IR (KBr) υ (cm^−1^): 3336-3226 (NH), 3176 (aromatic CH), 2976 (aliphatic CH), 1687 (C=O), 1321,1147 (SO_2_); ^1^H-NMR (DMSO-d_6_, 300 MHz) δ (ppm): 2.03 (s, 3H, CH_3_), 2.21 (s, 3H, CH_3_), ), 6.94 (d, 2H, *J =* 8.4 Hz, Ar-H), 7.02 (d, 2H, *J =* 8.4 Hz, Ar-H), 8.77, 11.31, 12.26 (s, 3NH, D_2_O exchangeable); ^13^C-NMR (DMSO, 75 MHz) δ (ppm): 18.71 (CH_3_), 20.33 (CH_3_), 98.2, 103.39, 104.11, 119.1, 129.82, 153.53, 161.42(SP^2^ carbon atoms), 163.35 (C=O), 176.27 (C=S); MS (EI) *m*/*z*: 311.47 (M^+^, 17.98%); Anal. Calcd. for C_12_H_13_N_3_O_3_S_2_ (311.38): C, 46.30; H, 4.18; N, 13.50%. Found: C, 46.08; H, 4.39; N, 13.48%.

#### 3.1.10. N-(3-Acetylphenyl)-6-Methyl-4-Oxo-2-Thioxo-1,2,3,4-Tetrahydropyrimidine-5-Sulfonamide (**6d**)

Yield: 77%; m.p.: 318–320 °C; IR (KBr) υ (cm^−1^): 3354–3257 (NH), 3185 (aromatic CH), 2965 (aliphatic CH), 1700, 1676 (2 C=O), 1320,1140 (SO_2_); ^1^H-NMR (DMSO-d_6_, 300 MHz) δ (ppm): 2.04 (s, 3H, CH_3_), 2.30 (s, 3H, CH_3_-C=O), 7.35-7.382 (m, 4H, Ar-H), 9.97, 10.83, 12.27 (s, 3NH, D_2_O exchangeable); ^13^C-NMR (DMSO, 75 MHz) δ (ppm): 19.3 (CH_3_), 45.7 (C=OCH_3_*), 98.7, 113.21, 124.51, 130.88, 153.66, 160.34 (SP^2^ carbon atoms), 163.7, 168.9 (2C=O), 173.52 (C=S); MS (EI) *m/z*: 339.4 (M^+^, 25.98%); Anal. Calcd. for C_13_H_13_N_3_O_4_S_2_ (339.39): C, 46.02; H, 3.83; N, 12.39%. Found: C, 46.06; H, 3.69; N, 12.41%.

#### 3.1.11. N-(2,3-Dichlorophenyl)-6-Methyl-4-Oxo-2-Thioxo-1,2,3,4-Tetrahydropyrimidine-5-Sulfonamide (**6e**)

Yield: 73%; m.p.: 326–328 °C; IR (KBr) υ (cm^−1^): 3357-3294 (NH), 3187 (aromatic CH), 2985 (aliphatic CH), 1684 (C=O), 1327,1143 (SO_2_); ^1^H-NMR (DMSO-d_6_, 300 MHz) δ (ppm): 2.20 (s, 3H, CH_3_), 7.49-7.93 (m, 3H, Ar-H), 9.86, 10.71, 12.22 (s, 3NH, D_2_O exchangeable); ^13^C-NMR (DMSO, 75 MHz) δ (ppm): 17.3 (CH_3_), 99.4, 110.91, 125.7, 129.6, 132.2, 133.63, 152.09, 163.2 (SP^2^ carbon atoms), 164.3 (C=O), 174.8 (C=S); MS (EI) *m/z*: 366.26 (M^+^, ^35^Cl, 47.93%), 368.24 (M+2, ^37^Cl, 31.29%), 370.28 (M^+^+4, ^37^Cl, 5.64%); Anal. Calcd. for C_11_H_9_Cl_2_N_3_O_3_S_2_ (366.24): C, 36.07; H, 2.46; N, 11.48%. Found: C, 36.16; H, 2.59; N, 11.34%.

#### 3.1.12. 6-Methyl-N-(4-Nitrophenyl)-4-Oxo-2-Thioxo-1,2,3,4-Tetrahydropyrimidine-5-Sulfonamide (**6f**)

Yield: 65%; m.p.: 321–323 °C; IR (KBr) υ (cm^−1^): 3365–3312 (NH), 3182 (aromatic CH), 2977 (aliphatic CH), 1674 (C=O), 1547, 1350 (NO_2_), 1326,1144 (SO_2_); ^1^H-NMR (DMSO-d_6_, 300 MHz) δ (ppm): 2.09 (s, 3H, CH_3_), ), 8.12 (d, 2H, *J =* 8.6 Hz, Ar-H), 8.26 (d, 2H, *J =* 8.6 Hz, Ar-H), 10.84, 11.46, 12.21 (s, 3NH, D_2_O exchangeable); ^13^C-NMR (DMSO, 75 MHz) δ (ppm): 18.4 (CH_3_), 97.3, 110.7, 122.19, 131.32, 153.4, 159.84 (SP^2^ carbon atoms), 163.8 (C=O), 175.22 (C=S); MS (EI) *m/z*: 342.37 (M^+^, 12.94%); Anal. Calcd. for C_11_H_10_N_4_O_5_S_2_ (342.35): C, 38.60; H, 2.92; N, 16.37%. Found: C, 38.65; H, 3.00; N, 16.43%. 

#### 3.1.13. N-(4-Bromophenyl)-6-Methyl-4-Oxo-2-Thioxo-1,2,3,4-Tetrahydropyrimidine-5-Sulfonamide (**6g**)

Yield: 68%; m.p.: 333–335 °C; IR (KBr) υ (cm^−1^): 3377-3319 (NH), 3172 (aromatic CH), 2955 (aliphatic CH), 1670 (C=O), 1329,1143 (SO_2_); ^1^H-NMR (DMSO-d_6_, 300 MHz) δ (ppm): 2.27 (s, 3H, CH_3_), ), 7.21 (d, 2H, *J =* 7.8 Hz, Ar-H), 7.54 (d, 2H, *J =* 7.8 Hz, Ar-H), 10.21, 11.17, 12.31 (s, 3NH, D_2_O exchangeable); ^13^C-NMR (DMSO, 75 MHz) δ (ppm): 18.45 (CH_3_), 95.8, 111.3, 122.65, 131.27, 156.3, 162.3 (SP^2^ carbon atoms), 163.8 (C=O), 175.22 (C=S); MS (EI) *m/z:* 375.29 (M^+^, 17.89%), 377.23 (M^+^+2, 16.99%); Anal. Calcd. for C_11_H_10_BrN_3_O_3_S_2_ (376.25): C, 35.11; H, 2.66; N, 11.17%. Found: C, 35.22; H, 2.56; N, 11.23%. 

#### 3.1.14. General Synthesis of N-((Substituted Acryloyl)phenyl) Pyrimidine-5-Sulfonamides (**7**)

A mixture of N-(3-Acetylphenyl) pyrimidine-5-sulfonamide **6d** (1.13 g, 0.0005 mol) and suitable aromatic aldehyde (0.005 mol) was stirred in 25 mL 10% ethanolic sodium hydroxide solution at room temperature for 24 h., then under reflux for 2 h, cooled and poured on acidified ice water. The precipitate was filtered, dried under vacuum, and recrystallized from DMF/H_2_O.

#### 3.1.15. N-(3-(3-(4-Chlorophenyl)acryloyl)phenyl)-6-Methyl-4-Oxo-2-Thioxo-1,2,3,4-Tetrahydro Pyrimidine-5-Sulfonamide (**7a**)

Yield: 68%; m.p.: 315–317 °C; IR (KBr) υ (cm^−1^): 3365-3275 (NH), 3188 (aromatic CH), 2967 (aliphatic CH), 1715 (Ph-*C=O), 1676 (thiouracil C=O), 1330,1140 (SO_2_); ^1^H-NMR (DMSO-d_6_, 300 MHz) δ (ppm): 2.04 (s, 3H, CH_3_), 7.27 (m, 4H, Ar-H), 7.56 (d, 1H, *J =* 6.8 Hz, CH=CH), 7.62 (d,2H, *J =* 8.4 Hz, Ar-H), 7.81 (d,2H, *J =* 8.4 Hz, Ar-H), 7.93 (d, 1H, *J =* 6.8 Hz, CH=CH), 9.98, 10.78, 12.27 (s, 3NH, D_2_O exchangeable); ^13^C-NMR (DMSO, 75 MHz) δ (ppm): 18.54 (CH_3_), 99.03, 103.96, 104.41, 129.25, 129.71, 130.13, 131.57, 137.70, 138.24, 138.76, 151.95, 153.25, 153.56, 160.94 (SP^2^ carbon atoms), 161.06 (C=O), 166.86 (C=S), 176.26 (Ph-*C=O); MS (EI) *m/z*: 461 (M^+^, 26.82%), 463 (M^+^+2, 8.95%); Anal. Calcd. for C_20_H_16_Cl N_3_O_4_S_2_ (461.94): C, 52.06; H, 3.47; N, 9.11%. Found: C, 52.08; H, 3.32; N, 9.23%. 

#### 3.1.16. 6-Methyl-N-(3-(3-(4-Nitrophenyl)acryloyl)phenyl)-4-Oxo-2-Thioxo-1,2,3,4-Tetrahydropyrimidine-5-Sulfonamide (**7b**)

Yield: 64%; m.p.: 318-320^o^C; IR (KBr) υ (cm^−1^): 3364-3112 (NH), 3187 (aromatic CH), 2977 (aliphatic CH), 1717 (Ph-*C=O), 1670 (thiouracil C=O), 1553, 1348 (NO_2_), 1330,1140 (SO_2_); ^1^H-NMR (DMSO-d_6_, 300 MHz) δ (ppm): 2.24 (s, 3H, CH_3_), 7.12 (m, 4H, Ar-H), 7.54 (d, 2H, *Jn=* 8.4 Hz, Ar-H), 7.71 (d, 1H, *J =* 6.4 Hz, CH=CH), 7.81(d, 2H, *J =* 8.4 Hz, Ar-H), 8.12 (d, 1H, *J =* 6.4 Hz, CH=CH), 10.18, 11.22, 12.34 (s, 3NH, D_2_O exchangeable); ^13^C-NMR (DMSO, 75 MHz) δ (ppm): 17.26 (CH_3_), 97.04, 99.3, 104.43, 109.14, 113.6, 127.5, 129.33, 129.8, 130.12, 131.45, 137.42, 139.3, 153.41, 160.0 (SP^2^ carbon atoms), 163.7 (C=O), 175.34 (C=S); MS (EI) *m/z*: 472.52 (M^+^, 19.54%); Anal. Calcd. for C_20_H_16_ N_4_O_6_S_2_ (472.49): C, 50.85; H, 3.39; N, 11.86%. Found: C, 50.73; H, 3.38; N, 11.87%. 

#### 3.1.17. N-(3-(3-(4-Bromophenyl)acryloyl)phenyl)-6-Methyl-4-Oxo-2-Thioxo-1,2,3,4-Tetrahydro Pyrimidine-5-Sulfonamide (**7c**)

Yield: 72%; m.p.: 335–337 °C; IR (KBr) υ (cm^−1^): 3378-3299 (NH), 3167 (aromatic CH), 2975 (aliphatic CH), 1723 (Ph-*C=O), 1686 (thiouracil C=O), 1330,1140 (SO_2_); ^1^H-NMR (DMSO-d_6_, 300 MHz) δ (ppm): 2.02 (s, 3H, CH_3_), 7.26 (m, 4H, Ar-H), 7.45 (d, 1H, *J =* 7.4 Hz, CH=CH), 7.59 (d, 2H, *J =* 8.6 Hz, Ar-H), 7.76 (d, 2H, *J =* 8.6 Hz, Ar-H), 8.75 (d, 1H, *J =* 7.4 Hz, CH=CH), 9.86, 10.50, 12.21 (s, 3NH, D_2_O exchangeable); ^13^C-NMR (DMSO, 75 MHz) δ (ppm): 17.26 (CH_3_), 98.3, 99.7, 104.41, 109.12, 113.5, 127.3, 129.4, 129.91, 130.7, 131.2, 137.2, 139.16, 154.45, 160.7 (SP^2^ carbon atoms), 164.37 (C=O), 175.38 (C=S);MS (EI) *m*/*z*: 505.45 (M^+^, 30.01%), 507.42 (M^+^+2, 29.04%); Anal. Calcd. for C_20_H_16_Br N_3_O_4_S_2_ (506.39): C, 47.43; H, 3.16; N, 8.30%. Found: C, 47.50; H, 3.23; N, 8.32%. 

#### 3.1.18. General Synthesis of Tetrahydropyrimidine-5-Sulfonohydrazide Derivatives (**8**)

Equimolar amounts of pyrimidine-5-sulfonyl chloride **2** (0.001 mol) and thiosemicarbazide, phenylhydrazine, or 2,4-dinitrophenyl hydrazine were refluxed into pyridine (0.5 mL, 0.001 mol), with stirring for 10 h, then cooled and poured onto acidified ice. The resulting solid materials were filtered, dried under suction, and recrystallized from DMF/water.

#### 3.1.19. [(6-Methyl-4-Oxo-2-Thioxo-1,2,3,4-Tetrahydropyrimidin-5-Yl) Sulfonylamino] Thiourea (**8a**)

Yield: 71%; m.p.: 343–345 °C; IR (KBr) υ (cm^−1^): 3387–3186 (NH, NH_2_), 2976 (aliphatic CH), 1667 (C=O), 1330,1143 (SO_2_); ^1^H-NMR (DMSO-d_6_, 300 MHz) δ (ppm): 2.04 (s, 3H, CH_3_), 6.62, 7.08 (s, NHC=S, NH-SO_2_, D_2_O exchangeable),8.18 (s, NH_2_, D_2_O exchangeable), 10.83, 11.05 (s, 2NH thiouracil, D_2_O exchangeable); ^13^C-NMR (DMSO, 75 MHz) δ (ppm): 18.49 (CH_3_), 98.0,104.10 (Pyrimidine SP^2^ carbons), 161.46 (C=O), 153.57,176.26 (2C=S); MS (EI) *m/z*: 295.34 (M^+^, 24.86%); Anal. Calcd. for C_6_H_9_ N_5_O_3_S_3_ (295.36): C, 24.41; H, 3.05; N, 23.73%. Found: C, 24.23; H, 3.18; N, 23.67%.

#### 3.1.20. 6-Methyl-4-Oxo-N′-Phenyl-2-Thioxo-1,2,3,4-Tetrahydropyrimidine-5-Sulfonohydrazide (**8b**)

Yield: 58%; m.p.: 336–338 °C; IR (KBr) υ (cm^−1^): 3388–3260 (NH), 3078 (aromatic CH), 2986 (aliphatic CH), 1676 (C=O), 1330,1143 (SO_2_); ^1^H-NMR (DMSO-d_6_, 300 MHz) δ (ppm): 2.31 (s, 3H, CH_3_), 2.14, 5.65 (s, SO_2_-NH-NH, D_2_O exchangeable), 7.67–8.33 (m, 5H, Ar-H), 10.95, 12.13 (s, 2NH thiouracil, D_2_O exchangeable); ^13^C-NMR (DMSO, 75 MHz) δ (ppm): 18.50 (CH_3_), 113.22, 129.25, 129.81, 151.0 (Ar-C), 95.0, 153.56 (Pyrimidine SP^2^carbons), 161.44 (C=O), 176.26 (C=S); MS (EI) *m/z*: 312.34 (M^+^, 26.43%); Anal. Calcd. for C_11_H_12_N_4_O_3_S_2_ (312.37): C, 42.31; H, 3.85; N, 17.95%. Found: C, 42.20; H, 3.98; N, 17.87%.

#### 3.1.21. N′-(2,4-Dinitrophenyl)-6-Methyl-4-Oxo-2-Thioxo-1,2,3,4-Tetrahydropyrimidine-5-Sulfono Hydrazide (**8c**)

Yield: 60%; m.p.: 321-323^o^C; IR (KBr) υ (cm^−1^): 3374-3287 (NH), 3189 (aromatic CH), 2967 (aliphatic CH), 1677 (C=O), 1553,1356 (NO_2_), 1332,1140 (SO_2_); ^1^H-NMR (DMSO-d_6_, 300 MHz) δ (ppm): 2.12 (s, 3H, CH_3_), 2.23,5.31 (s, SO_2_-NH-NH, D_2_O exchangeable), 7.53–8.81 (m, 3H, Ar-H), 9.99, 12.31 (s, 2NH thiouracil, D_2_O exchangeable); ^13^C-NMR (DMSO, 75 MHz) δ (ppm): 18.63 (CH_3_), 99.12, 104.09, 115.98, 123.91, 127.99, 134.71, 149.64, 153.57 (SP^2^ carbons), 161.47(C=O), 176.25(C=S); MS (EI) *m/z*: 402.39 (M^+^, 22.67%); Anal. Calcd. for C_11_H_10_ N_6_O_7_S_2_ (402.36): C, 32.84; H, 2.49; N, 20.90%. Found: C, 32.93; H, 2.57; N, 20.69%.

#### 3.1.22. General Synthesis of N′-Substituted Benzoyl-Pyrimidine-5-Sulfonohydrazides (**9**)

Equimolar amounts of pyrimidine-5-sulfonyl chloride **2** (0.001 mol) and substituted benzohydrazides were refluxed in pyridine (0.5 mL, 0.001 mol) for 12 h, then cooled and poured onto acidified ice. The resulting solid materials were filtered, dried under suction, and recrystallized from DMF/water.

#### 3.1.23. N′-(4-Chlorobenzoyl)-6-Methyl-4-Oxo-2-Thioxo-1,2,3,4-Tetrahydropyrimidine-5-Sulfono- Hydrazide (**9a**)

Yield: 65%; m.p.: 327–329 °C; IR (KBr) υ (cm^−1^): 3378–3210 (NH), 3076 (aromatic CH), 2974 (aliphatic CH), 1680, 1667 (2C=O), 1331,1142 (SO_2_); ^1^H-NMR (DMSO-d_6_, 300 MHz) δ (ppm): 2.04 (s, 3H, CH_3_), 6.95 (d, 2H, *J =* 7.4 Hz, Ar-H), 7.21 (d, 2H, *J =* 7.4 Hz, Ar-H), 5.55, 8.186, 10.81, 12.21 (s, 4NH, D_2_O exchangeable); ^13^C-NMR (DMSO, 75 MHz) δ (ppm): 12.93 (CH_3_), 127.5, 128.8, 132.0, 132.1(Ar-C), 94.0, 161.0 (Pyrimidine SP^2^carbons), 163.8, 164.8 (2C=O), 175.5 (C=S); MS (EI) *m/z*: 374.96 (M^+^, 23.68%), 376.84 (M^+^+2, 7.9%); Anal. Calcd. for C_12_H_11_Cl N_4_O_4_S_2_ (374.82): C, 38.50; H, 2.94; N, 14.97%. Found: C, 38.39; H, 3.11; N, 14.90%.

#### 3.1.24. N′-(2-Chlorobenzoyl)-6-Methyl-4-Oxo-2-Thioxo-1,2,3,4-Tetrahydropyrimidine-5-Sulfono-Hydrazide (**9b**)

Yield: 62%; m.p.: 334–336 °C; IR (KBr) υ (cm^−1^): 3376–3246 (NH), 3177 (aromatic CH), 2977 (aliphatic CH), 1678,1669 (2C=O), 1337,1143 (SO_2_); ^1^H-NMR (DMSO-d_6_, 300 MHz) δ (ppm): 2.09 (s, 3H, CH_3_), 7.54–7.93 (m, 4H, Ar-H), 2.47, 6.24, 10.81, 12.09 (s, 4NH, D_2_O exchangeable); ^13^C-NMR (DMSO, 75 MHz) δ (ppm): 16.8 (CH_3_), 96.3, 111.3, 122.65, 125.3, 128.41, 131.3, 132.45, 160.7 (SP^2^carbons), 163.23, 170.28 (2C=O), 173.84 (C=S); MS (EI) *m/z:* 374.89 (M^+^, 16.17%), 376.81 (M^+^+2, 5.8%); Anal. Calcd. for C_12_H_11_ClN_4_O_4_S_2_ (374.82): C, 38.50; H, 2.94; N, 14.97%. Found: C, 38.59; H, 2.87; N, 15.05%.

#### 3.1.25. N′-(4-Bromobenzoyl)-6-Methyl-4-Oxo-2-Thioxo-1,2,3,4-Tetrahydropyrimidine-5-Sulfono-Hydrazide (**9c**)

Yield: 71%; m.p.: 339–341 °C; IR (KBr) υ (cm^−1^): 3377–3219 (NH), 3187 (aromatic CH), 2979 (aliphatic CH), 1670, 1658 (2C=O), 1350, 1142 (SO_2_); ^1^H-NMR (DMSO-d_6_, 300 MHz) δ (ppm): 2.31 (s, 3H, CH_3_), 7.54 (d, 2H, *J =* 6.8 Hz, Ar-H), 7.81 (d, 2H, *J =* 6.8 Hz, Ar-H), 2.12, 9.88, 10.62, 12.24 (s, 4NH, D_2_O exchangeable); ^13^C-NMR (DMSO, 75 MHz) δ (ppm): 18.49 (CH_3_), 104.11, 128.85, 129.13, 132.42, 136.33, 153.53 (SP^2^carbons), 161.42, 165.21 (2C=O), 176.28 (C=S); MS (EI) *m/z*: 418.31 (M^+^, 20.01%), 420.28 (M^+^+2, 19.32%); Anal. Calcd. for C_12_H_11_Br N_4_O_4_S_2_ (419.27): C, 34.37; H, 2.63; N, 13.37%. Found: C, 34.42; H, 2.57; N, 13.43%

### 3.2. Biological Evaluation

#### 3.2.1. Cells 

Four cancer cell lines were used in this study viz ovarian (A-2780), colon (HT-29), breast (MCF-7), and liver (HepG2) cancer cells. They were provided by the National Institute of Cancer, Cairo University (Cairo, Egypt). The selection of those cancer cells was encouraged by the stated anticancer activity of a number 5-Fluorouracil hybrids against the mentioned cancer cells [62,63,64] and based on the reported cytotoxic activity of a large range of bioactive cores containing sulfonamide moiety [65,66,67].

#### 3.2.2. In Vitro Cytotoxicity Assessment

Cells were seeded in 96 well plates at a density of 2 × 10^4^ and incubated to allow the attachment for 24 h. Then the cells were treated at different concentrations (2.5–10 μM) with 5-FU as a positive control or the synthesized compounds for 48h. 0.1% DMSO was used as a negative control. Afterward, cells were washed using PBS and the viability of cells was evaluated utilizing in vitro toxicology assay kit, MTT Based (Stock No. TOX-1, Sigma-Aldrich, Saint Louis, Missouri, USA), per the manufacturer’s instructions. The plates were read at 570 nm. IC_50_ was calculated using a sigmoidal dose-response curve (GraphPad Prism version 5 for Windows (GraphPad Inc., USA)). Data are presented as the mean of the three individual experiments. The degree of selectivity of the active target compounds relative to WI38 normal cell lines is expressed as previously described [68]. Selectivity index (SI) = IC_50_ of a pure compound in a normal cell line/IC_50_ of the same pure compound in a cancerous cell line.

#### 3.2.3. In Vitro Inhibitory Screening of Cyclin-Dependent Kinase2 (CDK2) 

To measure CDK2/CyclinA2 activity, the CDK2 Assay kit #79599 was used for screening and profiling applications, together with Kinase-Glo® MAX (Promega#V6071) as a detection reagent. The assay was done as formerly described, using Roscovitine as a reference standard [69]. Briefly, 25 μL of the master mixture containing 6 μL Kinase assay buffer + 1 μL ATP + 5 μL CDK substrate peptide + 13 μL distilled water were added to each well. Later, 5 µL of inhibitor buffer only or diluted compound were added to the blank or test inhibitor wells, respectively. Then, 20μL of Kinase assay buffer was added to blank wells. The reaction was started by the addition of 20 μL of CDK2/CyclinA2 enzyme to the wells of the test inhibitor. The final volume was 50 µL in all of the reactions. Incubation was at 30 °C for 45 min for all the enzymatic reactions. Finally, to each well, 50 μL of Kinase-Glo MAX reagent was added. The plate was incubated for 15 min at room temperature then, the luminescence signal was measured using a Bio Tek Synergy 2 microplate reader. Experiments were performed in triplicates.

#### 3.2.4. Apoptosis Assay of Compound **6e** Using the Annexin-V-FITC Apoptosis Detection Kit 

For the apoptosis assay, the BioVision annexin V-FITC apoptosis detection kit (Cat No.: K101; BioVision, Inc., Milpitas, CA, USA) was used, per the manufacturer’s instructions [70]. Briefly, cells were treated with compound **6e** at its IC_50_ concentration or 0.1% DMSO as a control for 48 h. The cells were resuspended in 500 μL of Annexin V–binding buffer, followed by the addition of 5μL of Annexin V–FITC, then incubated in the dark for 10 min at room temperature. Analysis was performed using a FACS Calibur flow cytometer (BD Biosciences, San Jose, CA, USA), using CELLQUEST software (Becton Dickinson Immunocytometry Systems, San Jose, CA, USA).

#### 3.2.5. Cell Cycle Analysis of Compound **6e**

To evaluate cancer cells distribution through different cell cycle stages, the DNA content of propidium iodide-stained nuclei was assessed by flow cytometry as depicted earlier [71]. The cells were treated with IC_50_ of compound 6e or 0.1% DMSO as a control for 48h. Then, the cells were washed twice with ice-cold phosphate-buffered saline (PBS) and gathered by centrifugation. The cell pellets were mixed with 75% ethanol (−20 °C). Afterward, cells were stained with the propidium iodide flow cytometry kit (ab139418, Abcam, Cambridge, MA, USA), according to the manufacturer’s instructions. Cell cycle distribution was established by FACS Calibur flow cytometer (BD Biosciences, San Jose, CA, USA). Cell cycle distribution was measured using CELLQUEST software (Becton Dickinson Immunocytometry Systems, San Jose, CA, USA).

#### 3.2.6. Western Blot Analysis for p21 and p27

MCF-7 cells were seeded, cultured, and subjected to IC_50_ of compound 6e (1.67 μM) for 48 h. Whole-cell protein lysates were prepared according to a standard protocol using RIPA buffer (Cell Signaling, Danvers, MA, USA). Total protein concentrations were determined in the supernatant using the Bradford method. Equal amounts (20 µg) of protein samples were mixed and boiled with SDS Loading buffer for 10 min, allowed to cool on ice and then loaded into SDS-polyacrylamide gel and separated by Cleaver electrophoresis unit (Cleaver, UK), transferred onto polyvinylidene fluoride (PVDF) membranes (BioRad, USA) for 30 min using a Semi-dry Electroblotter (Biorad, USA) at 2.5 A and 25 V for 30 min. Membranes were blocked with 5% non-fat dry milk in TBS-T and incubated overnight with the primary antibody: anti-p21, anti-p27 antibody, or anti-β-actin antibody (1:1000; Cell Signaling Technology, Inc., Danvers, MA, USA), then incubated with secondary HRP-linked antibody (1:5000). Development was done using Pierce ECL2 chemiluminescent and chemifluorescent substrate (Perkin Elmer, Waltham, MA, USA). The chemiluminescent signals were captured using a CCD camera-based imager (Chemi Doc imager, Biorad, USA), and the bands’ intensities were then measured relative to actin by ImageLab (Biorad). 

#### 3.2.7. Data Presentation and Statistical Analysis

Significant differences between groups were tested by using GraphPad InStat software version 3.05 (GraphPad Inc., La Jolla, CA, USA). Appropriate graphs were plotted when needed using GraphPad Prism version 5 for Windows (GraphPad Inc., USA). *p*-value < 0.05 was considered statistically significant.

### 3.3. Molecular Docking 

All compounds were constructed using MOE 2014.09 and filed in a molecular database file [72]. The crystal structure of CDK2A was attained from the protein data bank (PDBID: 5ANJ) [73]. Protein was energy minimized and 3D protonated via the structure preparation module of MOE. The co-crystallized bound compound and water molecules were removed from the crystal structure. The site of docking was recognized and the database containing all the tested compounds has been established using rigid receptor as a docking protocol and triangle matcher as a placement method. Two rescoring functions were selected, London dG and GBVI/WSA dG. The force field was used as a refinement. Free binding energy (kcal/mol) was calculated and only the best-scored pose was selected for each compound.

## 4. Conclusions

New synthetic analogs of 2-thiouracil-5-sulphonamides were synthesized, screened for their anti-cancer effect against a panel of four cancer cell lines and in-vitro CDK2 inhibition activity. Molecular docking was conceded in a way trying to declare their potential mode of action. 

Sulphonamides **6b**, **6e**, and **6g** (containing 4-methoxyphenyl, 2,3-dichlorophenyl, 4-bromophenyl radicals, respectively) were the most potent with a wide range of cytotoxic activities against all cell lines and showed promising CDK2 inhibition activities. The dichlorophenol derivative **6e** exhibited superior antitumor activity as well as CDK2 inhibition. Thus, it was selected for inspection of the potential mechanisms of its cytotoxic activity. The results indicated that **6e** was able to cause cell cycle arrest at G1/S phase in ovarian cancer cells (A2780), S phase in colon cancer (HT-29), and breast cancer (MCF-7) cells, together with G2/M in liver cancer cells (HepG2), and displayed good apoptotic activity as evidenced by Annexin V-FITC apoptosis assay. Moreover, **6e** was able to cause cycle arrest indirectly through enhanced expression of cell cycle inhibitors p21 and p27. Hence, it could be considered as a good lead candidate for additional optimization of new potent anticancer agents.

## Figures and Tables

**Table 1:** Cytotoxic activity of the newly synthesized compounds against human cancer cells.

Compound	IC_50_ (μM)			
	A-2780	HT-29	MCF-7	HepG2
**2**	21.35 ± 2.51	18.03 ± 1.04	16.53 ± 1.40	16.99 ± 0.93
**3**	NA	13.00 ± 2.00	14.92 ± 2.89	NA
**4**	NA	14.20 ± 2.85	10.83 ± 1.60	NA
**5**	NA	11.71 ± 1.96	17.33 ± 2.35	NA
**6a**	17.72 ± 2.18	11.33 ± 2.08	16.75 ± 2.11	15.64 ± 1.52
**6b**	2.56 ± 0.45	5.10 ± 1.32	2.29 ± 0.63	2.23 ± 0.55
**6c**	14.03 ± 1.05	5.46 ± 1.66	6.36 ± 1.20	6.71 ± 0.99
**6d**	11.67 ± 1.99	6.13 ± 0.53	2.30 ± 0.62	5.24 ± 1.05
**6e**	2.52 ± 0.47	1.75 ± 0.40	1.67 ± 0.31	2.02 ± 0.85
**6f**	10.16 ± 0.97	6.40 ± 1.24	2.34 ± 0.61	6.10 ± 0.48
**6g**	1.62 ± 0.35	5.05 ± 0.83	7.31 ± 1.21	2.47 ± 0.51
**7a**	12.47 ± 2.10	4.20 ± 0.88	6.90 ± 1.33	10.91 ± 1.72
**7b**	11.07 ± 1.41	2.84 ± 0.78	6.11 ± 0.50	12.42 ± 1.82
**7c**	6.10 ± 0.54	8.14 ± 1.95	11.65 ± 2.07	8.25 ± 0.68
**8a**	16.83 ± 1.82	8.02 ± 1.99	13.31 ± 3.00	17.98 ± 1.95
**8b**	16.39 ± 1.55	8.16 ± 2.07	9.89 ± 1.80	13.06 ± 2.04
**8c**	8.57 ± 1.79	10.11 ± 1.54	5.54 ± 1.58	14.76 ± 1.45
**9a**	11.87 ± 3.47	6.64 ± 1.19	10.78 ± 1.53	10.99 ± 1.23
**9b**	7.95 ± 1.75	10.62 ± 1.25	13.58 ± 2.65	13.29 ± 1.50
**9c**	8.16 ± 2.07	6.99 ± 1.35	11.88 ± 2.00	9.44 ± 1.03
**5-FU**	3.92 ± 1.00	4.69 ± 1.13	5.15 ± 1.14	38.44 ± 2.40

IC_50_ values are the mean of three individual experiments ± SD; NA: not active.

**Table 2:** Cytotoxic activity of compounds **6b–g** and **7a–c** against WI38 normal cells and their SI.

Compound	IC_50_ (μM) WI38	SI *			
		A-2780	HT-29	MCF-7	HepG2
**6b**	71.66 ± 2.00	28.00	14.05	31.29	32.13
**6c**	84.43 ± 1.70	6.02	15.46	13.27	12.58
**6d**	74.38 ± 4.74	6.37	12.13	32.33	11.08
**6e**	60.21 ± 1.77	23.91	34.40	36.05	29.80
**6f**	65.68 ± 2.28	6.46	10.26	28.06	10.76
**6g**	70.61 ± 2.14	43.58	13.98	9.65	38.58
**7a**	83.08 ± 2.00	6.66	19.78	12.04	7.61
**7b**	79.92 ± 2.56	7.22	28.14	13.08	6.43
**7c**	62.72 ± 1.93	10.28	7.71	5.38	7.60

* SI: Selectivity index.

**Table 3:** CDK2 Protein Kinase Inhibitory Activity of compounds **6b,d–g** and **7a–c**.

Compound	CDK2 Protein Kinase (IC_50_ in µM)
**6b**	0.447 ± 0.031
**6d**	0.567 ± 0.021
**6f**	0.605± 0.023
**6g**	0.485 ± 0.033
**6e**	0.407 ± 0.021
**7a**	6.410 ± 0.377
**7b**	0.810 ± 0.026
**7c**	8.203 ± 0.486
**Roscovitine**	0.259 ± 0.014

IC_50_ values are the mean of three individual experiments ± SD.

**Table 4:** Distribution of apoptotic cells in the Annexin V-FITC/PI dual staining assay in A-2780, HT-29, MCF-7, and HepG2 cells after treatment with **6e**.

Treatment	% Early Apoptosis	% Late Apoptosis	% Necrosis	% Total
**6e/A-2780**	2.38	22.02	9.41	33.81
**Control/A-2780**	0.54	0.19	0.75	1.48
**6e/HT-29**	1.61	26.24	14.51	42.36
**Control/HT-29**	0.69	0.25	1.47	2.41
**6e/MCF-7**	3.82	23.71	11.75	39.28
**Control/MCF-7**	0.46	0.08	1.18	1.72
**6e/HepG2**	0.84	18.97	8.81	28.62
**Control/HepG2**	0.71	0.33	1.15	2.19

**Table 5:** Results of molecular adhesion of the most active compounds (**6b, 6d–g**, and **7b**) versus CDK-2A (PDB ID: 5ANJ).

Compound	*(S)* *Kcal/mol*	*RMSD*	*E Score 1* *Kcal/mol*	*E Score 2* *Kcal/mol*	*Binding Interaction* *(Receptor-Ligand)*
**6b**	*−5.82*	*1.62*	*−10.30*	*−5.82*	*Lys33-(S=O) (H-b, 2.87 A^o^)* *Gln131-(C=S), (H-b, 3.62 A^o^)* *Gly13-(pyrimidine) (H-pi, 4.22 A^o^)* *Lys129 (C=S) (H-b, 4.48 A^o^)*
**6d**	*−5.29*	*1.71*	*−10.27*	*−5.29*	*Lys33-(S=O) (H-b, 2.94 A^o^)* *Phe82-(CH3-C=O) (Pi-H, 4.73 A^o^)* *Gln131-(C=S) (H-b, 3.87 A^o^)* *Gly13-(pyrimidine) (H-Pi, 3.89 A^o^)*
**6e**	*−5.48*	*1.05*	*−9.97*	*−5.48*	*Lys33-(S=O) (H-b, 2.98 A^o^)* *Gly 13-(Ph) (H-Pi, 4.09 A^o^)* *Lys89-(C=S) (H-b, 4.29 A^o^)* *Asp86-(NH)( H-b, 3.17 A^o^)*
**6f**	*−5.72*	*1.05*	*−10.24*	*−5.72*	*Lys33-(S=O) (H-b, 3.10 A^o^)* *Gly 13-(Ph) (H-Pi, 3.99 A^o^)* *Lys89-(C=S) (H-b, 3.89 A^o^)* *Asp86-(NH)( H-b, 3.14 A^o^)*
**6g**	*−6.08*	*1.26*	*−9.83*	*−6.08*	*Lys33-(S=O) (H-b, 3.29 A^o^)* *ILe10-(Ph) (H-Pi, 3.80 A^o^)* *Gln131-(C=S) (H-b, 4.05 A^o^)* *Thr14-(NH)( H-b, 3.21 A^o^)*
**7b**	*−6.92*	*1.97*	*−12.46*	*−6.92*	*Lys33-(CH-C=O) (H-b, 2.98 A^o^)* *Gln131-(thiouracil C=O)* *(H-b, 3.35 A^o^)*
**TPHP**	*−4.52*	*1.86*	*−9.37*	*−4.52*	*Lys33-(C=O) (H-b, 2.89 A^o^)* *Leu83-(NH) (H-b, 3.56 A^o^)* *Leu83-(C=O) (H-b, 3.21 A^o^)*
TPHP: 2,4,6-Trioxo-1-phenyl-hexahydropyrimidine-5-carboxamide. E score 1 (London dG) Docking Score (S)					

## Data Availability

The data presented in this study are available in Supplementary Materials section.

## Supplementary Materials

The following are available online at https://www.mdpi.com/article/10.3390/ijms222111957/s1.

## References

[1] Bray F., Ferlay J., Soerjomataram I., Siegel R.L., Torre L.A., Jemal A. (2018). Global cancer statistics 2018: GLOBOCAN estimates of incidence and mortality worldwide for 36 cancers in 185 countries. CA Cancer J. Clin..

[2] Zheng L.W., Li Y., Ge D., Zhao B.X., Liu Y.R., Lv H.S., Ding J., Miao J.Y. (2010). Synthesis of novel oxime-containing pyrazole derivatives and discovery of regulators for apoptosis and autophagy in A549 lung cancer cells. Bioorg. Med. Chem. Lett..

[3] Fares M., Abou-Seri S.M., Abdel-Aziz H.A., Abbas S.E., Youssef M.M., Eladwy R.A. (2014). Synthesis and antitumor activity of pyrido [2,3-d]pyrimidine and pyrido[2,3-d] [1,2,4]triazolo[4,3-a]pyrimidine derivatives that induce apoptosis through G1 cell-cycle arrest. Eur. J. Med. Chem..

[4] Vekariya M.K., Vekariya R.H., Brahmkshatriya P.S., Shah N.K. (2018). Pyrimidine-based pyrazoles as cyclin-dependent kinase 2 inhibitors: Design, synthesis, and biological evaluation. Chem. Biol. Drug Des..

[5] Anscombe E., Meschini E., Mora-Vidal R., Martin M.P., Staunton D., Geitmann M., Danielson U.H., Stanley W.A., Wang L.Z., Reuillon T. (2015). Identification and Characterization of an Irreversible Inhibitor of CDK2. Chem. Biol..

[6] Baillache D.J., Unciti-Broceta A. (2020). Recent developments in anticancer kinase inhibitors based on the pyrazolo[3,4-d]pyrimidine scaffold. RSC Med. Chem..

[7] Whittaker S.R., Barlow C., Martin M.P., Mancusi C., Wagner S., Self A., Barrie E., Te Poele R., Sharp S., Brown N. (2018). Molecular profiling and combinatorial activity of CCT068127: A potent CDK2 and CDK9 inhibitor. Mol. Oncol..

[8] Bauer M.R., Joerger A.C., Fersht A.R. (2016). 2-Sulfonylpyrimidines: Mild alkylating agents with anticancer activity toward p53-compromised cells. Proc. Natl. Acad. Sci. USA.

[9] Longley D.B., Harkin D.P., Johnston P.G. (2003). 5-fluorouracil: Mechanisms of action and clinical strategies. Nat. Rev. Cancer.

[10] Kufe D.W., Major P.P., Egan E.M., Loh E. (1981). 5-Fluoro-2′-deoxyuridine incorporation in L1210 DNA. J. Biol. Chem..

[11] Lewin F., Skog S., Tribukait B., Rjngborg U. (1987). Effect of 5-fluorouracil on the cell growth and cell cycle kinetics of a mouse ascites tumor growing in vivo. Acta Oncol..

[12] Takeda H., Haisa M., Naomoto Y., Kawashima R., Satomoto K., Yamatuji T., Tanaka N. (1999). Effect of 5-fluorouracil on cell cycle regulatory proteins in human colon cancer cell line. Jap. J. Cancer Res..

[13] Gu J., Li Z., Zhou J., Sun Z., Bai C. (2019). Response prediction to oxaliplatin plus 5-fluorouracil chemotherapy in patients with colorectal cancer using a four-protein immunohistochemical model. Oncol. Lett..

[14] Zhang N., Yin Y., Xu S.J., Chen W.S. (2008). 5-Fluorouracil: Mechanisms of resistance and reversal strategies. Molecules.

[15] Yata V.K., Mahajan S., Thapa A., Ahmed S., Biswas A.D., Sanjeev A., Mattaparthi V.S.K. (2016). In silico methods reconfirm CDK2 as a potential molecular target of 5-fluorouracil. Indian J. Biochem. Biophys..

[16] Alsfouk A. (2021). Small molecule inhibitors of cyclin-dependent kinase 9 for cancer therapy. J. Enzym. Inhib. Med. Chem..

[17] Toogood P.L., Harvey P.J., Repine J.T., Sheehan D.J., VanderWel S.N., Zhou H., Keller P.R., McNamara D.J., Sherry D., Zhu T. (2005). Discovery of a potent and selective inhibitor of cyclin-dependent kinase 4/6. J. Med. Chem..

[18] VanderWel S.N., Harvey P.J., McNamara D.J., Repine J.T., Keller P.R., Quin J., Booth R.J., Elliott W.L., Dobrusin E.M., Fry D.W. (2005). Pyrido[2,3-d]pyrimidin-7-ones as specific inhibitors of cyclin-dependent kinase 4. J. Med. Chem..

[19] Boschi D., Tosco P., Chandra N., Chaurasia S., Fruttero R., Griffin R., Wang L.Z., Gasco A. (2013). 6-Cyclohexylmethoxy-5-(cyano-NNO-azoxy)pyrimidine-4-amine: A new scaffold endowed with potent CDK2 inhibitory activity. Eur. J. Med. Chem..

[20] Palmer B.D., Smaill J.B., Rewcastle G.W., Dobrusin E.M., Kraker A., Moore C.W., Steinkampf R.W., Denny W.A. (2005). Structure–activity relationships for 2-anilino-6-phenylpyrido[2, 3-d]pyrimidin-7(8H)-ones as inhibitors of the cellular checkpoint kinase Wee1. Bioorg. Med. Chem. Lett..

[21] Heidelberger C., Chaudhuri N.K., Danneberg P., Mooren D., Griesbach L., Duschinsky R., Schnitzer R.J., Pleven E., Scheiner J. (1957). Fluorinated pyrimidines, a new class of tumour-inhibitory compounds. Nature.

[22] DePinto W., Chu X.-J., Yin X., Smith M., Packman K., Goelzer P., Lovey A., Chen Y., Qian H., Hamid R. (2006). In vitro and in vivo activity of R547: A potent and selective cyclin-dependent kinase inhibitor currently in phase I clinical trials. Mol. Cancer Ther..

[23] Xia P., Liu Y., Chen J., Coates S., Liu D.X., Cheng Z. (2018). Inhibition of cyclin-dependent kinase 2 protects against doxorubicin-induced cardiomyocyte apoptosis and cardiomyopathy. J. Biol. Chem..

[24] Jorda R., Paruch K., Krystof V. (2012). Cyclin-dependent kinase inhibitors inspired by roscovitine: Purine bioisosteres. Curr. Pharm. Des..

[25] Sridhar J., Akula N., Pattabiraman N. (2006). Selectivity and potency of cyclin-dependent kinase inhibitors. AAPS J..

[26] Richardson C.M., Nunns C.L., Williamson D.S., Parratt M.J., Dokurno P., Howes R., Borgognoni J., Drysdale M.J., Finch H., Hubbard R.E. (2007). Discovery of a potent CDK2 inhibitor with a novel binding mode, using virtual screening and initial, structure-guided lead scoping. Bioorg. Med. Chem. Lett..

[27] Zhao J., Yin B., Sun H., Pang L., Chen J. (2020). Identifying hot spots of inhibitor-CDK2 bindings by computational alanine scanning. Chem. Phys. Lett..

[28] Hydbring P., Malumbres M., Sicinski P. (2016). Non-canonical functions of cell cycle cyclins and cyclin-dependent kinases. Nat. Rev. Mol. Cell Biol..

[29] Chen G., Wu D.I., Guo W., Cao Y., Huang D., Wang H., Wang T., Zhang X., Chen H., Yu H. (2020). Clinical and immunological features of severe and moderate coronavirus disease 2019. J. Clin. Investig..

[30] Ali G.M.E., Ibrahim D.A., Elmetwali A.M., Ismail N.S.M. (2019). Design, synthesis and biological evaluation of certain CDK2 inhibitors based on pyrazole and pyrazolo[1,5-a] pyrimidine scaffold with apoptotic activity. Bioorg. Chem..

[31] Law M.E., Corsino P.E., Narayan S., Law B.K. (2015). Cyclin-Dependent Kinase Inhibitors as Anticancer Therapeutics. Mol. Pharmacol..

[32] Malumbres M., Barbacid M. (2009). Cell cycle, CDKs and cancer: A changing paradigm. Nat. Rev. Cancer.

[33] Malumbres M. (2014). Cyclin-dependent kinases. Genome Biol..

[34] Otto T., Sicinski P. (2017). Cell cycle proteins as promising targets in cancer therapy. Nat. Rev. Cancer.

[35] Tutone M., Almerico A.M. (2017). Recent advances on CDK inhibitors: An insight by means of in silico methods. Eur. J. Med. Chem..

[36] Madia V.N., Nicolai A., Messore A., De Leo A., Ialongo D., Tudino V., Saccoliti F., De Vita D., Scipione L., Artico M. (2021). Design, Synthesis and Biological Evaluation of New Pyrimidine Derivatives as Anticancer Agents. Molecules.

[37] Jansa J., Jorda R., Skerlova J., Pachl P., Perina M., Reznickova E., Heger T., Gucky T., Rezacova P., Lycka A. (2021). Imidazo[1,2-c]pyrimidin-5(6H)-one inhibitors of CDK2: Synthesis, kinase inhibition and co-crystal structure. Eur. J. Med. Chem..

[38] Hassan S.M., al-Jaf A.N.A., Hussien Y.A., Awad S.M., Hadi N.R. (2020). The potential antiviral activity of a novel pyrimidine derivative against Herpes Simplex Virus type-1 (HSV-1). Rev. Pharm..

[39] Walker R.T., Jones A.S., Rahim S.G., Serafinowski P., De Clercq E. (1981). The synthesis and properties of some 5-substituted uracil derivatives. Nucleic Acids Symp. Ser..

[40] Cremlyn R.J., Frearson M.J., Graham S. (1995). The synthesis and chlorosulfonation of some diarylidene and heteroarylidene ketones with varying alicyclic ring size. Phosphorus Sulfur Silicon Relat. Elem..

[41] Sharma V., Chitranshi N., Agarwal A.K. (2014). Significance and biological importance of pyrimidine in the microbial world. Int. J. Med. Chem..

[42] Eweas A.F., Abdallah Q.M., Hassan E.S. (2014). Design, synthesis, molecular docking of new thiopyrimidine-5-carbonitrile derivatives and their cytotoxic activity against HepG2 cell line. J. Appl. Pharm. Sci..

[43] Wang Y.-G., Barnes E.C. (2018). O-Regioselective Synthesis with the Silver Salt Method. ACS Omega.

[44] Arias J.L. (2008). Novel strategies to improve the anticancer action of 5-fluorouracil by using drug delivery systems. Molecules.

[45] Gillis E.P., Eastman K.J., Hill M.D., Donnelly D.J., Meanwell N.A. (2015). Applications of fluorine in medicinal chemistry. J. Med. Chem..

[46] Maybaum J., Ullman B., Mandel H.G., Day J.L., Sadee W. (1980). Regulation of RNA-and DNA-directed actions of 5-fluoropyrimidines in mouse T-lymphoma (S-49) cells. Cancer Res..

[47] Hübbers A., Hennings J., Lambertz D., Haas U., Trautwein C., Nevzorova Y.A., Sonntag R., Liedtke C. (2020). Pharmacological Inhibition of Cyclin-Dependent Kinases Triggers Anti-Fibrotic Effects in Hepatic Stellate Cells In Vitro. Int. J. Mol. Sci..

[48] Schloer S., Pajonczyk D., Rescher U. (2018). Annexins in Translational Research: Hidden Treasures to Be Found. Int. J. Mol. Sci..

[49] Rieger A.M., Nelson K.L., Konowalchuk J.D., Barreda D.R. (2011). Modified annexin V/propidium iodide apoptosis assay for accurate assessment of cell death. J. Vis. Exp..

[50] Crowley L.C., Marfell B.J., Scott A.P., Waterhouse N.J. (2016). Quantitation of apoptosis and necrosis by annexin V binding, propidium iodide uptake, and flow cytometry. Cold Spring Harb. Protoc..

[51] Wlodkowic D., Telford W., Skommer J., Darzynkiewicz Z. (2011). Apoptosis and beyond: Cytometry in studies of programmed cell death. Methods Cell Biol..

[52] Visconti R., Della Monica R., Grieco D. (2016). Cell cycle checkpoint in cancer: A therapeutically targetable double-edged sword. J. Exp. Clin. Cancer Res..

[53] Gartel A.L., Radhakrishnan S.K. (2005). Lost in transcription: p21 repression, mechanisms, and consequences. Cancer Res..

[54] Chiarle R., Pagano M., Inghirami G. (2001). The cyclin dependent kinase inhibitor p27 and its prognostic role in breast cancer. Breast Cancer Res..

[55] Sluyser M. (2005). Application of Apoptosis to Cancer Treatment.

[56] He G., Kuang J., Huang Z., Koomen J., Kobayashi R., Khokhar A.R., Siddik Z.H. (2006). Upregulation of p27 and its inhibition of CDK2/cyclin E activity following DNA damage by a novel platinum agent are dependent on the expression of p21. Br. J. Cancer.

[57] Li Y., Gao W., Li F., Wang J., Zhang J., Yang Y., Zhang S., Yang L. (2013). An in silico exploration of the interaction mechanism of pyrazolo [1, 5-a] pyrimidine type CDK2 inhibitors. Mol. Biosyst..

[58] Chen H., Van Duyne R., Zhang N., Kashanchi F., Zeng C. (2009). A novel binding pocket of cyclin-dependent kinase 2. Proteins Struct. Funct. Bioinform..

[59] Li Y., Zhang J., Gao W., Zhang L., Pan Y., Zhang S., Wang Y. (2015). Insights on structural characteristics and ligand binding mechanisms of CDK2. Int. J. Mol. Sci..

[60] Liang J.-W., Wang M.-Y., Wang S., Li S.-L., Li W.-Q., Meng F.-H. (2020). Identification of novel CDK2 inhibitors by a multistage virtual screening method based on SVM, pharmacophore and docking model. J. Enzyme Inhib. Med. Chem..

[61] Fathalla A., Zaghary W.A., Radwan H.H., Awad S.M., Mohamed M.S. (2002). Synthesis of new 2-thiouracil-5-sulfonamide derivatives with biological activity. Arch. Pharm. Res..

[62] Shen L., Hu J., Wang H., Wang A., Lai Y., Kang Y. (2015). Synthesis and biological evaluation of novel uracil and 5-fluorouracil-1-yl acetic acid-colchicine conjugate. Chem. Res. Chin. Univ..

[63] Guan X.W., Xu X.H., Feng S.L., Tang Z.B., Chen S.W., Hui L. (2016). Synthesis of hybrid 4-deoxypodophyllotoxin-5-fluorouracil compounds that inhibit cellular migration and induce cell cycle arrest. Bioorg. Med. Chem. Lett..

[64] Radwan A.A., Alanazi F.K. (2014). Design and synthesis of new cholesterol-conjugated 5-Fluorouracil: A novel potential delivery system for cancer treatment. Molecules.

[65] Hassan G.S., Kadry H.H., Abou-Seri S.M., Ali M.M., Mahmoud A.E. (2011). Synthesis and in vitro cytotoxic activity of novel pyrazolo[3,4-d]pyrimidines and related pyrazole hydrazones toward breast adenocarcinoma MCF-7 cell line. Bioorg. Med. Chem..

[66] Zheng L.W., Wu L.L., Zhao B.X., Dong W.L., Miao J.Y. (2009). Synthesis of novel substituted pyrazole-5-carbohydrazide hydrazone derivatives and discovery of a potent apoptosis inducer in A549 lung cancer cells. Bioorg. Med. Chem..

[67] Hafez H.N., El-Gazzar A.R. (2009). Synthesis and antitumor activity of substituted triazolo[4,3-a]pyrimidin-6-sulfonamide with an incorporated thiazolidinone moiety. Bioorg. Med. Chem. Lett..

[68] Badisa R., Ayuk-Takem L., Ikediobi C., Walker E. (2006). Selective anticancer activity of pure licamichauxiioic-B acid in cultured cell lines. Pharm. Biol..

[69] Asghar U., Witkiewicz A.K., Turner N.C., Knudsen E.S. (2015). The history and future of targeting cyclin-dependent kinases in cancer therapy. Nat. Rev. Drug Discov..

[70] Pollet M., Shaik S., Mescher M., Frauenstein K., Tigges J., Braun S.A., Sondenheimer K., Kaveh M., Bruhs A., Meller S. (2018). The AHR represses nucleotide excision repair and apoptosis and contributes to UV-induced skin carcinogenesis. Cell Death Differ..

[71] Abaza M.S., Bahman A.M., Al-Attiyah R.J. (2014). Valproic acid, an anti-epileptic drug and a histone deacetylase inhibitor, in combination with proteasome inhibitors exerts antiproliferative, pro-apoptotic and chemosensitizing effects in human colorectal cancer cells: Underlying molecular mechanisms. Int. J. Mol. Med..

[72] Abd El-Hameed R.H., Sayed A.I. (2018). Synthesis of novel pyrrolopyrimidine derivatives as CDK2 inhibitors. Pharmacophore.

[73] Zander U., Hoffmann G., Cornaciu I., Marquette J.P., Papp G., Landret C., Seroul G., Sinoir J., Rower M., Felisaz F. (2016). Automated harvesting and processing of protein crystals through laser photoablation. Acta Crystallogr. D Struct. Biol..

