# Supplementary material for: Synthesis of Novel 2-Thiouracil-5-Sulfonamide Derivatives as Potent Inducers of Cell Cycle Arrest and CDK2A Inhibition Supported by Molecular Docking
Source: Int J Mol Sci. 2021 Nov 4;22(21):11957. doi: 10.3390/ijms222111957 (PMC8584424; doi:10.3390/ijms222111957)
Supplement: Supplementary file 1 [file ijms-22-11957-s001.zip › ijms-1415103-supplementary.pdf]

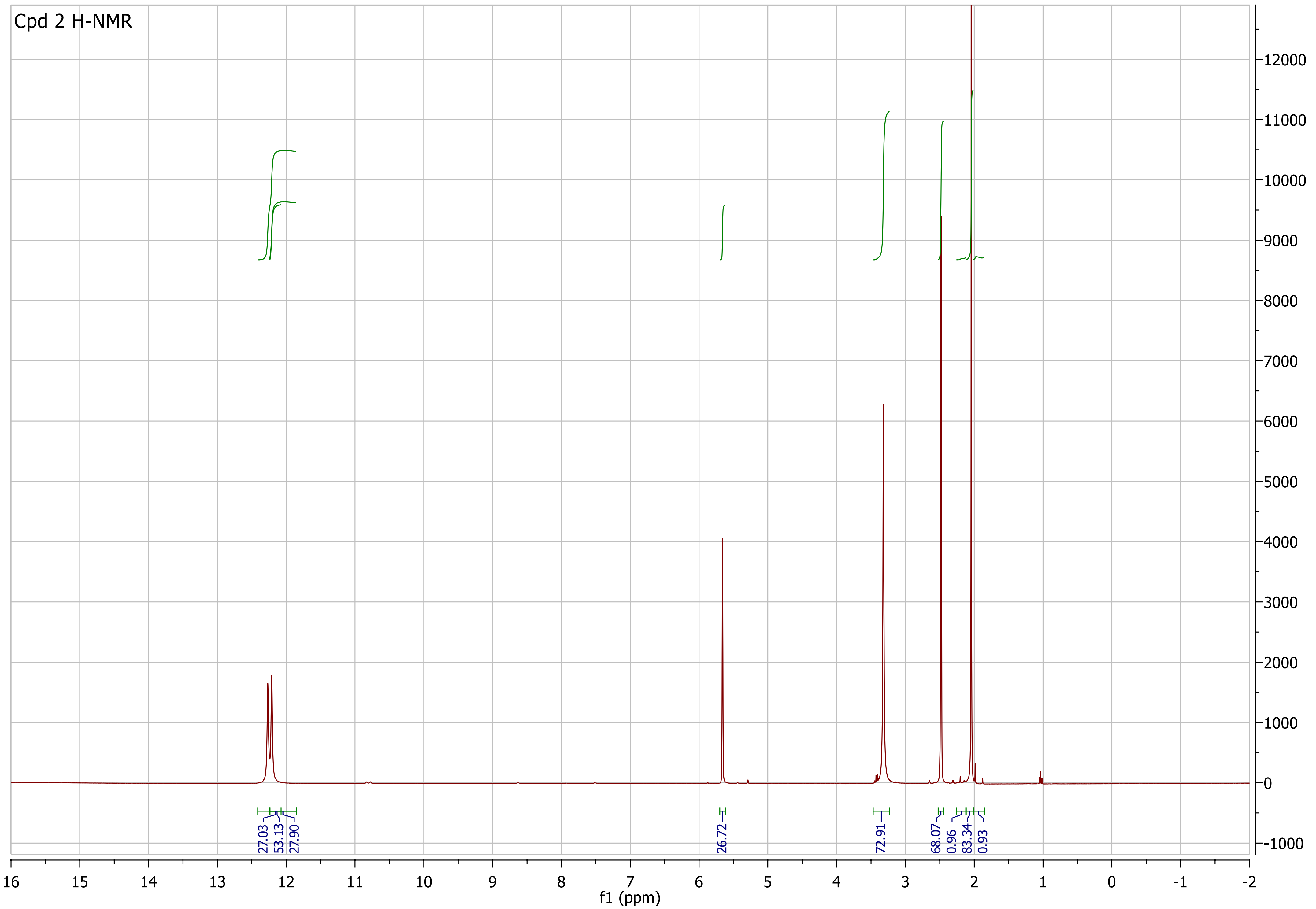

Cpd 2 H-NMR
27.03
53.13
27.90
26.72
72.91
68.07
0.96
83.34
0.93
f1 (ppm)

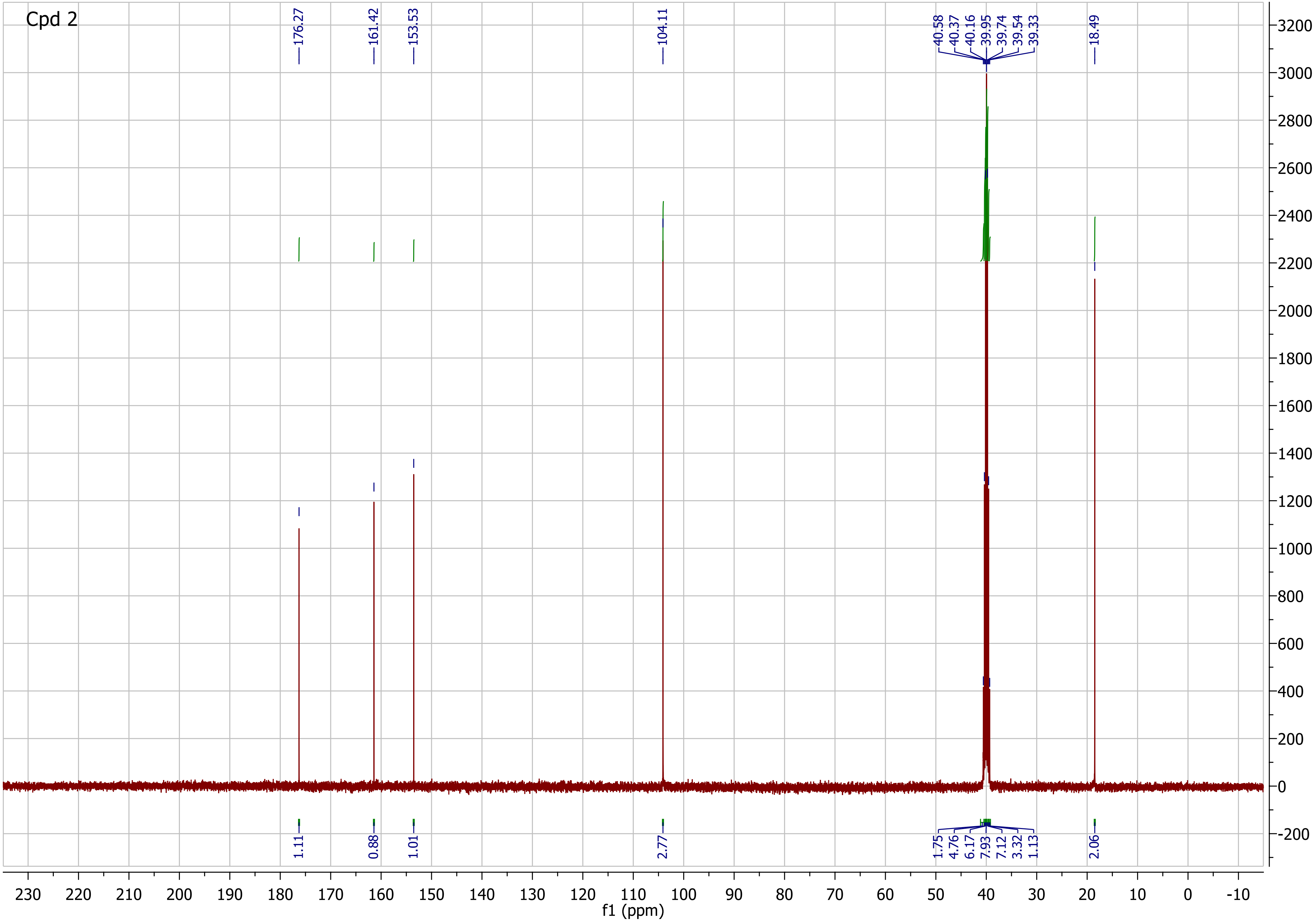

Cpd 2
176.27
161.42
153.53
104.11
40.58
40.37
40.16
39.95
39.74
39.54
39.33
18.49
1.11
0.88
1.01
2.77
1.75
4.76
6.17
7.93
7.12
3.32
1.13
2.06
f1 (ppm)

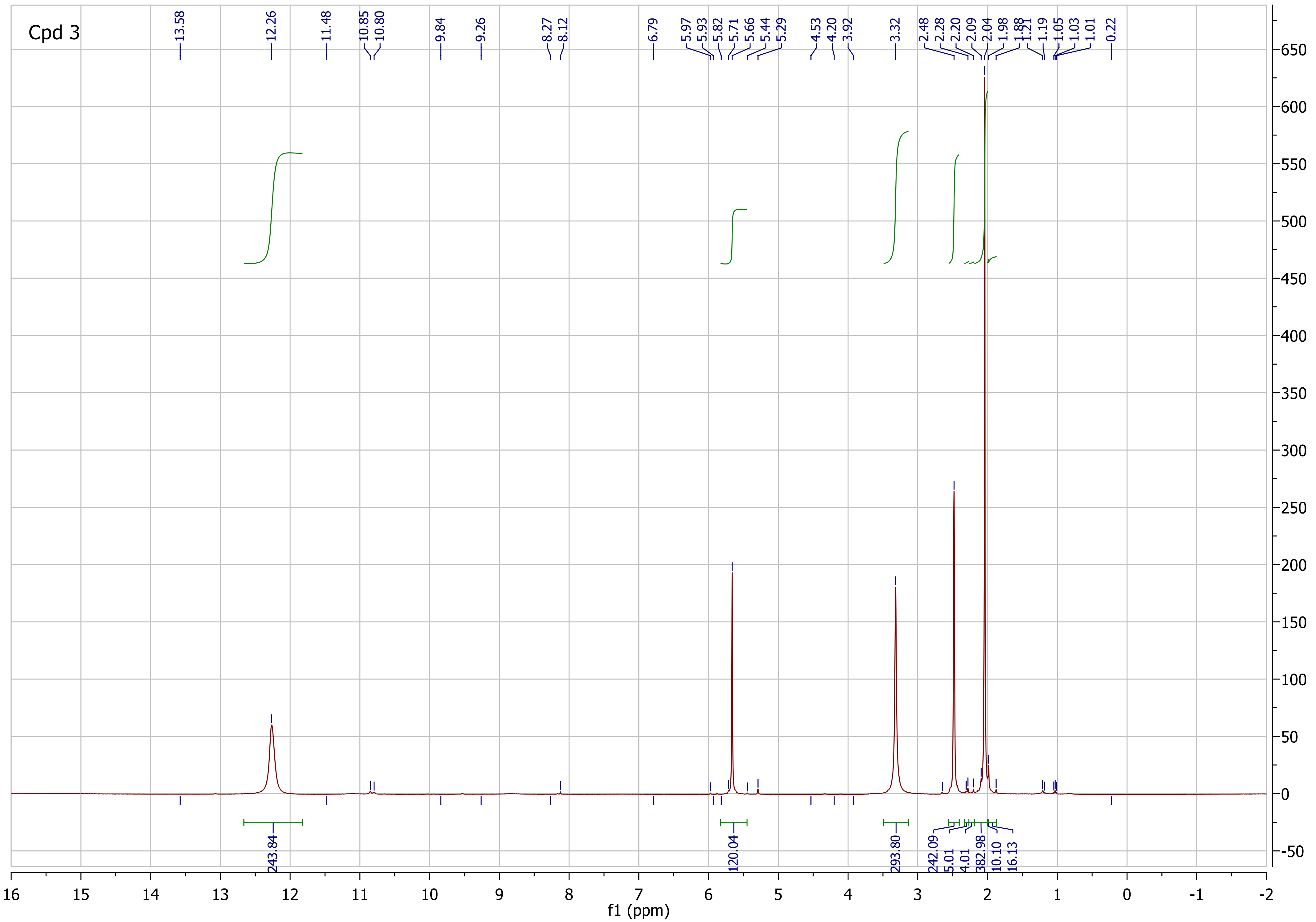

Cpd 3
13.58
12.26
11.48
10.85
10.80
9.84
9.26
8.27
8.12
6.79
5.97
5.93
5.82
5.71
5.66
5.44
5.29
4.53
4.20
3.92
3.32
2.48
2.28
2.20
2.09
2.04
1.98
1.88
1.21
1.19
1.05
1.03
1.01
0.22
243.84
120.04
293.80
242.09
5.01
4.01
382.98
10.10
16.13
f1 (ppm)

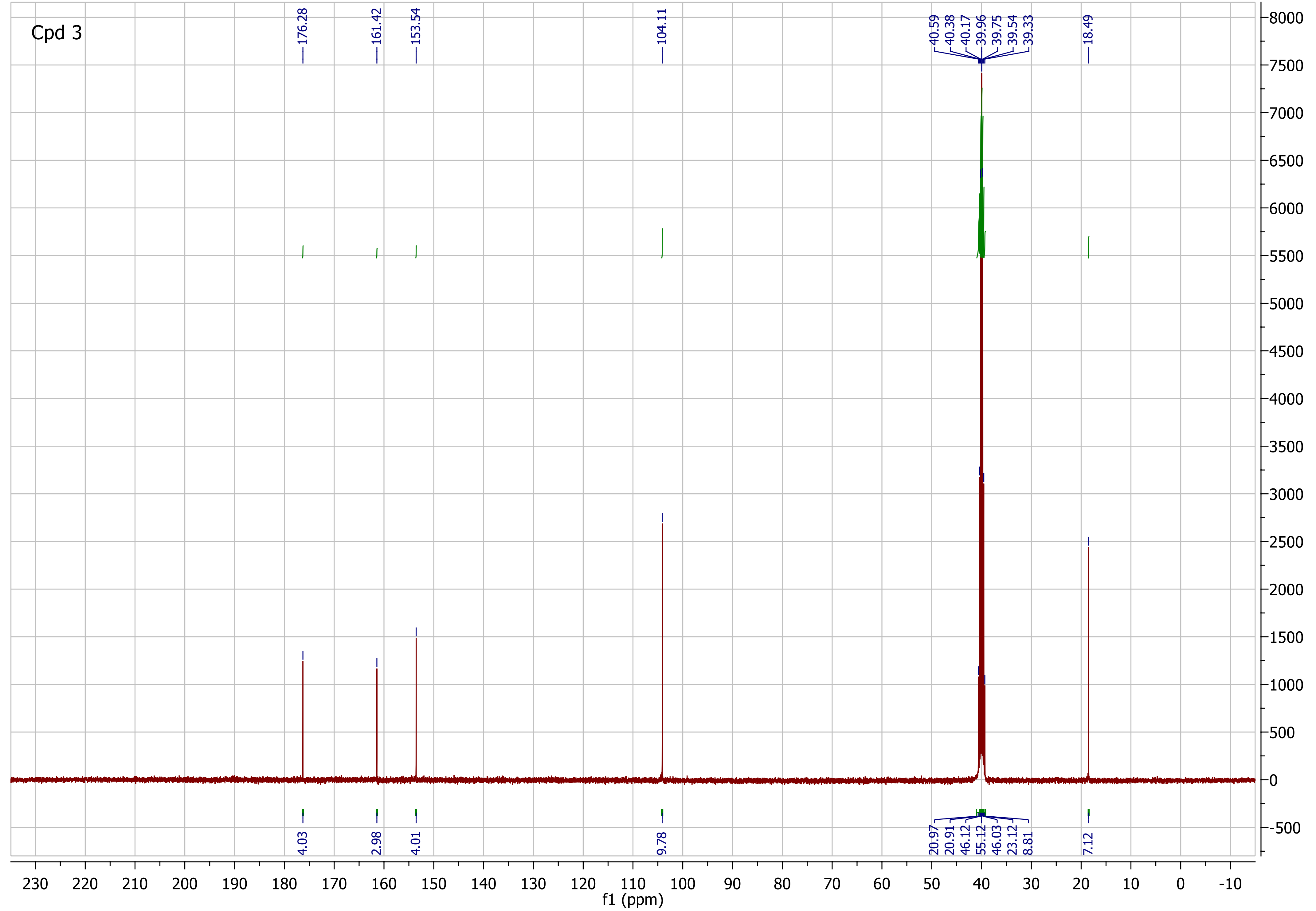

Cpd 3
176.28
161.42
153.54
104.11
40.59
40.38
40.17
39.96
39.75
39.54
39.33
18.49
4.03
2.98
4.01
9.78
20.97
20.91
46.12
55.12
46.03
23.12
8.81
7.12
f1 (ppm)

Cpd 4 H-NMR

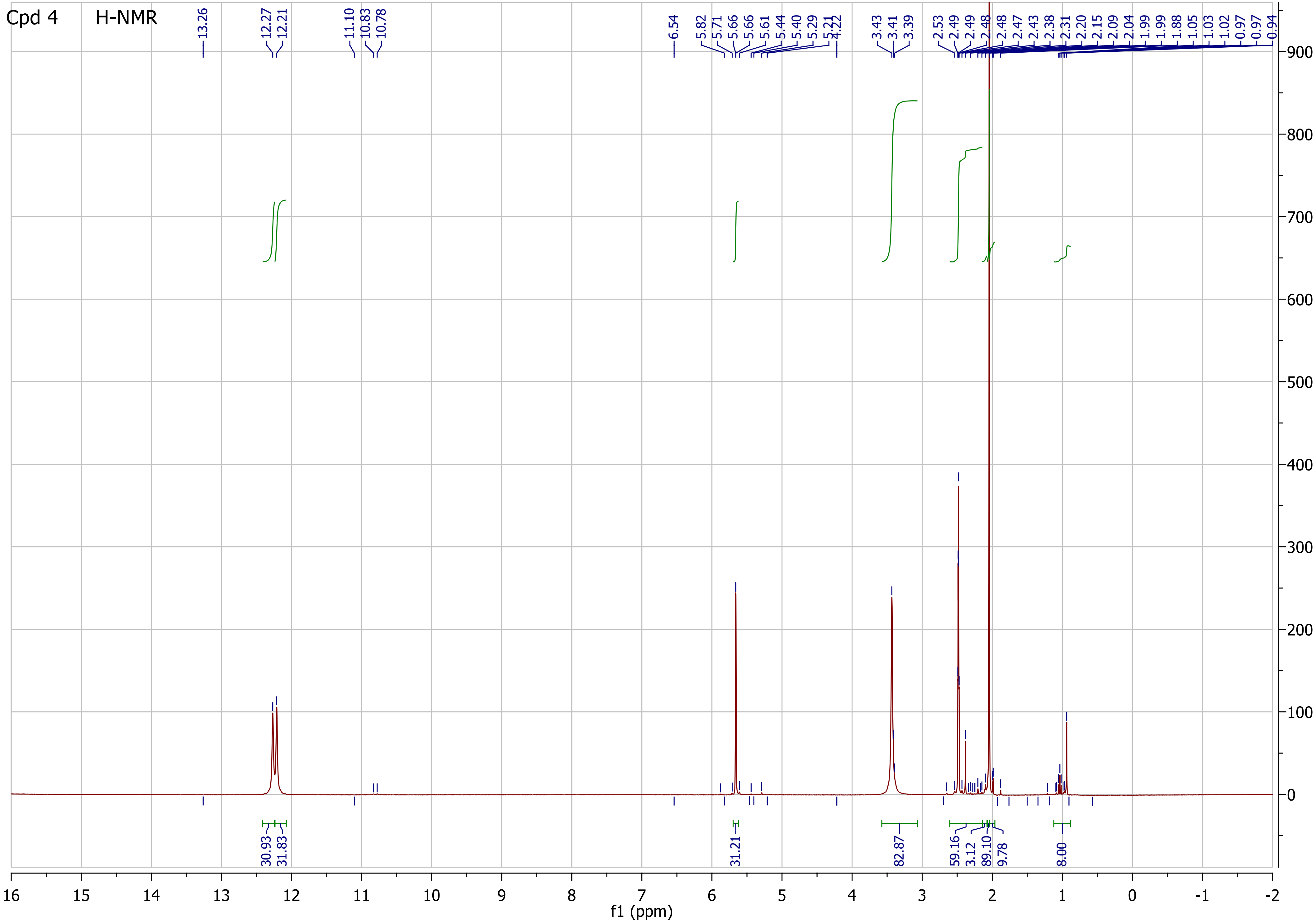

13.26
12.27
12.21
11.10
10.83
10.78
6.54
5.82
5.71
5.66
5.66
5.61
5.44
5.40
5.29
5.21
4.22
3.43
3.41
3.39
2.53
2.49
2.49
2.48
2.48
2.47
2.43
2.38
2.31
2.20
2.15
2.09
2.04
1.99
1.99
1.88
1.05
1.03
1.02
0.97
0.97
0.94
30.93
31.83
31.21
82.87
59.16
3.12
89.10
9.78
8.00
f1 (ppm)

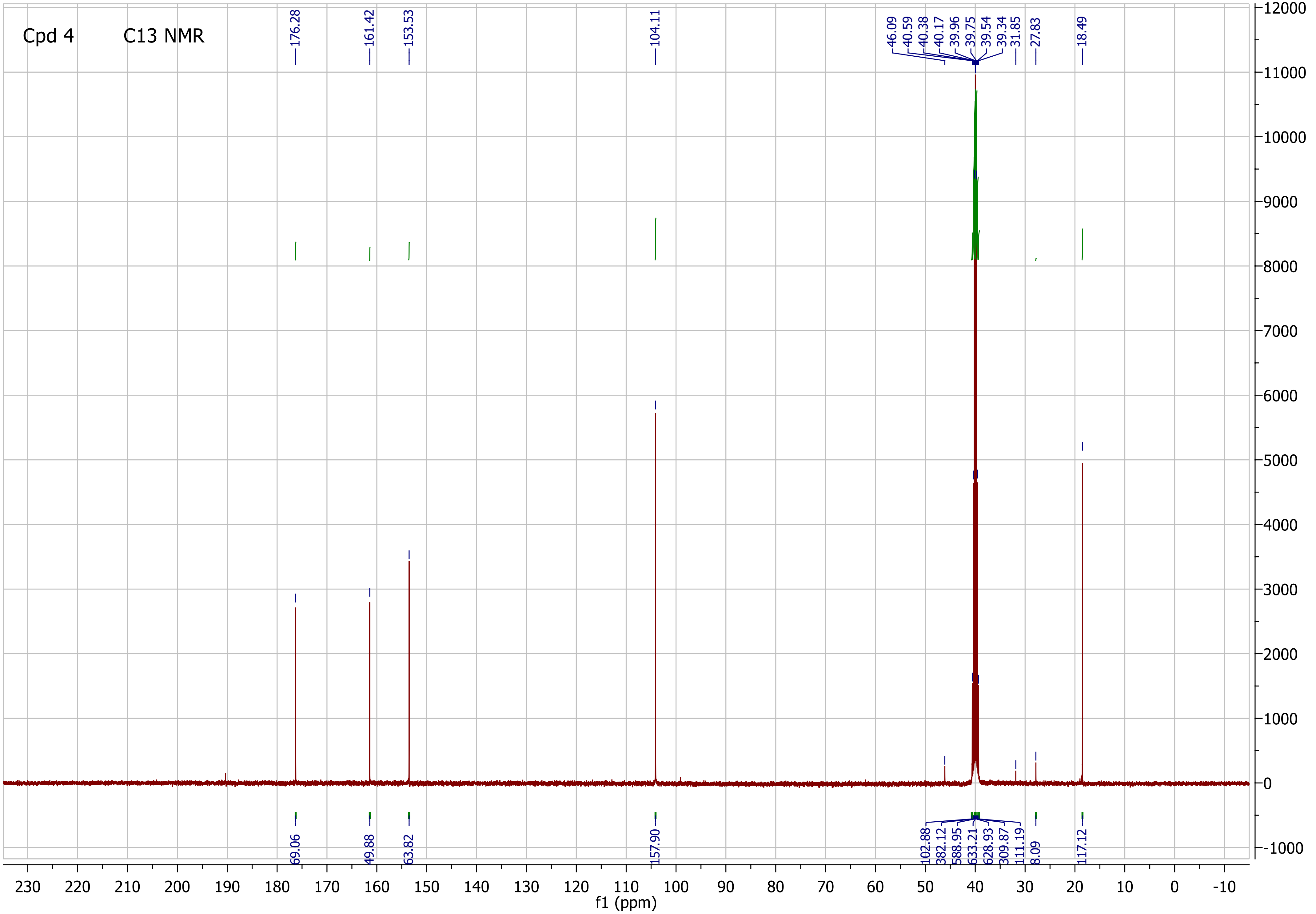
Cpd 4
C13 NMR
176.28
161.42
153.53
104.11
46.09
40.59
40.38
40.17
39.96
39.75
39.54
39.34
31.85
27.83
18.49
69.06
49.88
63.82
157.90
102.88
382.12
588.95
633.21
628.93
309.87
111.19
8.09
117.12
f1 (ppm)

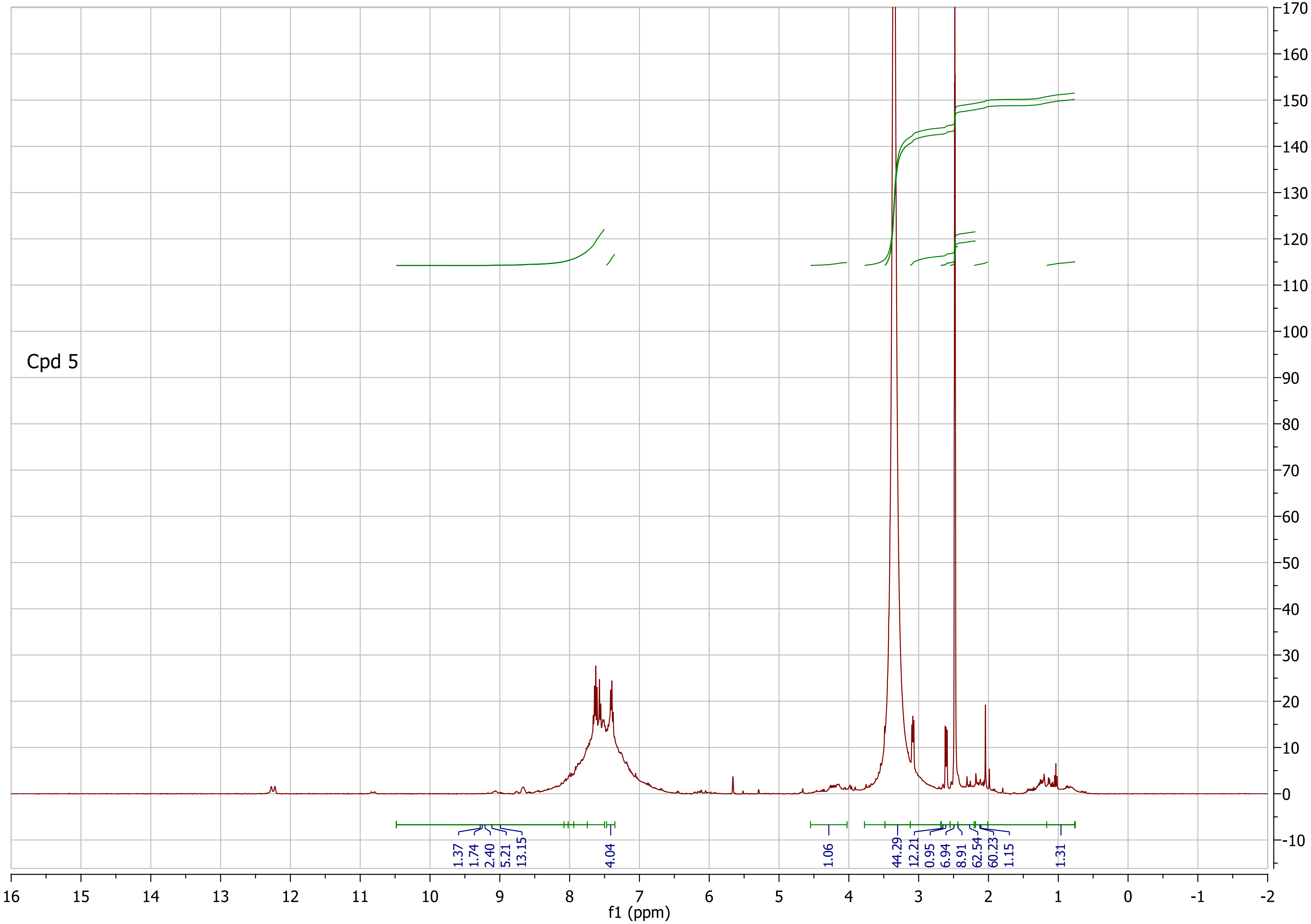

Cpd 5
f1 (ppm)
1.37
1.74
2.40
5.21
13.15
4.04
1.06
44.29
12.21
0.95
6.94
8.91
62.54
60.23
1.15
1.31

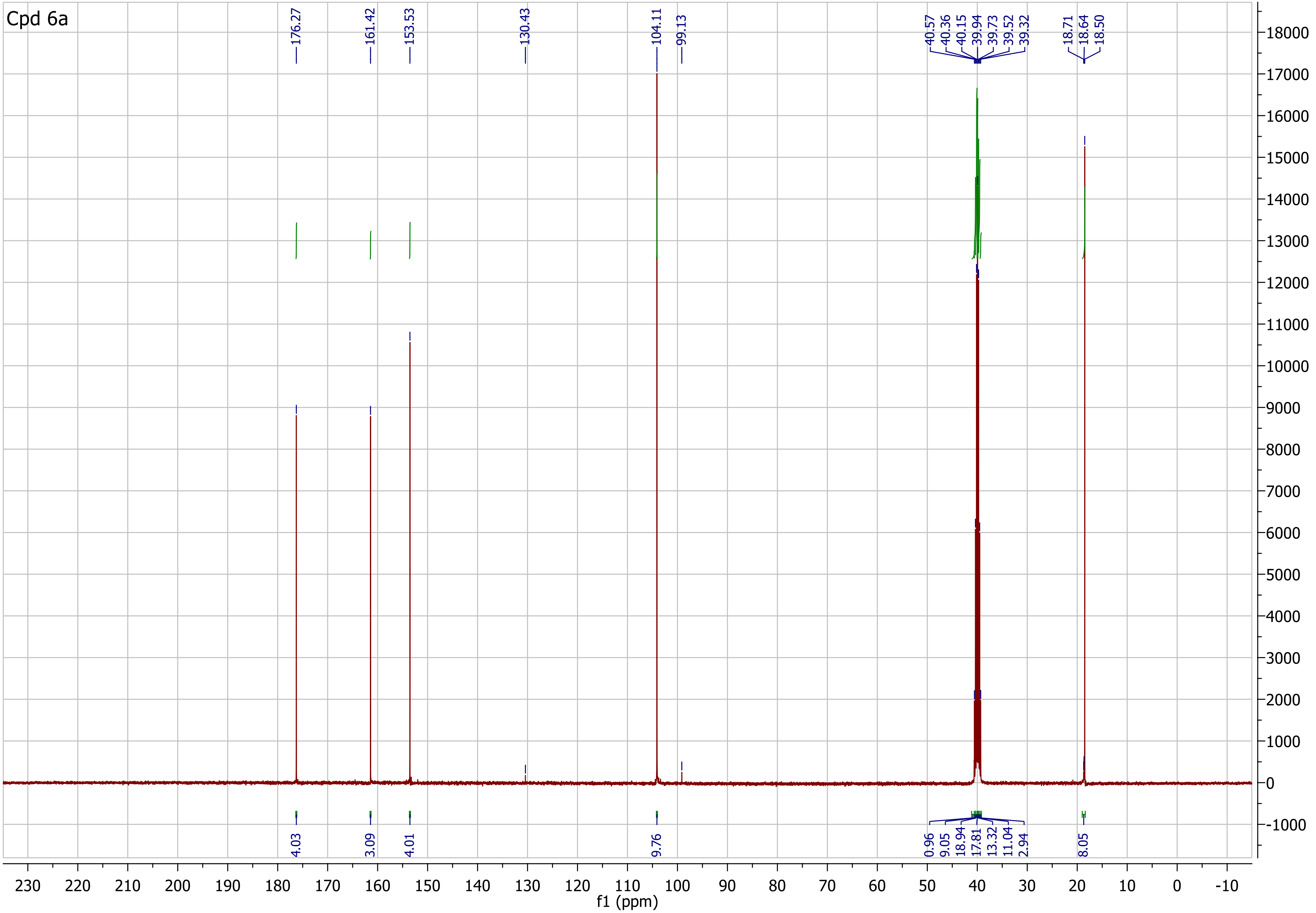
Cpd 6a
176.27
161.42
153.53
130.43
104.11
99.13
40.57
40.36
40.15
39.94
39.73
39.52
39.32
18.71
18.64
18.50
4.03
3.09
4.01
9.76
0.96
9.05
18.94
17.81
13.32
11.04
2.94
8.05
f1 (ppm)

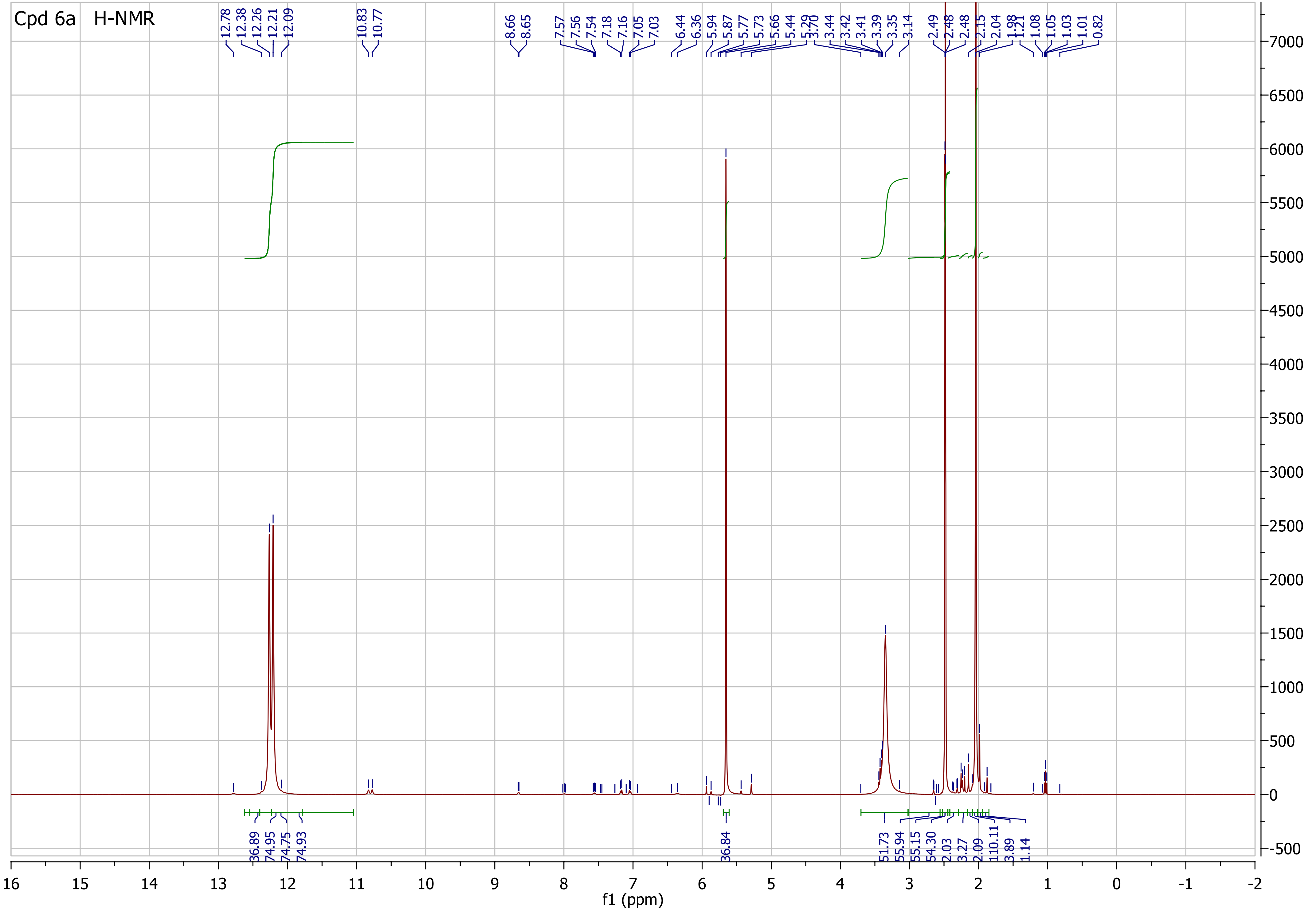

Cpd 6a H-NMR
12.78
12.38
12.26
12.21
12.09
10.83
10.77
8.66
8.65
7.57
7.56
7.54
7.18
7.16
7.05
7.03
6.44
6.36
5.94
5.87
5.77
5.73
5.66
5.44
5.29
3.70
3.44
3.42
3.41
3.39
3.35
3.14
2.49
2.48
2.48
2.15
2.04
1.98
1.21
1.08
1.05
1.03
1.01
0.82
36.89
74.95
74.75
74.93
36.84
51.73
55.94
55.15
54.30
2.03
3.27
2.09
110.11
3.89
1.14
f1 (ppm)

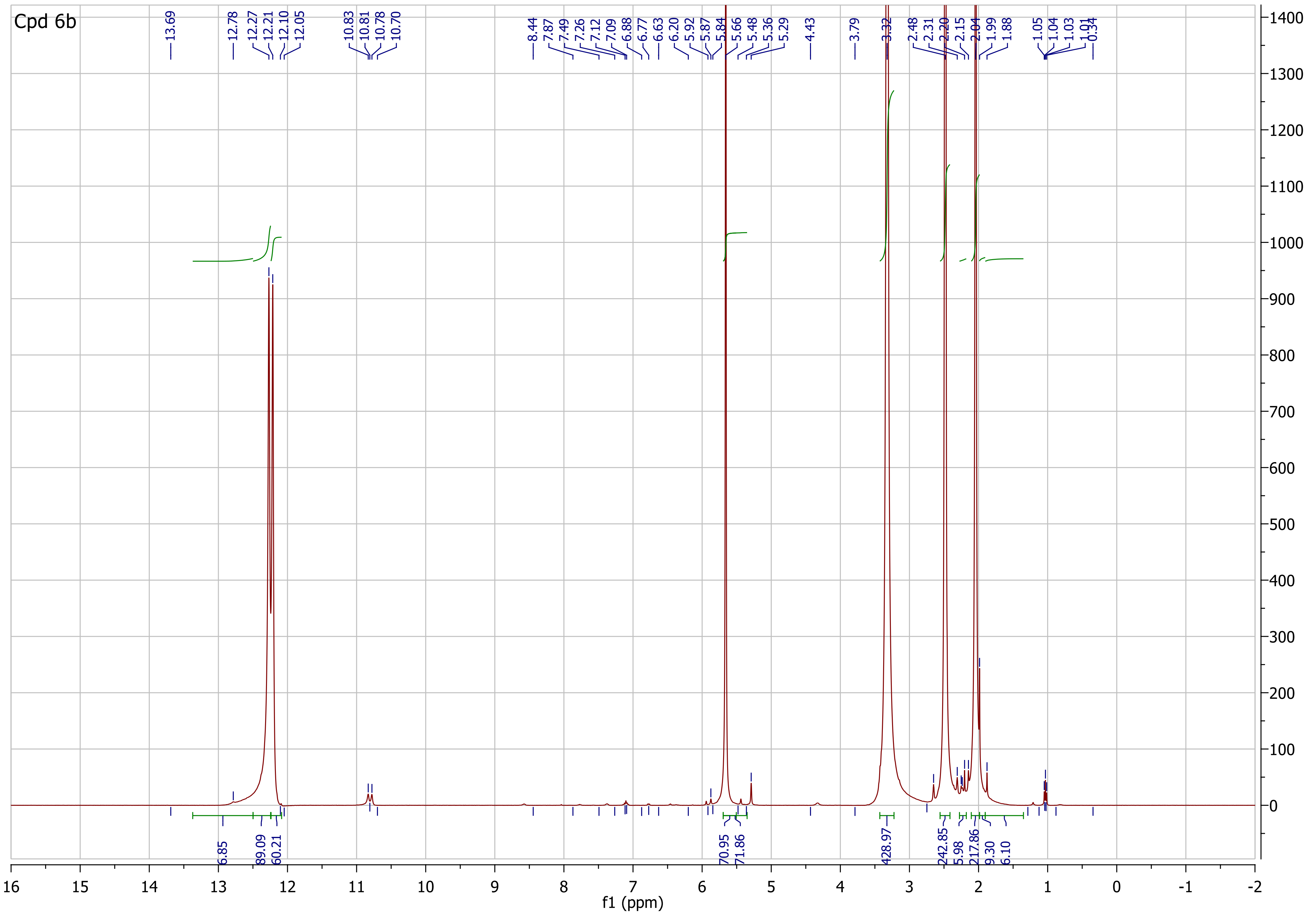

Cpd 6b
13.69
12.78
12.27
12.21
12.10
12.05
10.83
10.81
10.78
10.70
8.44
7.87
7.49
7.26
7.12
7.09
6.88
6.77
6.63
6.20
5.92
5.87
5.84
5.66
5.48
5.36
5.29
4.43
3.79
3.32
2.48
2.31
2.20
2.15
2.04
1.99
1.88
1.05
1.04
1.03
1.01
0.34
6.85
89.09
60.21
70.95
71.86
428.97
242.85
5.98
217.86
9.30
6.10
f1 (ppm)

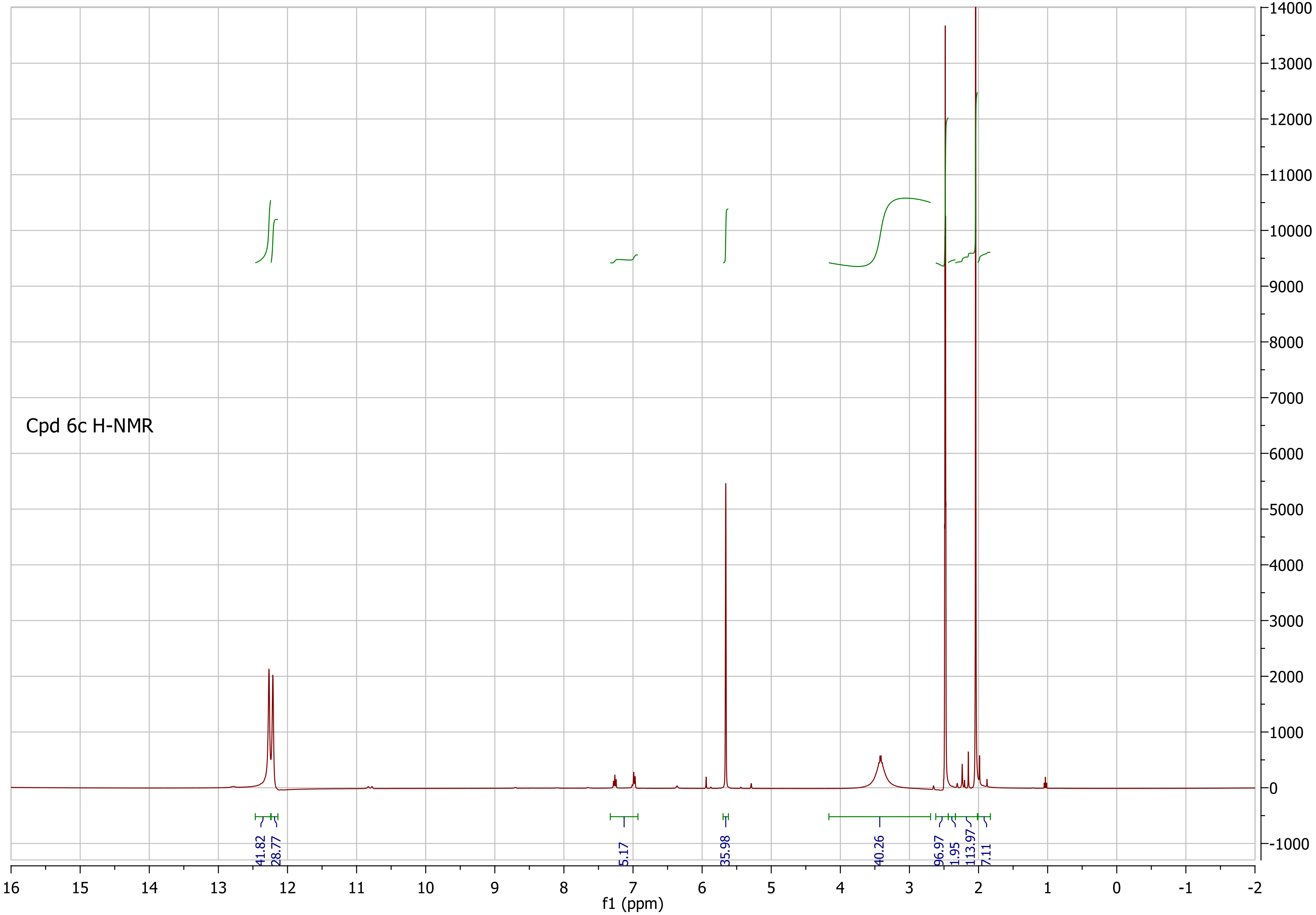

Cpd 6c H-NMR
f1 (ppm)
41.82
28.77
5.17
35.98
40.26
96.97
1.95
113.97
7.11

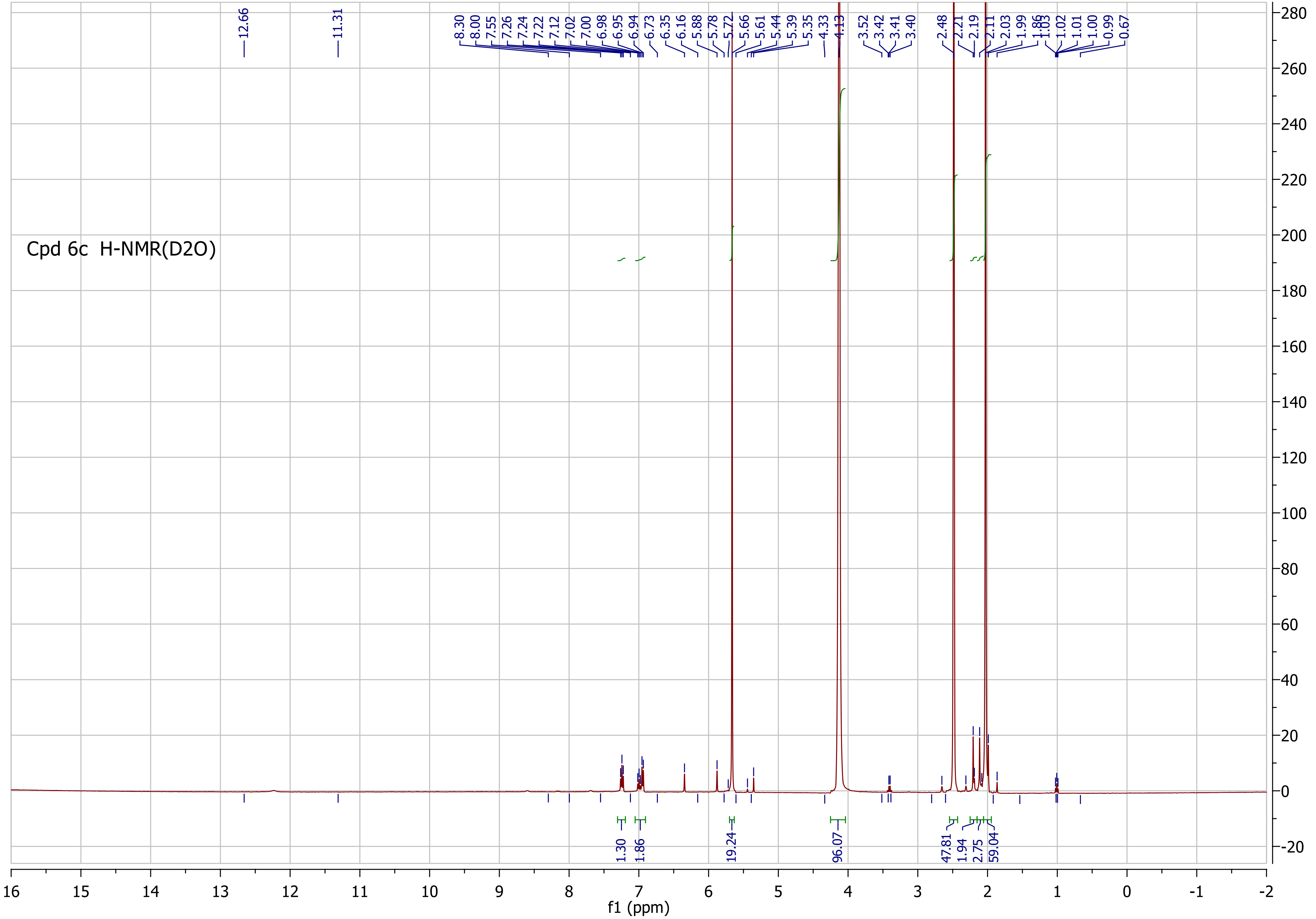
Cpd 6c H-NMR(D2O)
f1 (ppm)

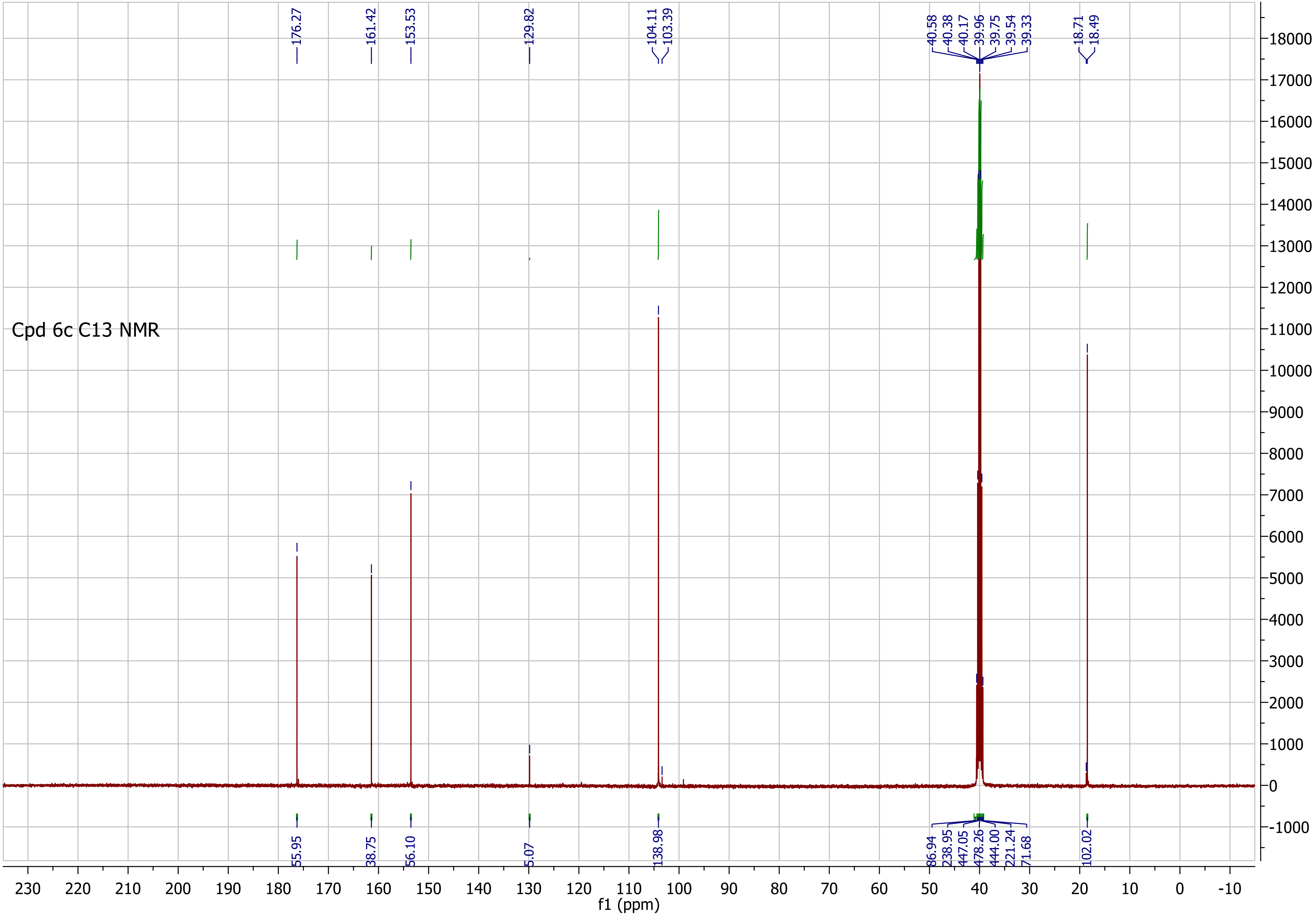
Cpd 6c C13 NMR
176.27
161.42
153.53
129.82
104.11
103.39
40.58
40.38
40.17
39.96
39.75
39.54
39.33
18.71
18.49
55.95
38.75
56.10
5.07
138.98
86.94
238.95
447.05
478.26
444.00
221.24
71.68
102.02
f1 (ppm)

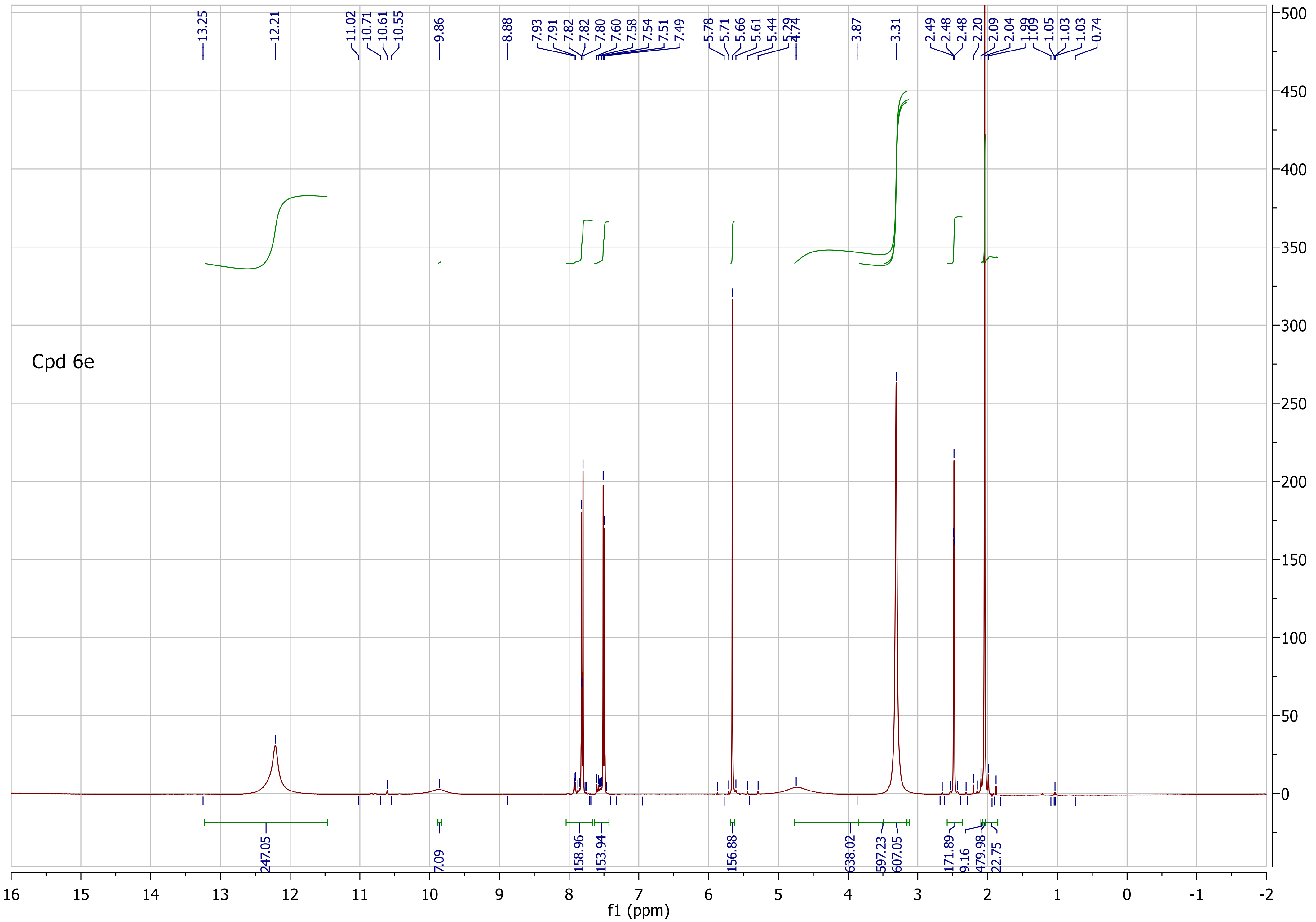
Cpd 6e
13.25
12.21
11.02
10.71
10.61
10.55
9.86
8.88
7.93
7.91
7.82
7.82
7.80
7.60
7.58
7.54
7.51
7.49
5.78
5.71
5.66
5.61
5.44
5.29
4.74
3.87
3.31
2.49
2.48
2.48
2.20
2.09
2.04
1.99
1.09
1.05
1.03
1.03
0.74
247.05
7.09
158.96
153.94
156.88
638.02
597.23
607.05
171.89
9.16
479.98
22.75
f1 (ppm)

Cpd 7a C13-NMR

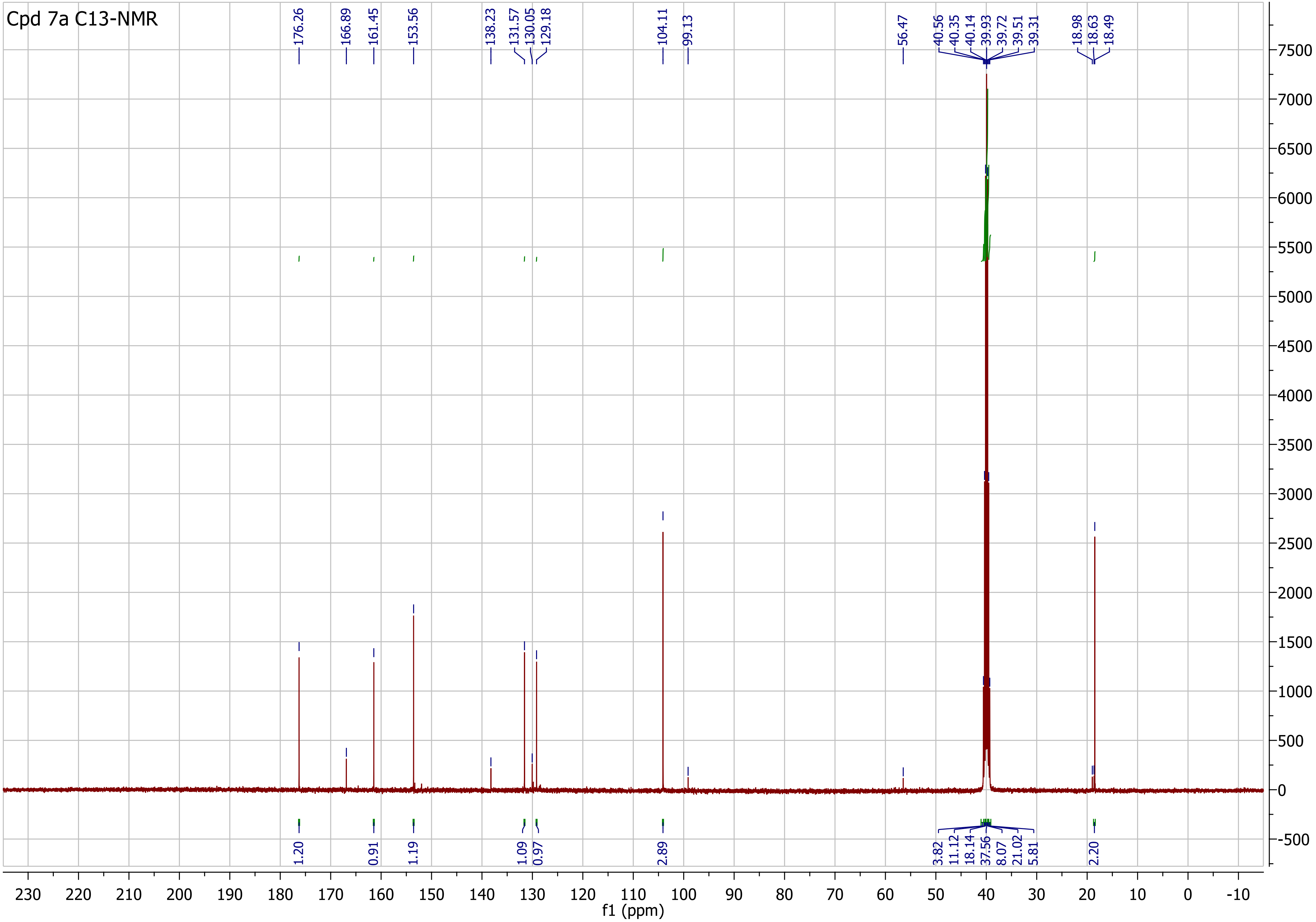

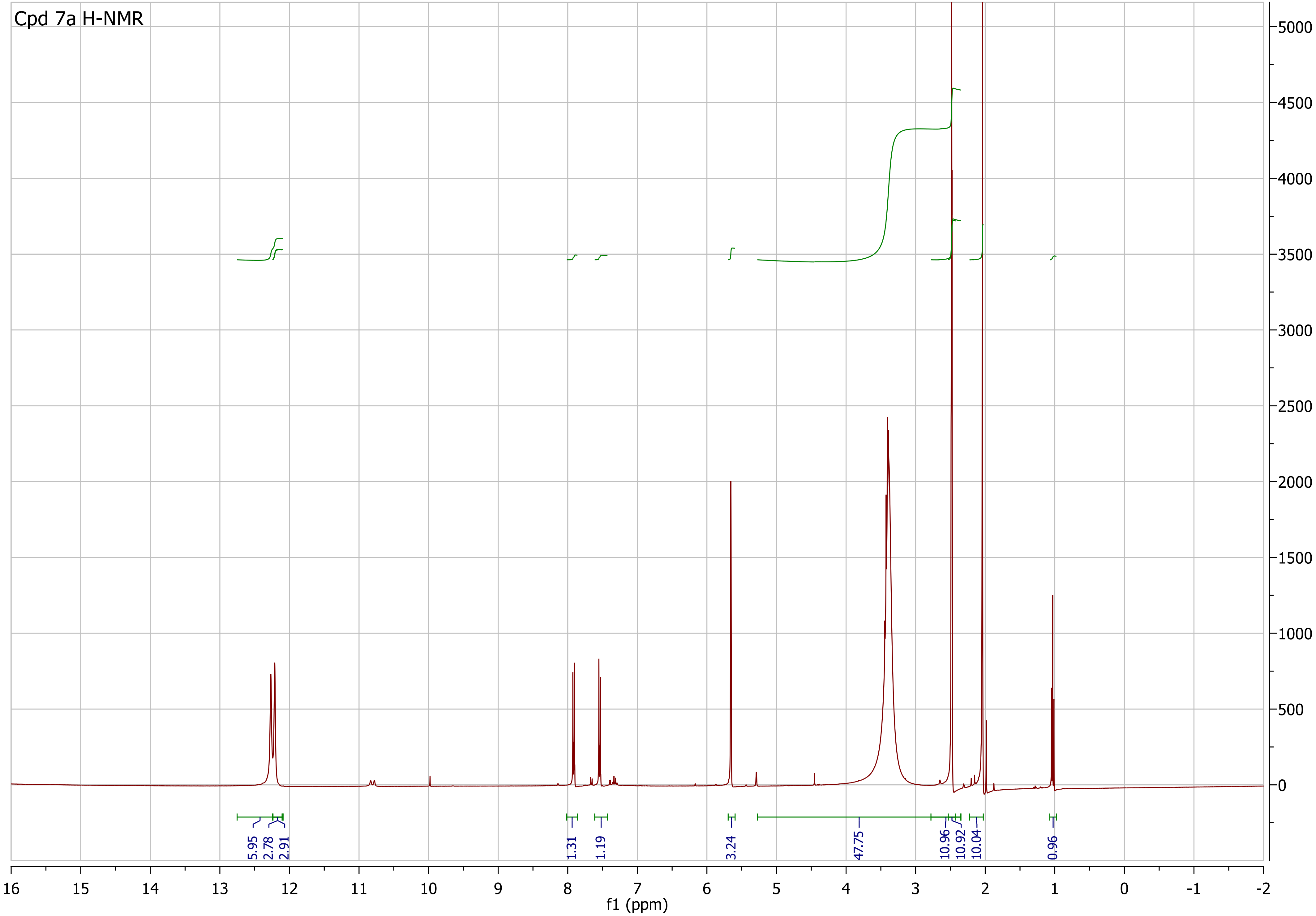

Cpd 7a H-NMR
5000
4500
4000
3500
3000
2500
2000
1500
1000
500
0
5.95
2.78
2.91
1.31
1.19
3.24
47.75
10.96
10.92
10.04
0.96
16
15
14
13
12
11
10
9
8
7
6
5
4
3
2
1
0
-1
-2
f1 (ppm)

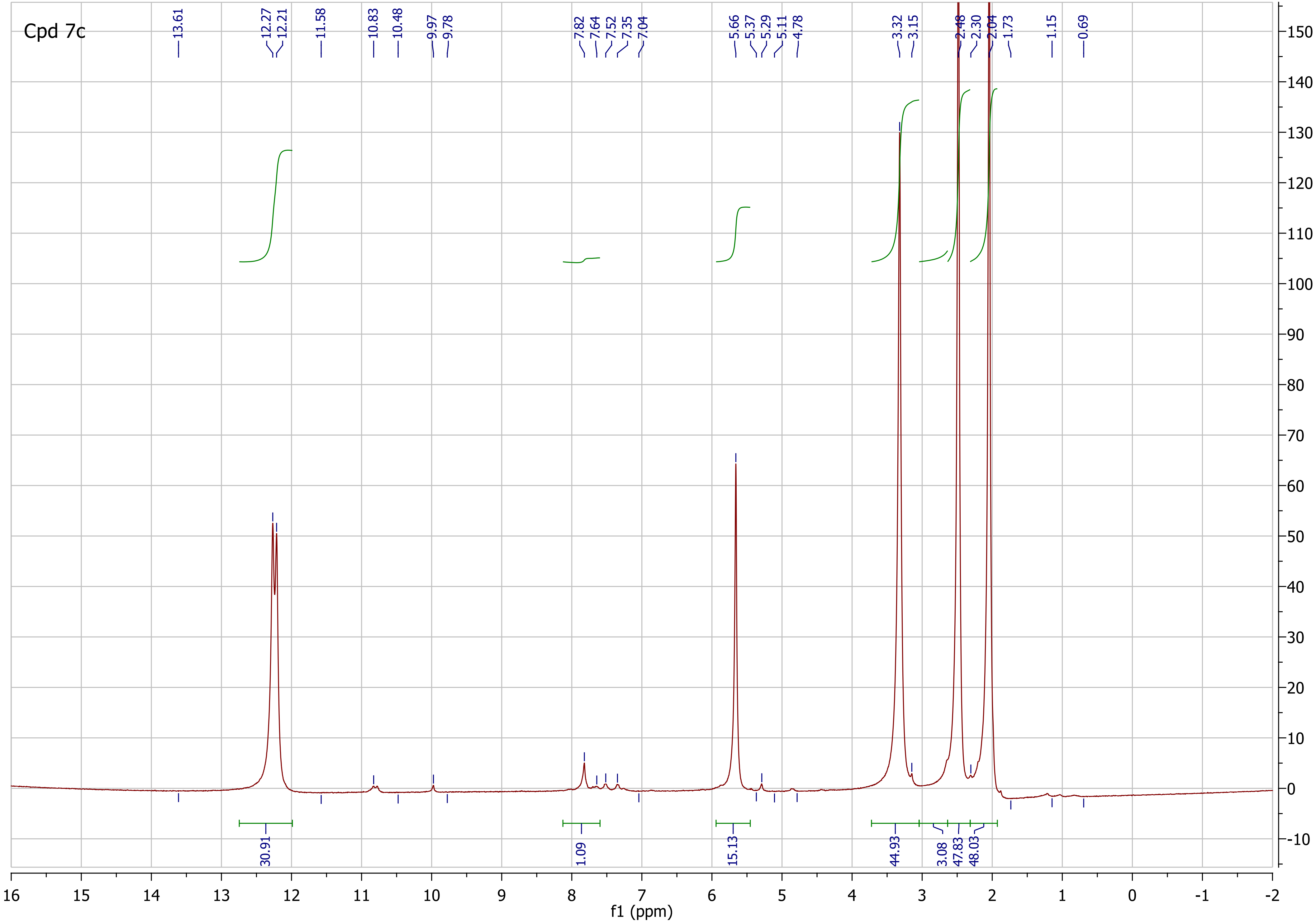

Cpd 7c
13.61
12.27
12.21
11.58
10.83
10.48
9.97
9.78
7.82
7.64
7.52
7.35
7.04
5.66
5.37
5.29
5.11
4.78
3.32
3.15
2.48
2.30
2.04
1.73
1.15
0.69
30.91
1.09
15.13
44.93
3.08
47.83
48.03
f1 (ppm)

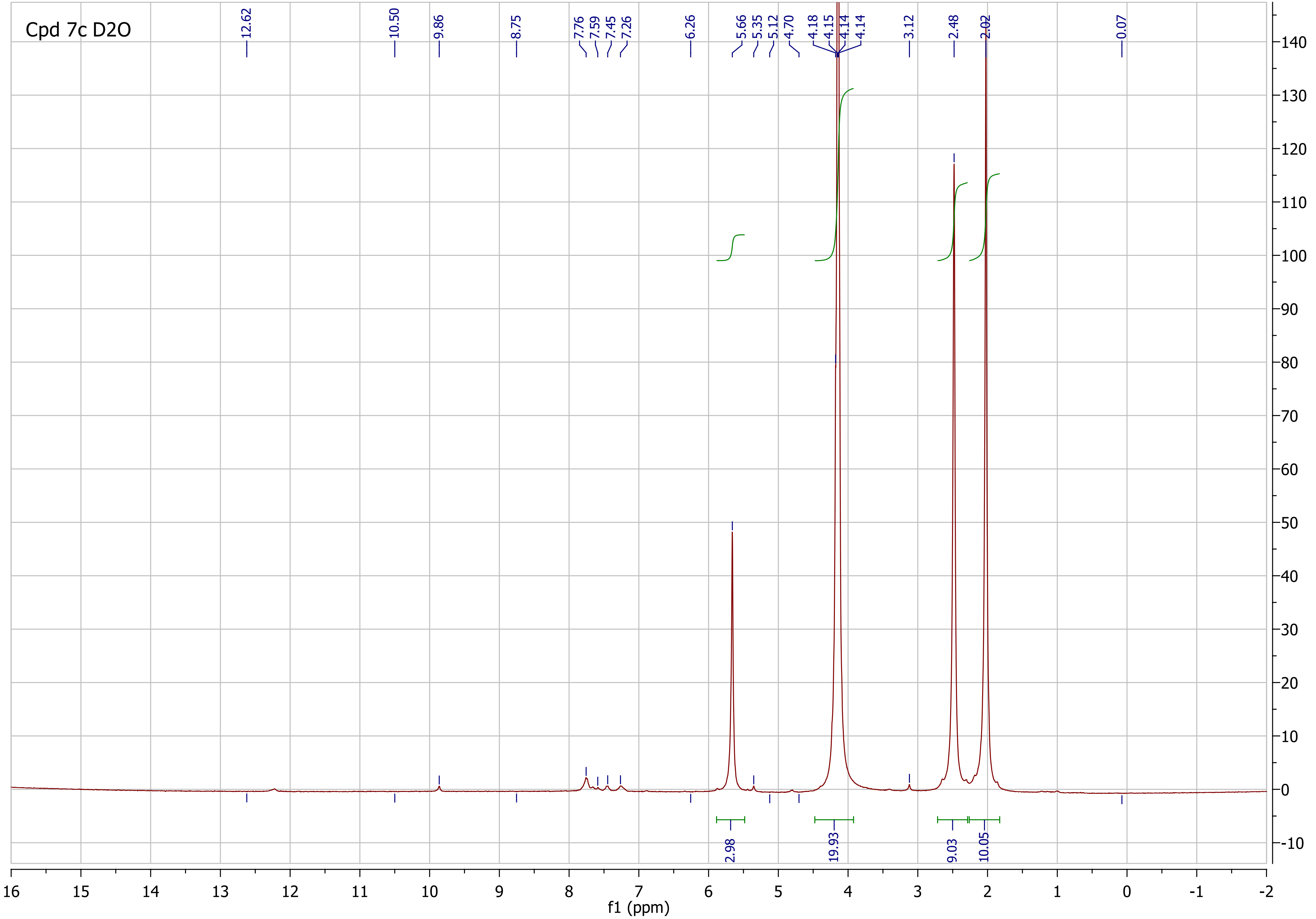

Cpd 7c D2O
12.62
10.50
9.86
8.75
7.76
7.59
7.45
7.26
6.26
5.66
5.35
5.12
4.70
4.18
4.15
4.14
4.14
3.12
2.48
2.02
0.07
2.98
19.93
9.03
10.05
f1 (ppm)

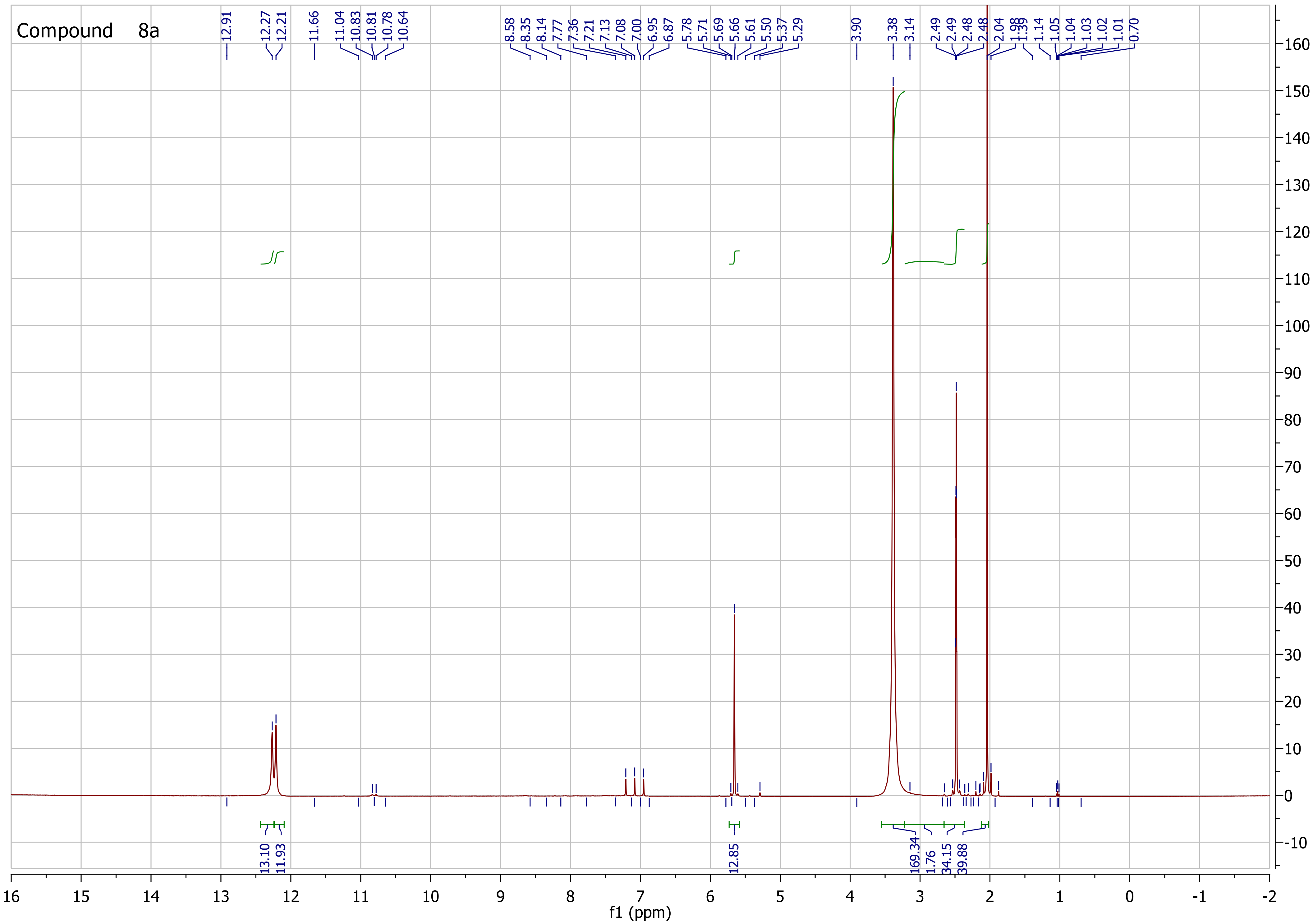

Compound 8a
12.91
12.27
12.21
11.66
11.04
10.83
10.81
10.78
10.64
8.58
8.35
8.14
7.77
7.36
7.21
7.13
7.08
7.00
6.95
6.87
5.78
5.71
5.69
5.66
5.61
5.50
5.37
5.29
3.90
3.38
3.14
2.49
2.49
2.48
2.48
2.04
1.98
1.39
1.14
1.05
1.04
1.03
1.02
1.01
0.70
13.10
11.93
12.85
169.34
1.76
34.15
39.88
f1 (ppm)

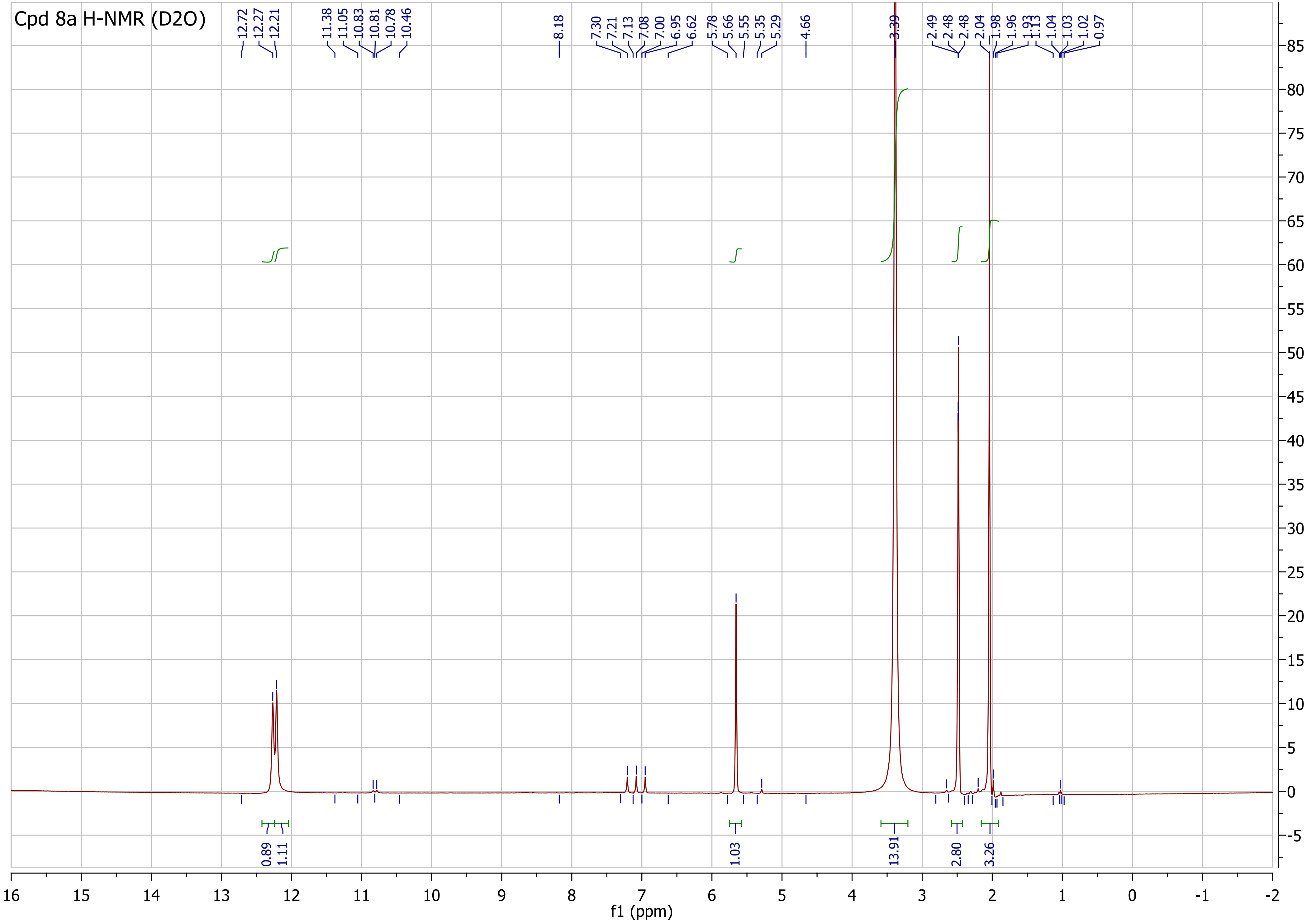
Cpd 8a H-NMR (D2O)
12.72
12.27
12.21
11.38
11.05
10.83
10.81
10.78
10.46
8.18
7.30
7.21
7.13
7.08
7.00
6.95
6.62
5.78
5.66
5.55
5.35
5.29
4.66
3.39
2.49
2.48
2.48
2.04
1.98
1.96
1.93
1.13
1.04
1.03
1.02
0.97
0.89
1.11
1.03
13.91
2.80
3.26
f1 (ppm)

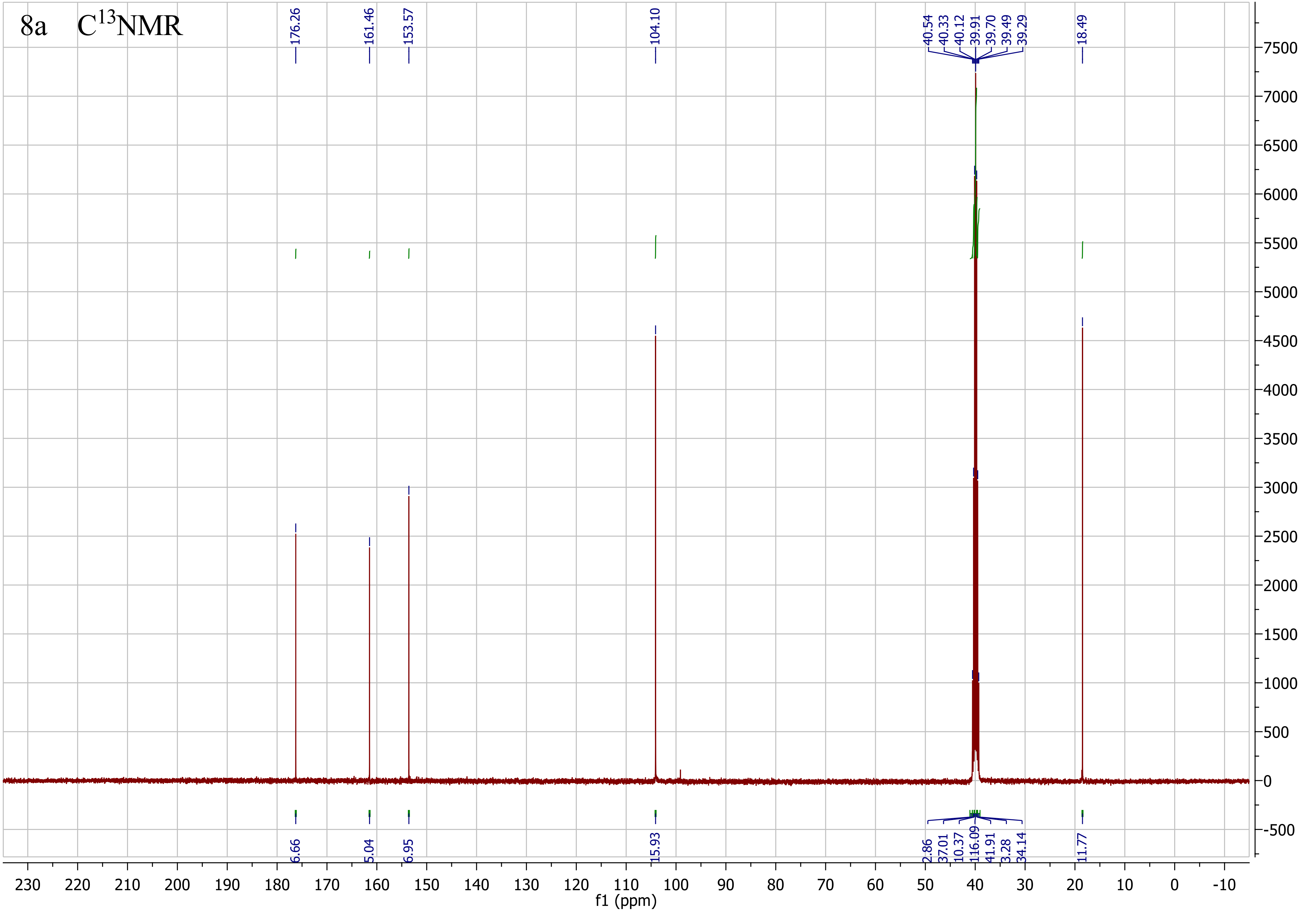

8a C13NMR
176.26
161.46
153.57
104.10
40.54
40.33
40.12
39.91
39.70
39.49
39.29
18.49
6.66
5.04
6.95
15.93
2.86
37.01
10.37
116.09
41.91
3.28
34.14
11.77
f1 (ppm)

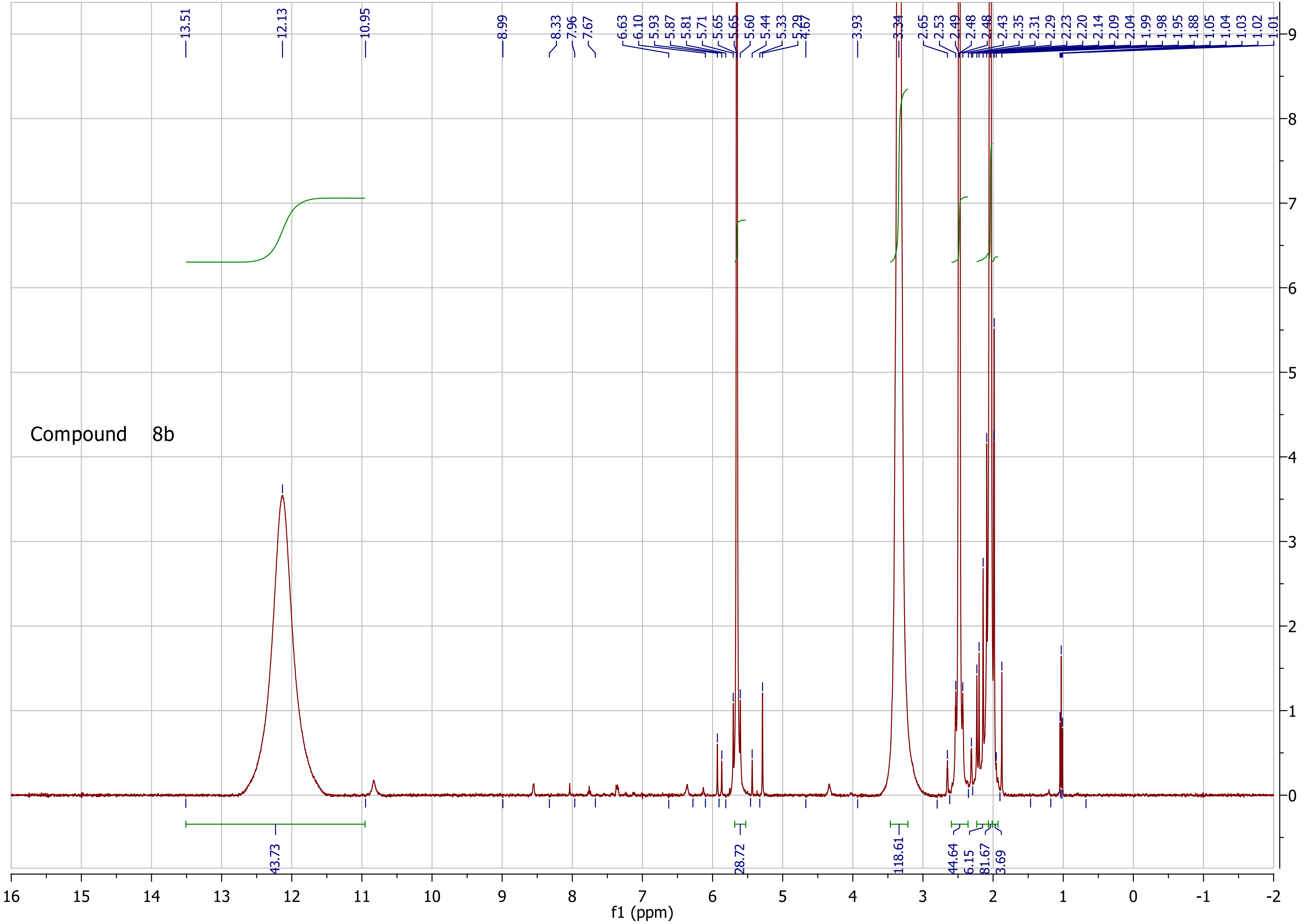

Compound 8b

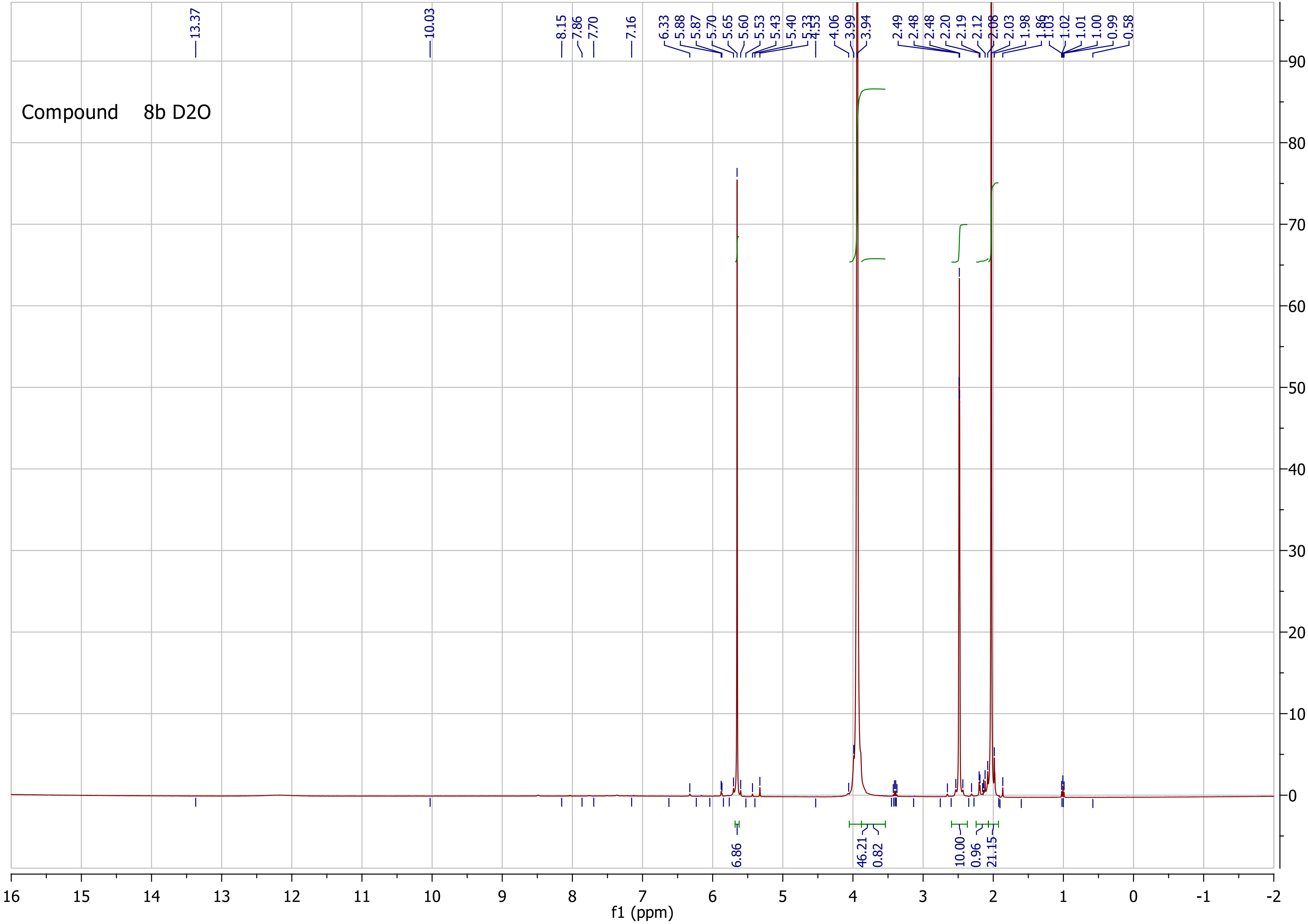
Compound 8b D2O
13.37
10.03
8.15
7.86
7.70
7.16
6.33
5.88
5.87
5.70
5.65
5.60
5.53
5.43
5.40
5.33
4.53
4.06
3.99
3.94
2.49
2.48
2.48
2.20
2.19
2.12
2.08
2.03
1.98
1.86
1.03
1.02
1.01
1.00
0.99
0.58
6.86
46.21
0.82
10.00
0.96
21.15
f1 (ppm)

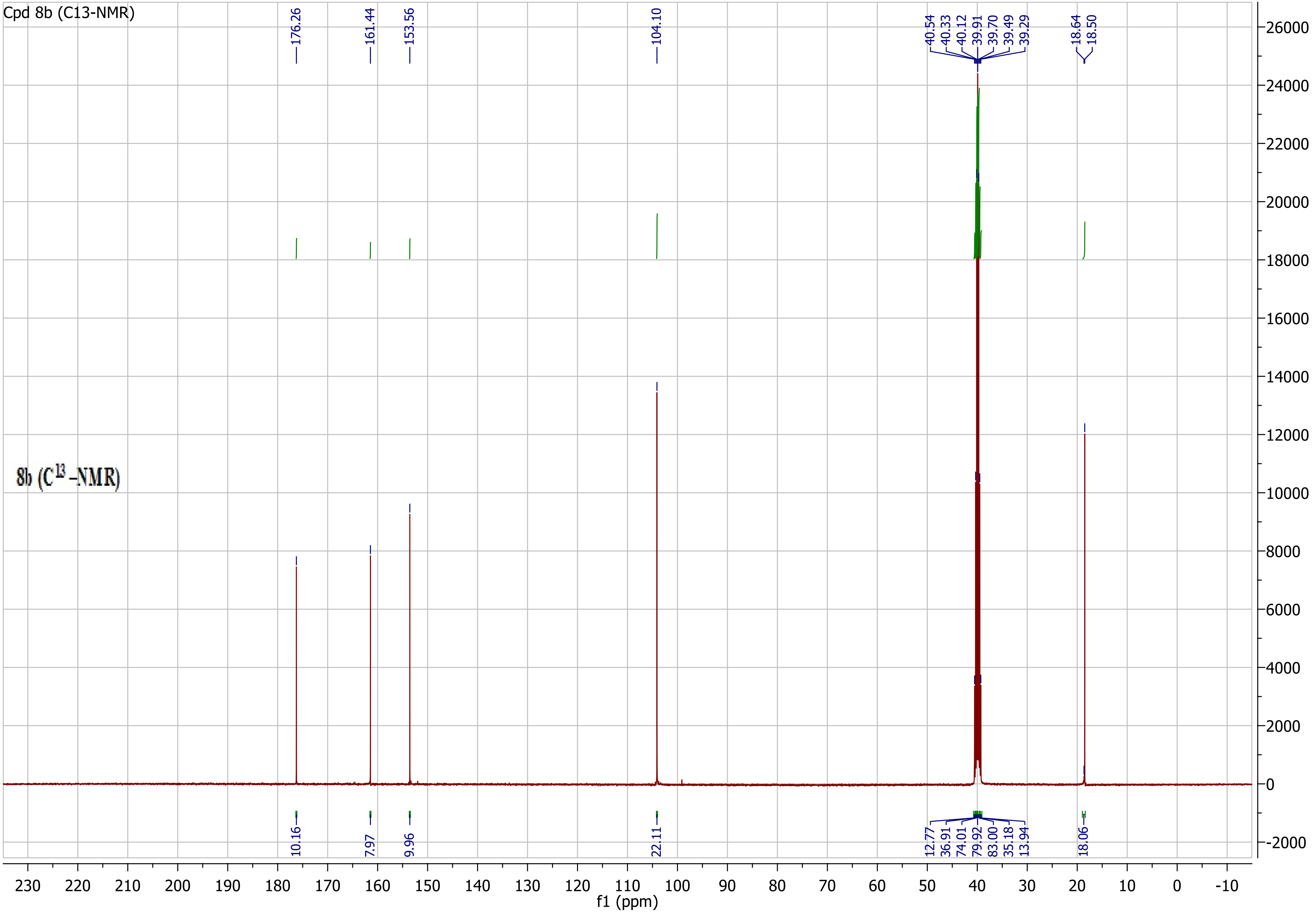
Cpd 8b (C13-NMR)
176.26
161.44
153.56
104.10
40.54
40.33
40.12
39.91
39.70
39.49
39.29
18.64
18.50
8b (C13-NMR)
10.16
7.97
9.96
22.11
12.77
36.91
74.01
79.92
83.00
35.18
13.94
18.06
f1 (ppm)

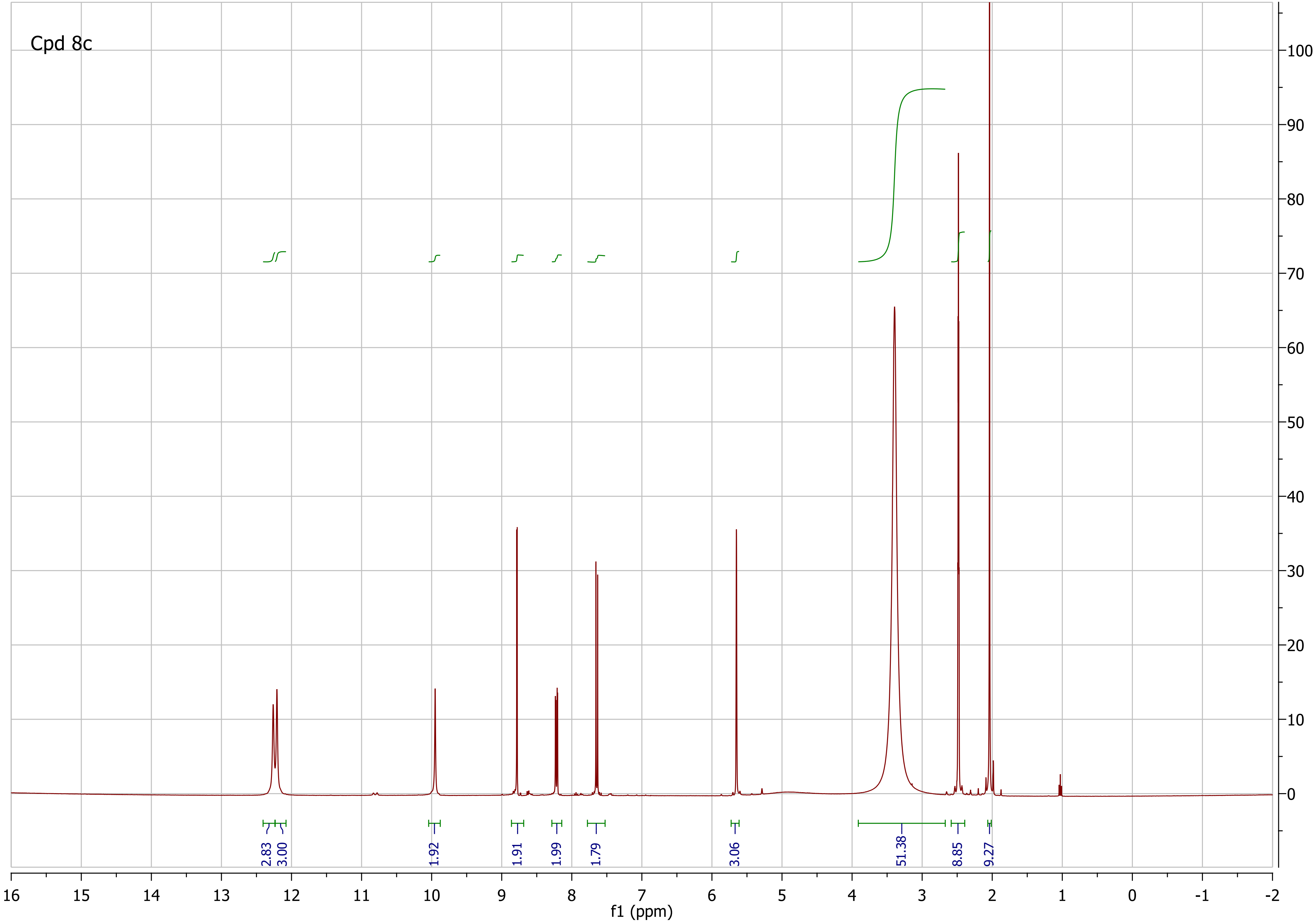

Cpd 8c
2.83
3.00
1.92
1.91
1.99
1.79
3.06
51.38
8.85
9.27
f1 (ppm)

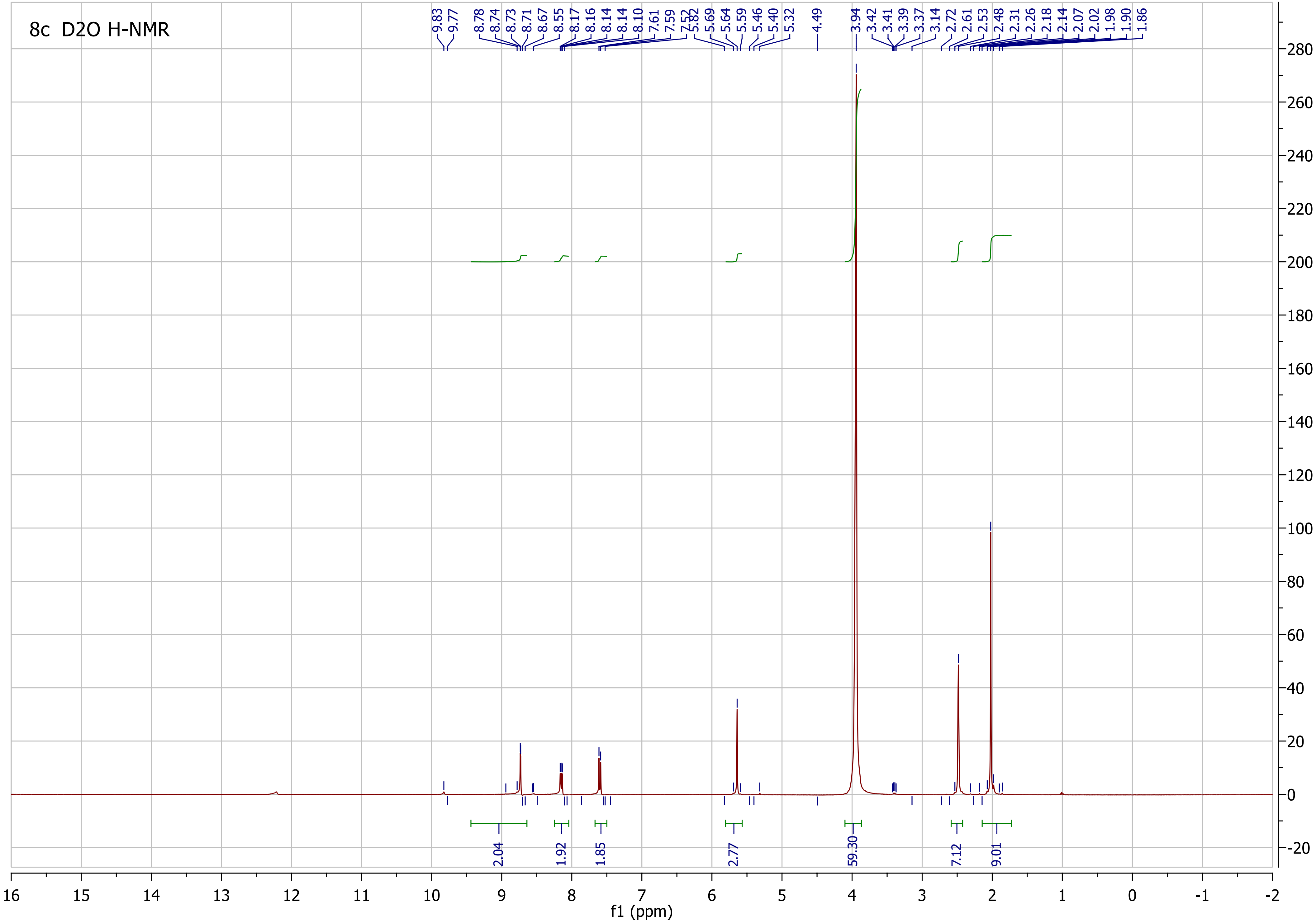
8c D2O H-NMR
9.83
9.77
8.78
8.74
8.73
8.71
8.67
8.55
8.17
8.16
8.14
8.14
8.10
7.61
7.59
7.52
5.82
5.69
5.64
5.59
5.46
5.40
5.32
4.49
3.94
3.42
3.41
3.39
3.37
3.14
2.72
2.61
2.53
2.48
2.31
2.26
2.18
2.14
2.07
2.02
1.98
1.90
1.86
2.04
1.92
1.85
2.77
59.30
7.12
9.01
f1 (ppm)

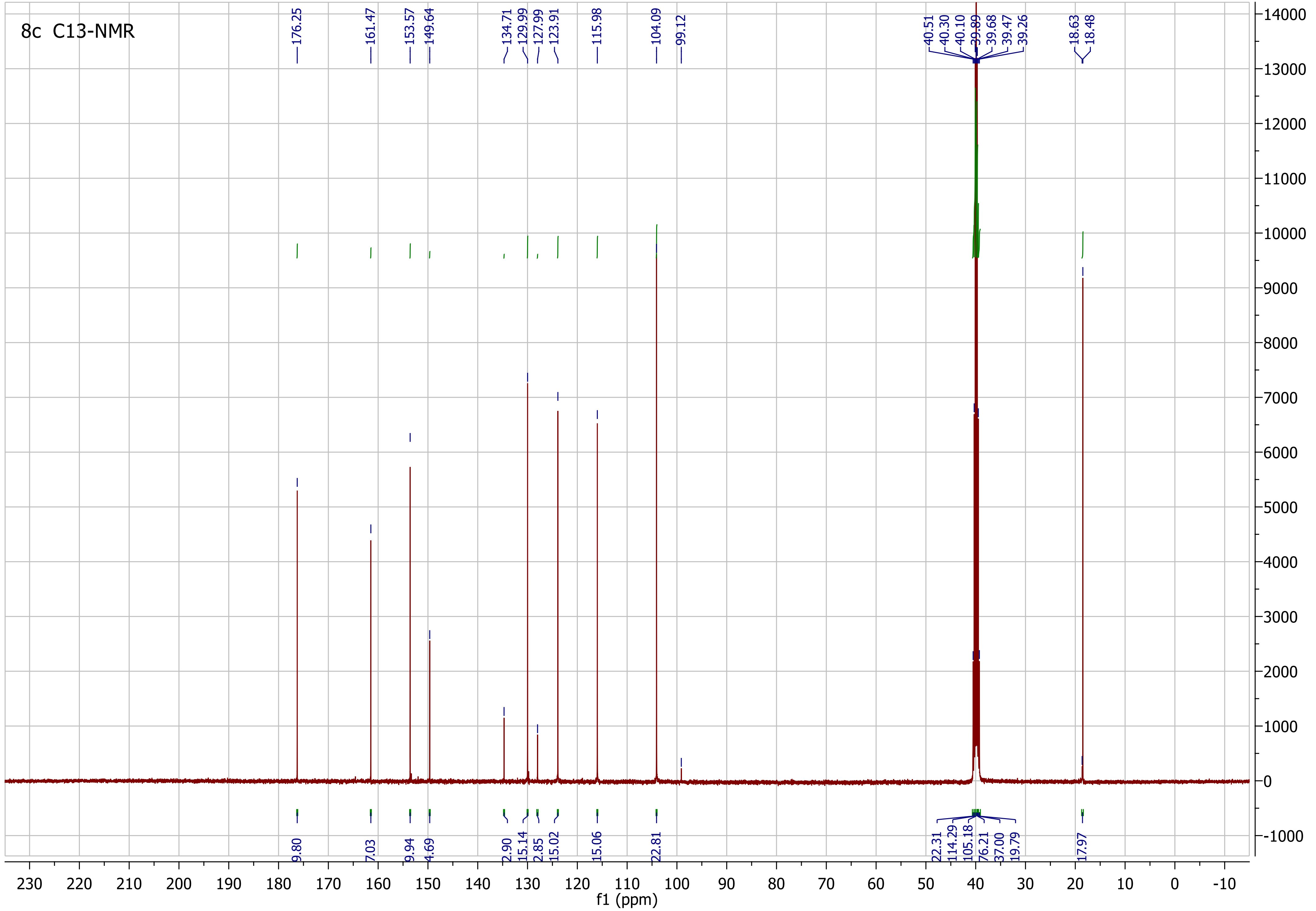

8c C13-NMR
176.25
161.47
153.57
149.64
134.71
129.99
127.99
123.91
115.98
104.09
99.12
40.51
40.30
40.10
39.89
39.68
39.47
39.26
18.63
18.48
9.80
7.03
9.94
4.69
2.90
15.14
2.85
15.02
15.06
22.81
22.31
114.29
105.18
76.21
37.00
19.79
17.97
14000
13000
12000
11000
10000
9000
8000
7000
6000
5000
4000
3000
2000
1000
0
-1000
230
220
210
200
190
180
170
160
150
140
130
120
110
100
90
80
70
60
50
40
30
20
10
0
-10
f1 (ppm)

Cpd 9a

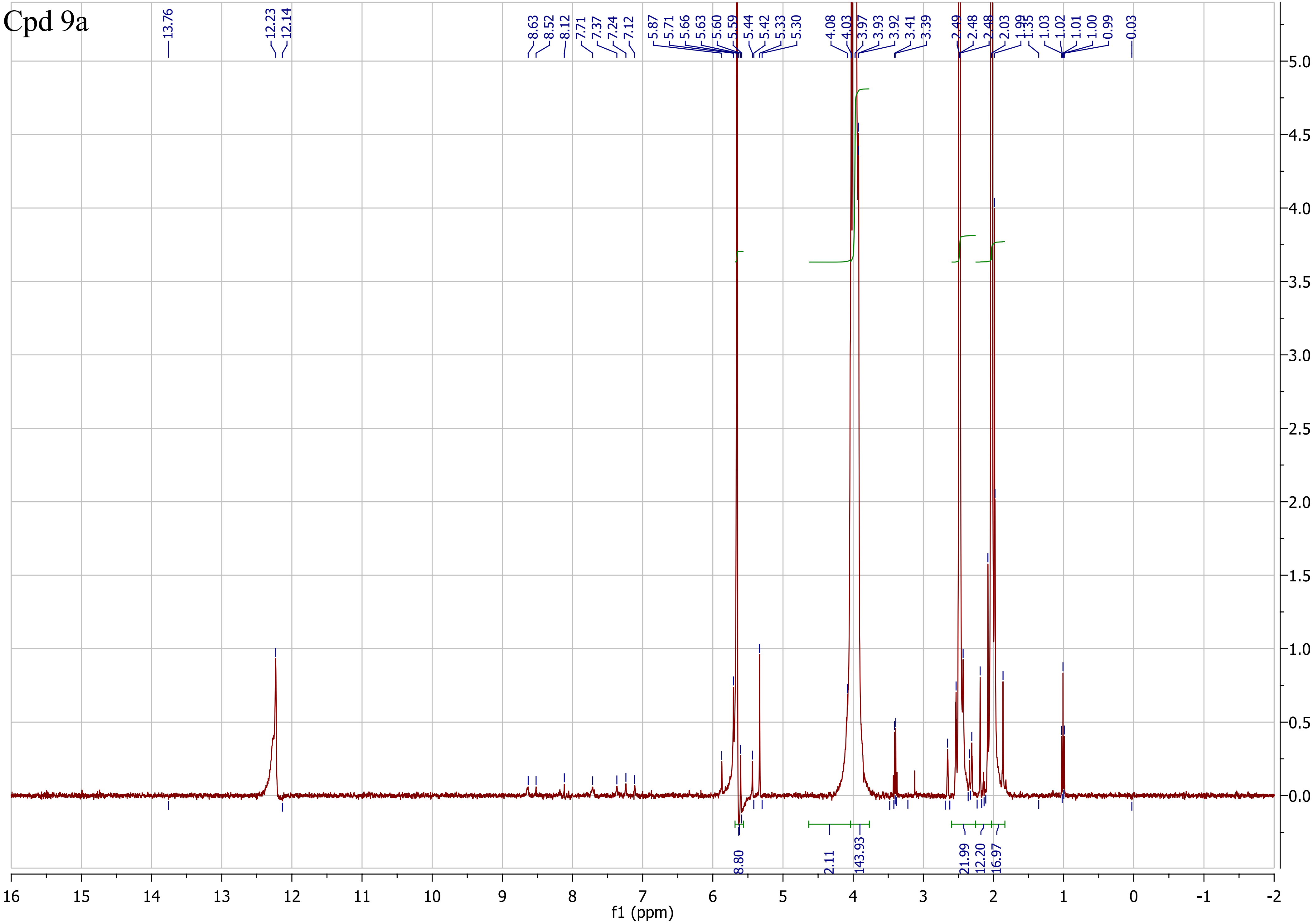
13.76
12.23
12.14
8.63
8.52
8.12
7.71
7.37
7.24
7.12
5.87
5.71
5.66
5.63
5.60
5.59
5.44
5.42
5.33
5.30
4.08
4.03
3.97
3.93
3.92
3.41
3.39
2.49
2.48
2.48
2.03
1.99
1.35
1.03
1.02
1.01
1.00
0.99
0.03
8.80
2.11
143.93
21.99
12.20
16.97
f1 (ppm)

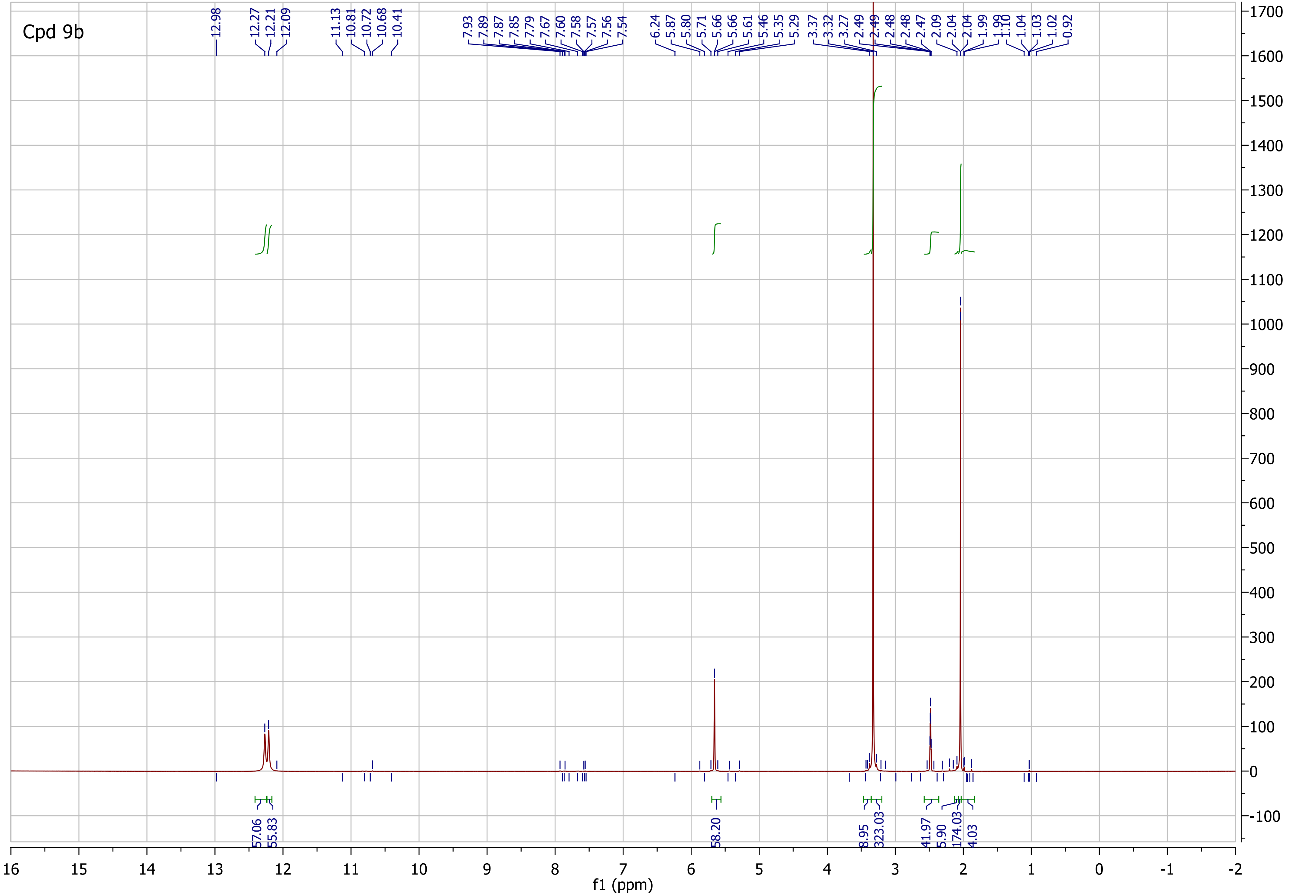
Cpd 9b
12.98
12.27
12.21
12.09
11.13
10.81
10.72
10.68
10.41
7.93
7.89
7.87
7.85
7.79
7.67
7.60
7.58
7.57
7.56
7.54
6.24
5.87
5.80
5.71
5.66
5.66
5.61
5.46
5.35
5.29
3.37
3.32
3.27
2.49
2.49
2.48
2.48
2.47
2.09
2.04
2.04
1.99
1.10
1.04
1.03
1.02
0.92
57.06
55.83
58.20
8.95
323.03
41.97
5.90
174.03
4.03
1700
1600
1500
1400
1300
1200
1100
1000
900
800
700
600
500
400
300
200
100
0
-100
16
15
14
13
12
11
10
9
8
7
6
5
4
3
2
1
0
-1
-2
f1 (ppm)

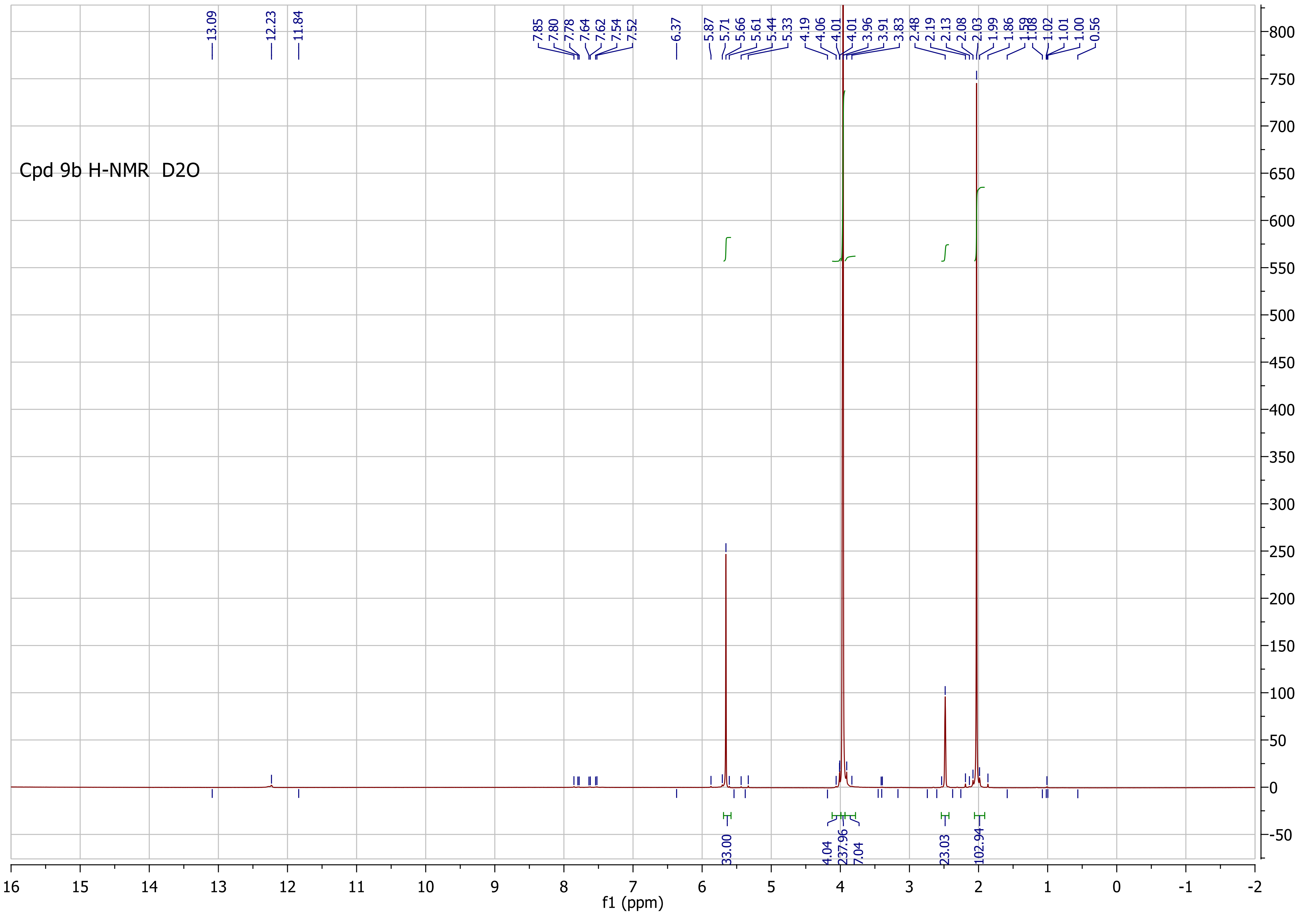
Cpd 9b H-NMR D2O
13.09
12.23
11.84
7.85
7.80
7.78
7.64
7.62
7.54
7.52
6.37
5.87
5.71
5.66
5.61
5.44
5.33
4.19
4.06
4.01
4.01
3.96
3.91
3.83
2.48
2.19
2.13
2.08
2.03
1.99
1.86
1.59
1.08
1.02
1.01
1.00
0.56
33.00
4.04
237.96
7.04
23.03
102.94
f1 (ppm)

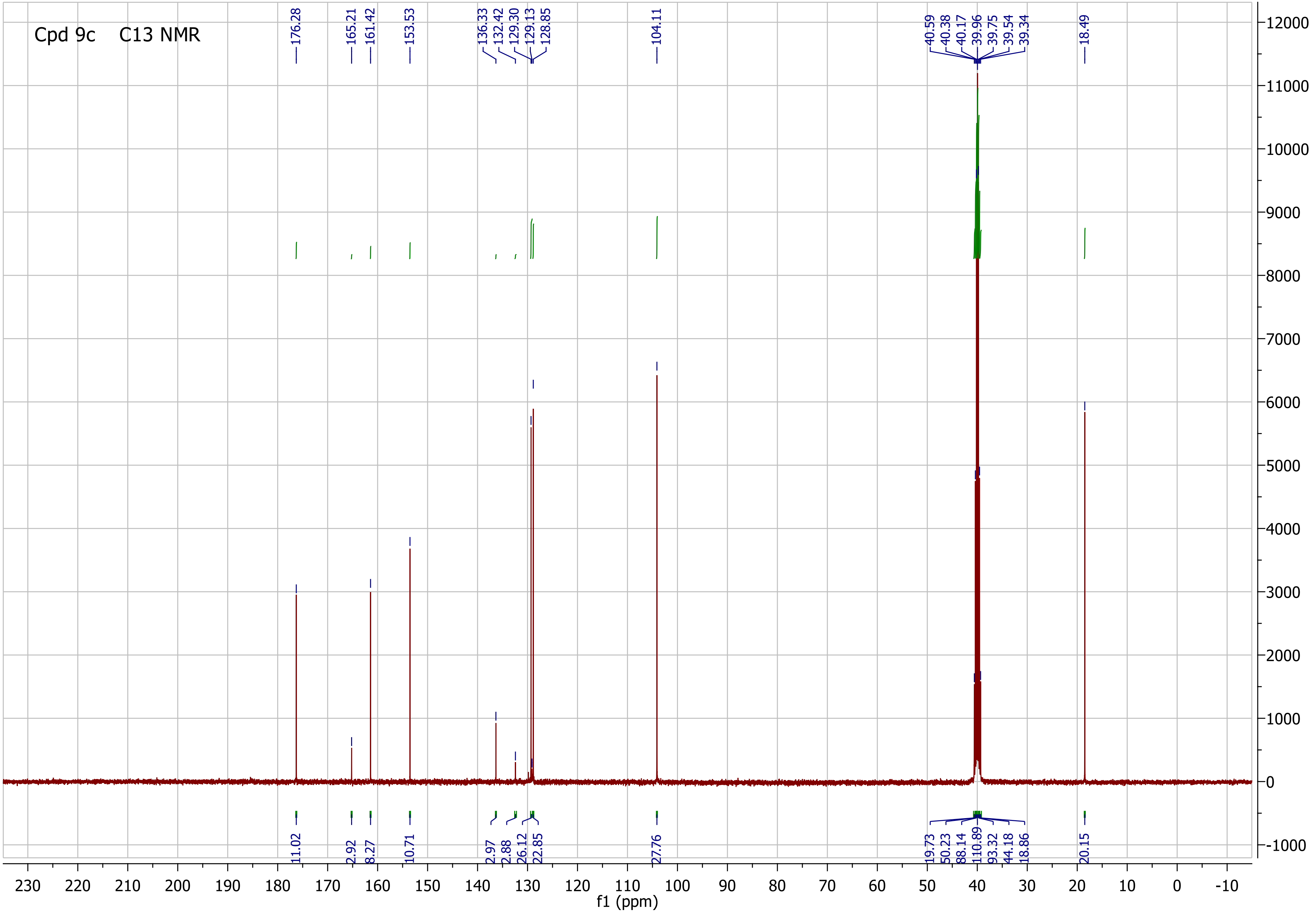
Cpd 9c C13 NMR
176.28
165.21
161.42
153.53
136.33
132.42
129.30
129.13
128.85
104.11
40.59
40.38
40.17
39.96
39.75
39.54
39.34
18.49
11.02
2.92
8.27
10.71
2.97
2.88
26.12
22.85
27.76
19.73
50.23
88.14
110.89
93.32
44.18
18.86
20.15
f1 (ppm)

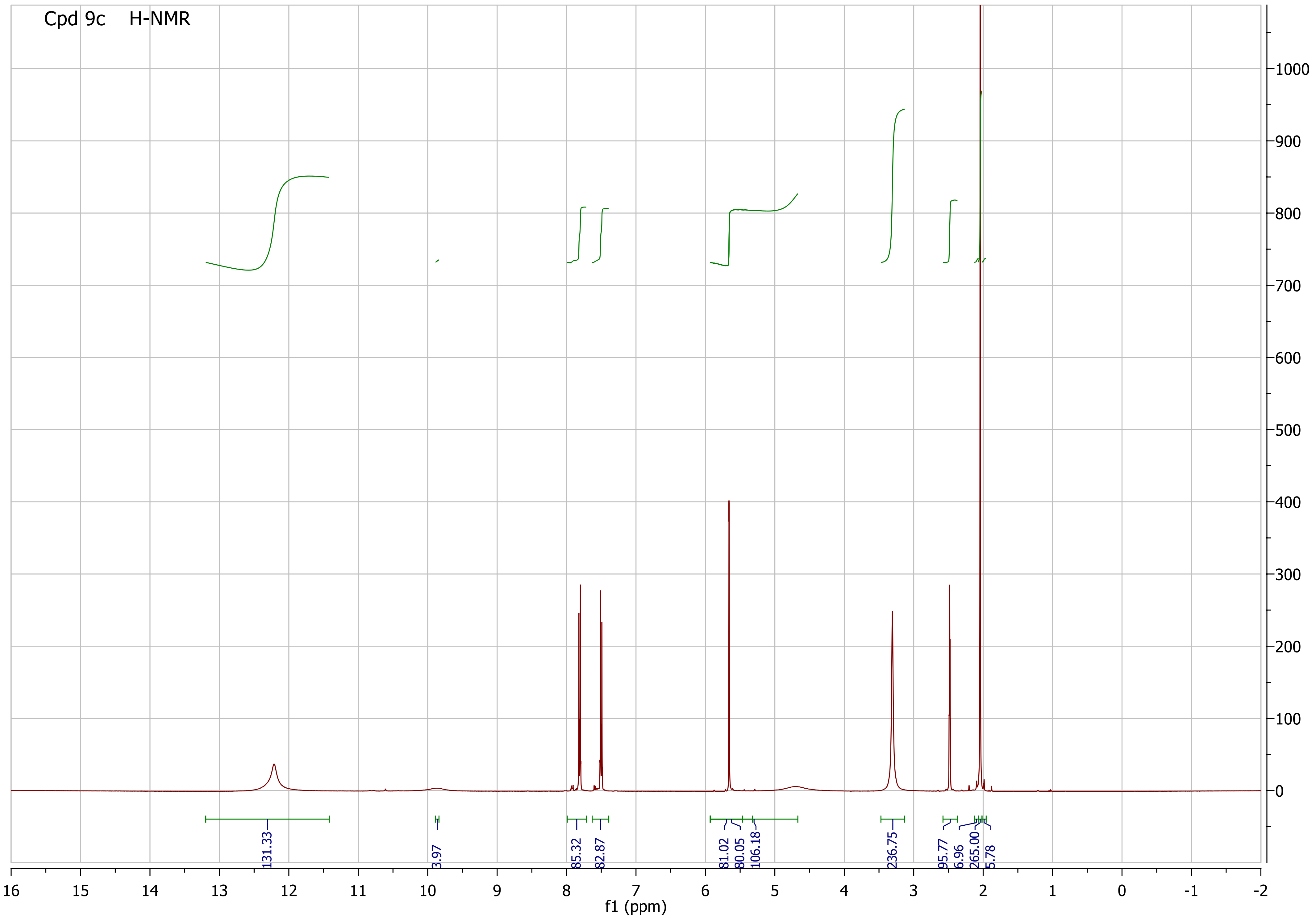
Cpd 9c H-NMR
131.33
3.97
85.32
82.87
81.02
80.05
106.18
236.75
95.77
6.96
265.00
5.78
16
15
14
13
12
11
10
9
8
7
6
5
4
3
2
1
0
-1
-2
f1 (ppm)
1000
900
800
700
600
500
400
300
200
100
0
